# Supplementary figures and images for: SUN2 mediates calcium-triggered nuclear actin polymerization to cluster active RNA polymerase II
Source: EMBO Rep. 2024 Sep 24;25(11):9. doi: 10.1038/s44319-024-00274-8 (PMC11549082; doi:10.1038/s44319-024-00274-8)

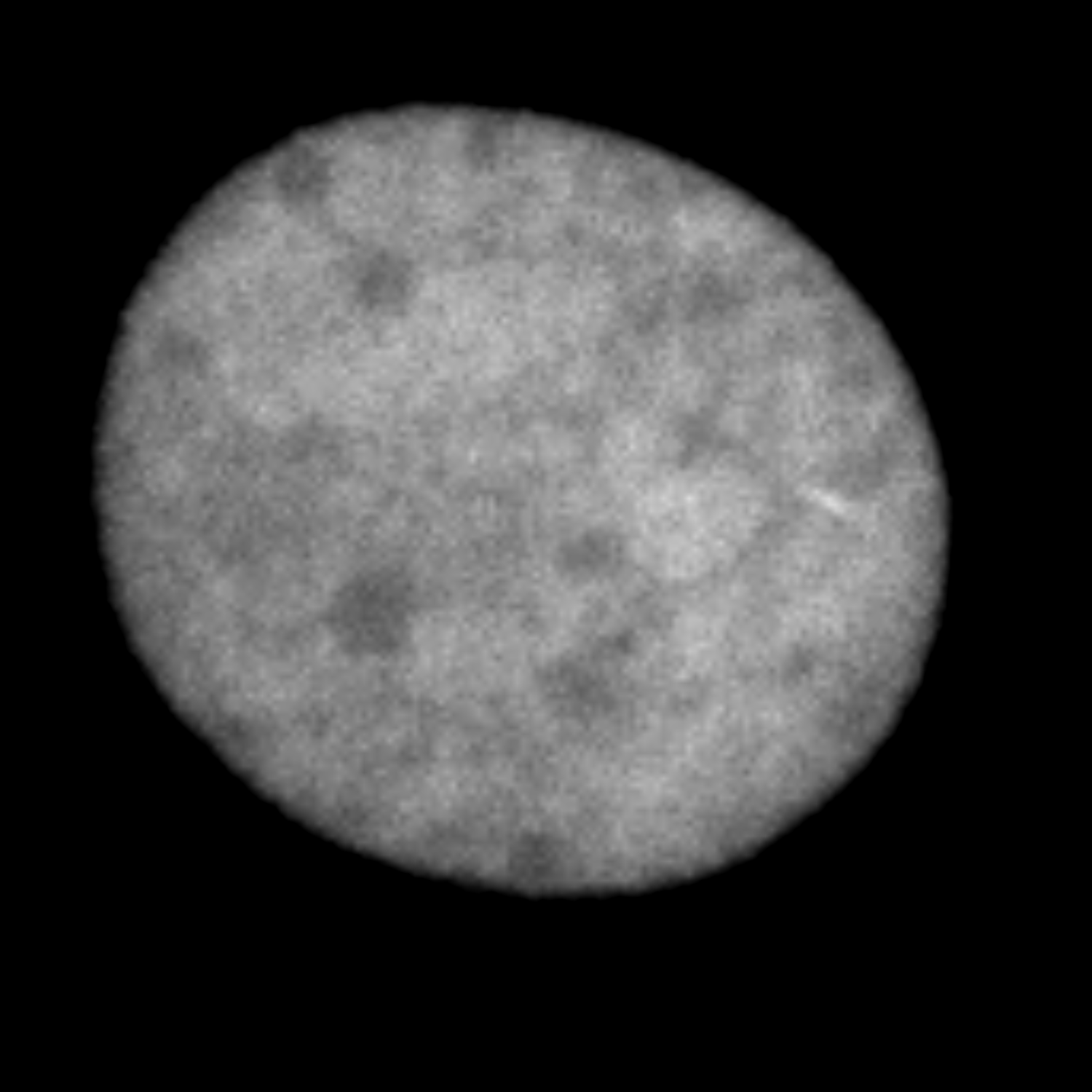

Supplement: Supplementary file 5 — Source data Fig. 1 [file 44319_2024_274_MOESM5_ESM.zip › Figure 1/1A/0 sec.tif]

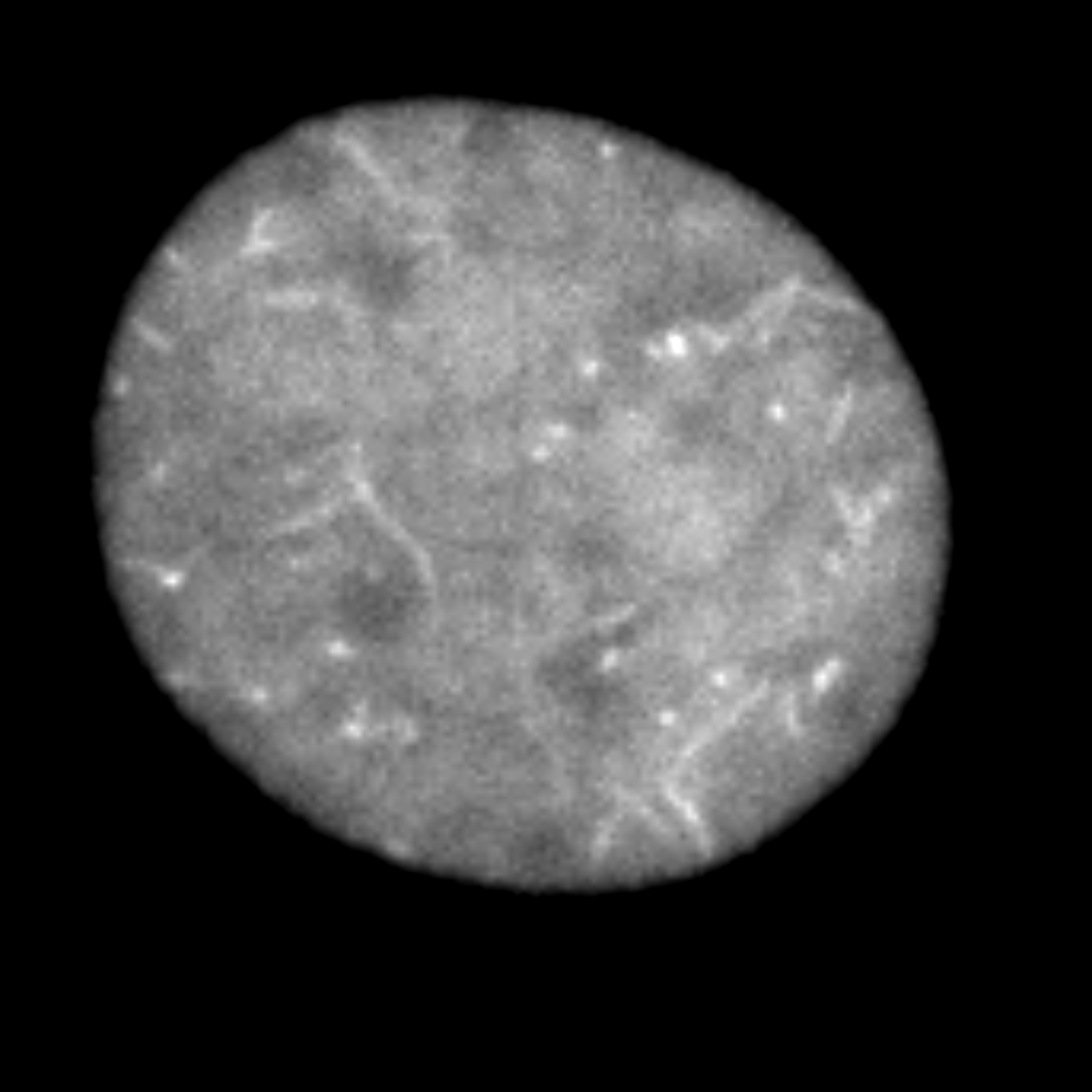

Supplement: Supplementary file 5 — Source data Fig. 1 [file 44319_2024_274_MOESM5_ESM.zip › Figure 1/1A/10 sec.tif]

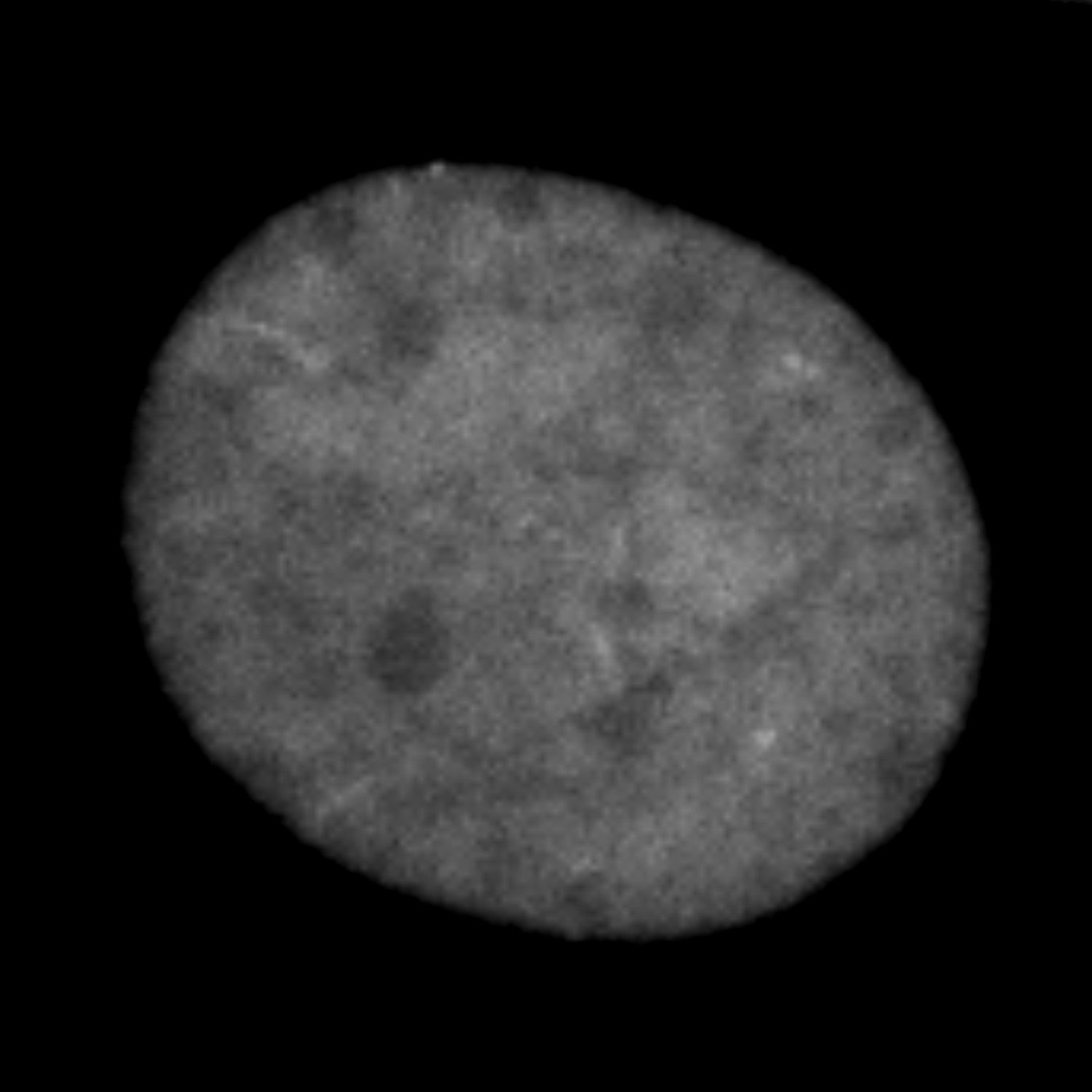

Supplement: Supplementary file 5 — Source data Fig. 1 [file 44319_2024_274_MOESM5_ESM.zip › Figure 1/1A/240 sec.tif]

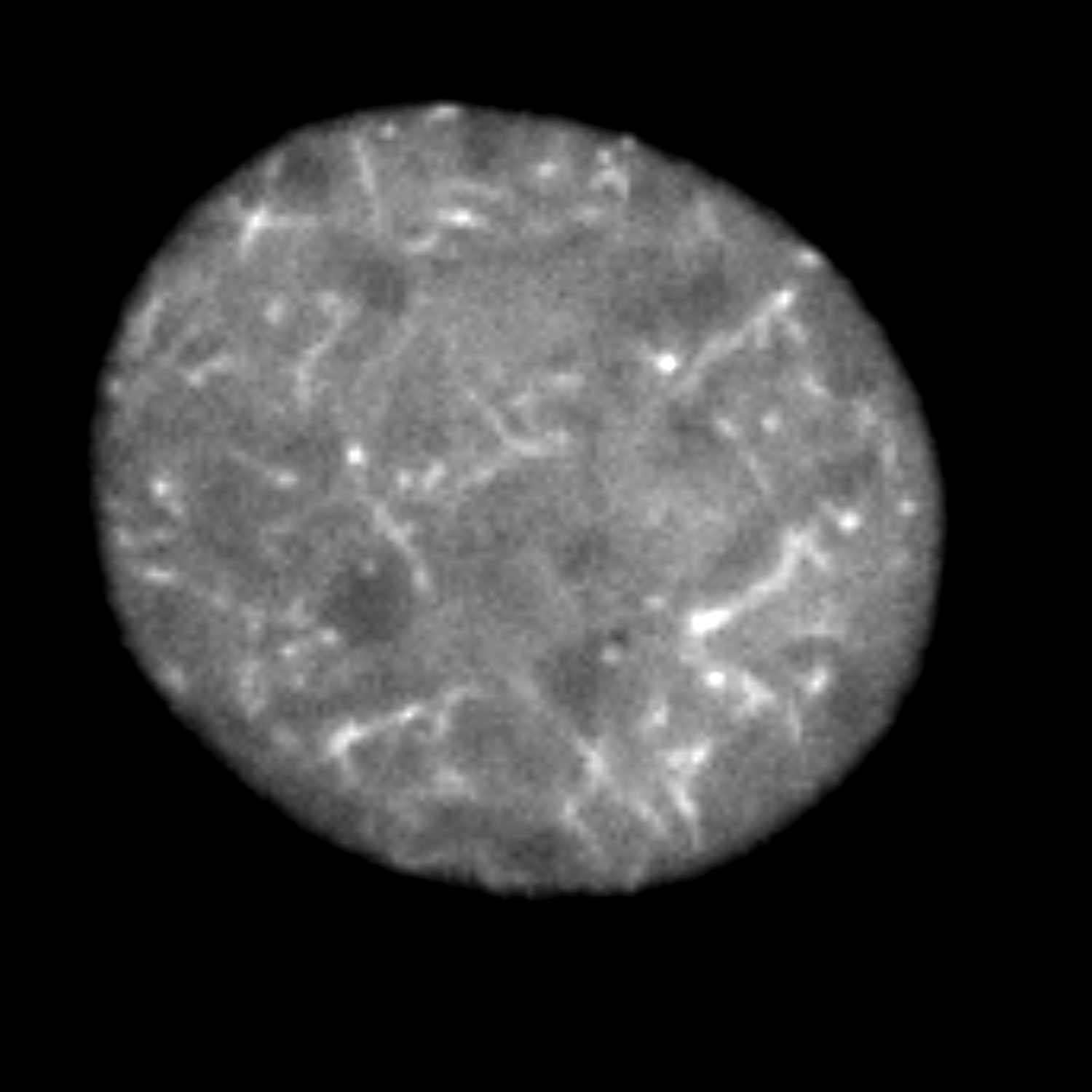

Supplement: Supplementary file 5 — Source data Fig. 1 [file 44319_2024_274_MOESM5_ESM.zip › Figure 1/1A/40 sec.tif]

WB Figure 1E

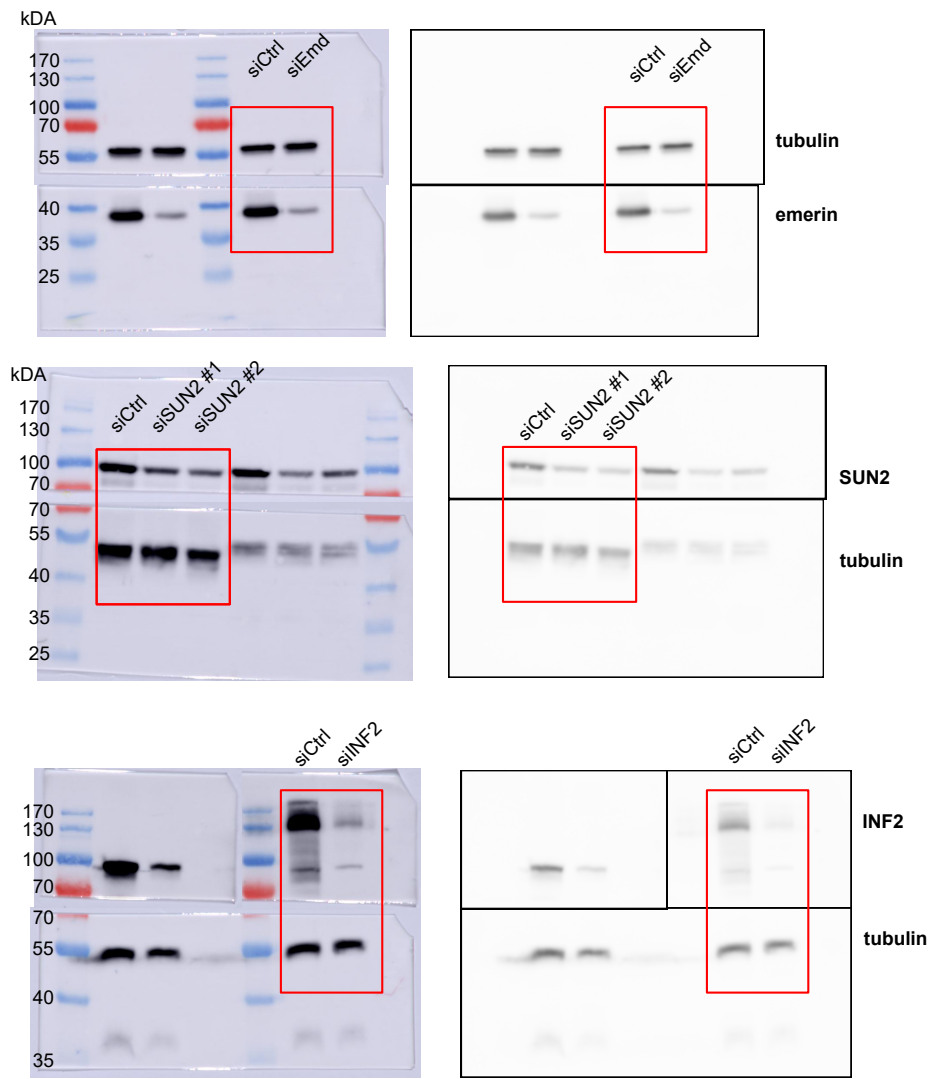

Supplement: Supplementary file 5 — Source data Fig. 1 [file 44319_2024_274_MOESM5_ESM.zip › Figure 1/1E/1E_WB.pdf]

WB Figure 1F

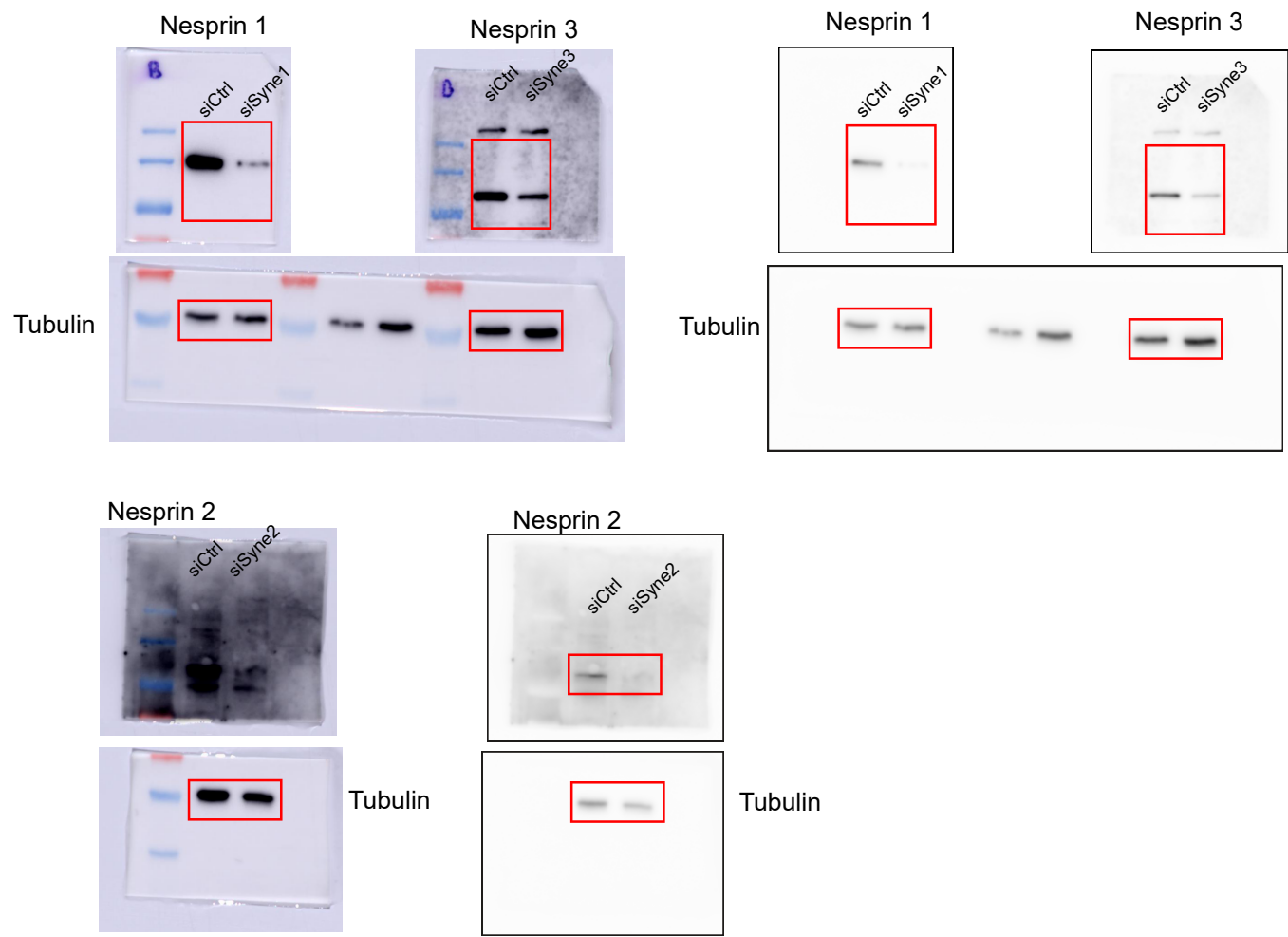

Supplement: Supplementary file 5 — Source data Fig. 1 [file 44319_2024_274_MOESM5_ESM.zip › Figure 1/1F/1F_WB.pdf]

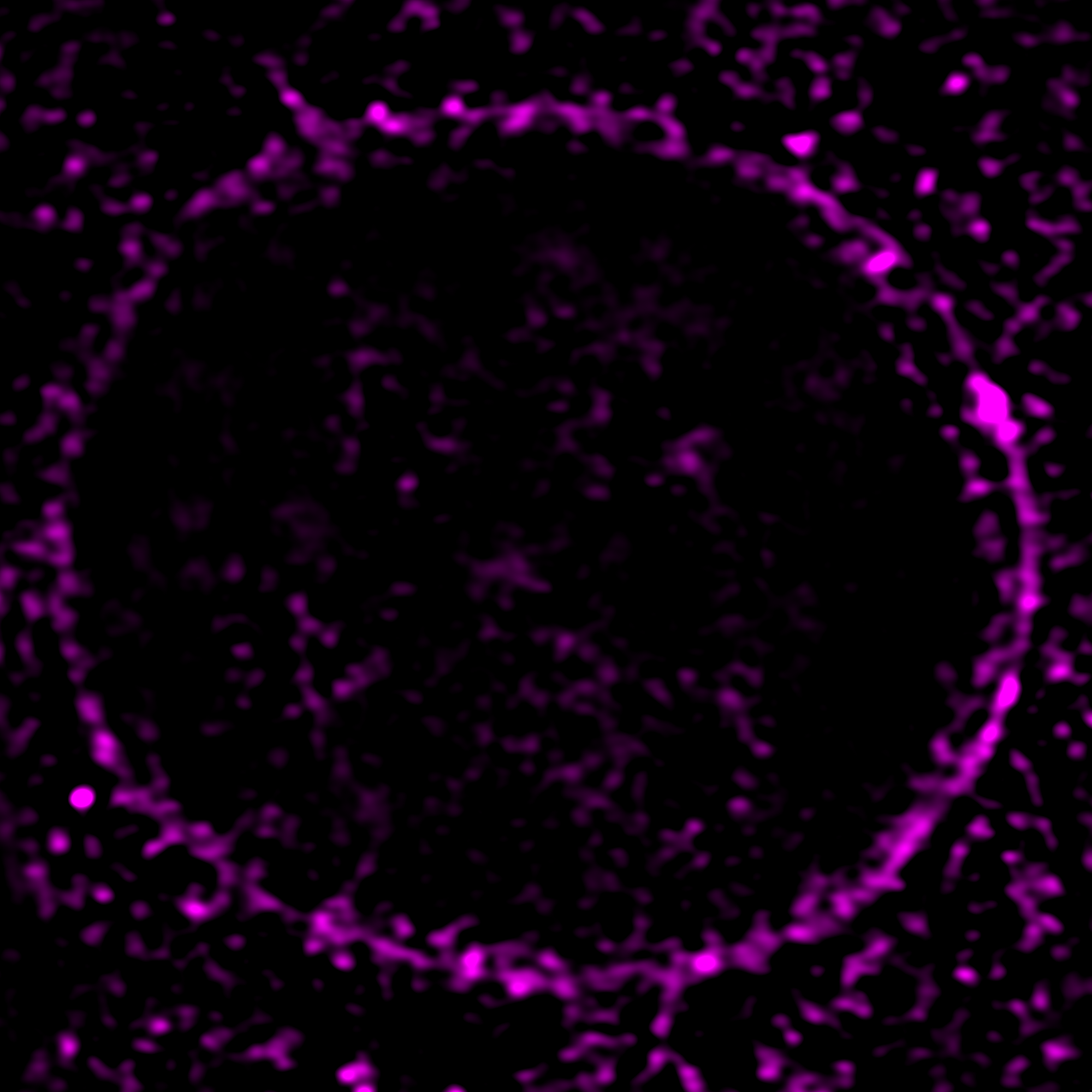

Supplement: Supplementary file 6 — Source data Fig. 2 [file 44319_2024_274_MOESM6_ESM.zip › Figure 2/2A/INF2-647.tif]

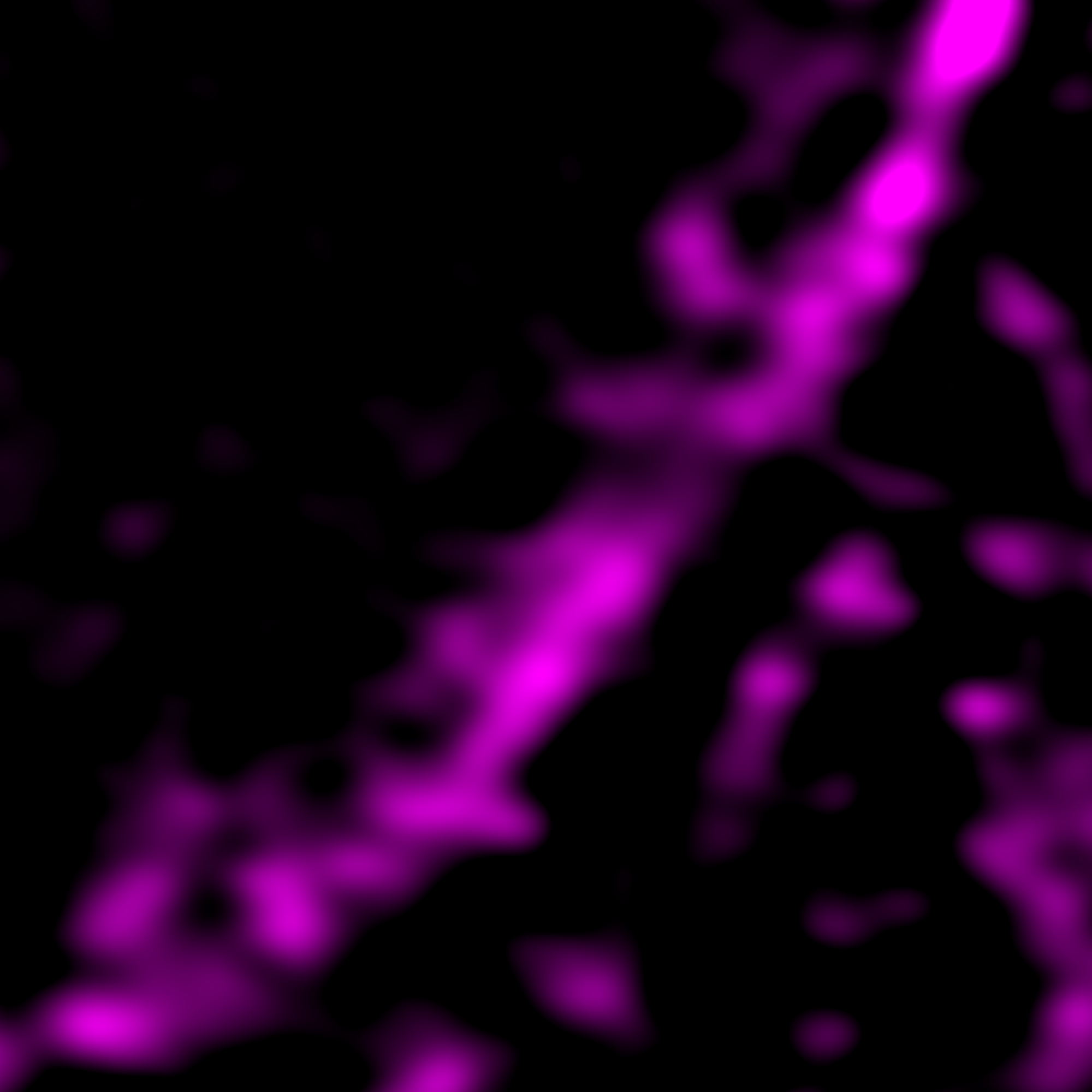

Supplement: Supplementary file 6 — Source data Fig. 2 [file 44319_2024_274_MOESM6_ESM.zip › Figure 2/2A/INF2-647_zoom.tif]

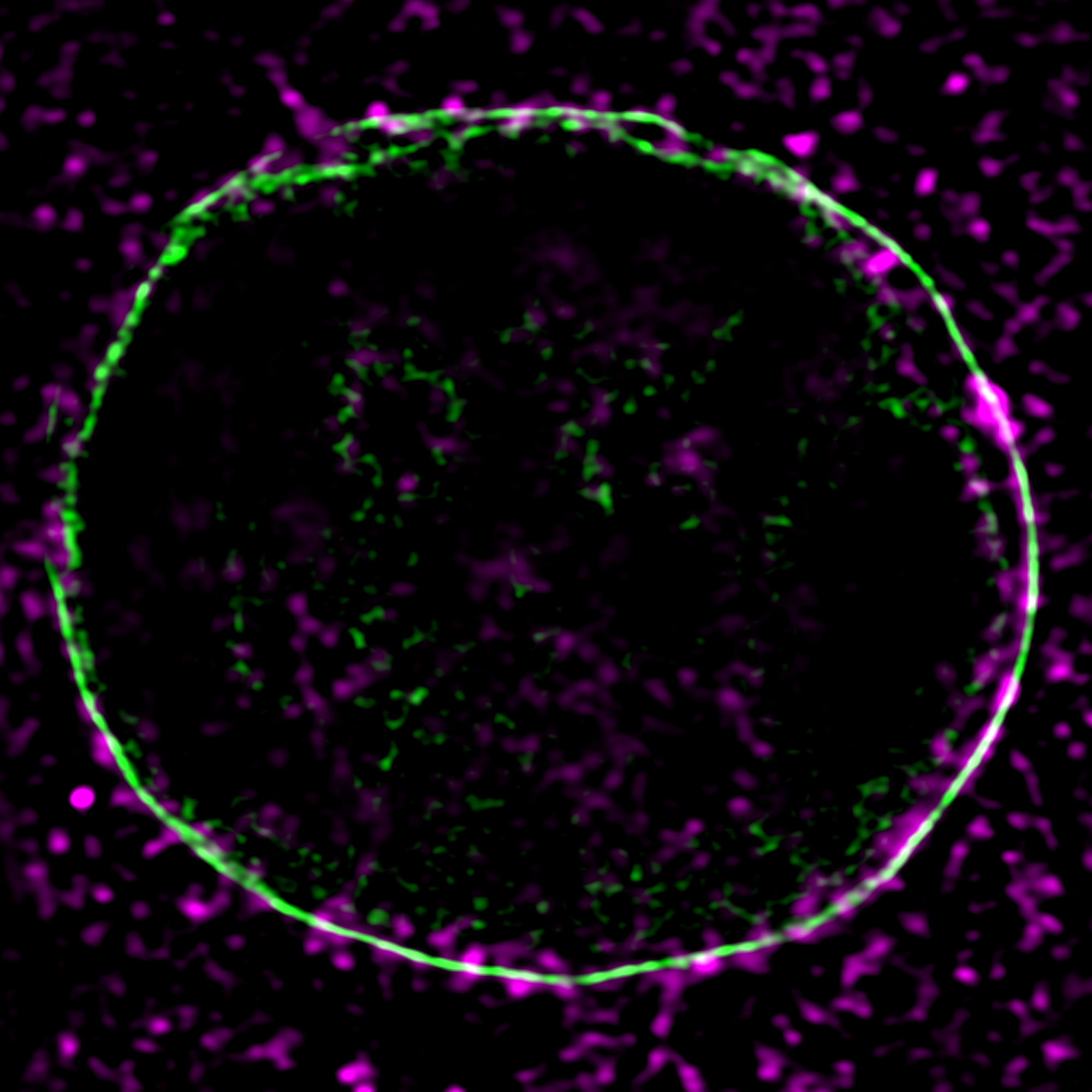

Supplement: Supplementary file 6 — Source data Fig. 2 [file 44319_2024_274_MOESM6_ESM.zip › Figure 2/2A/merge.tif]

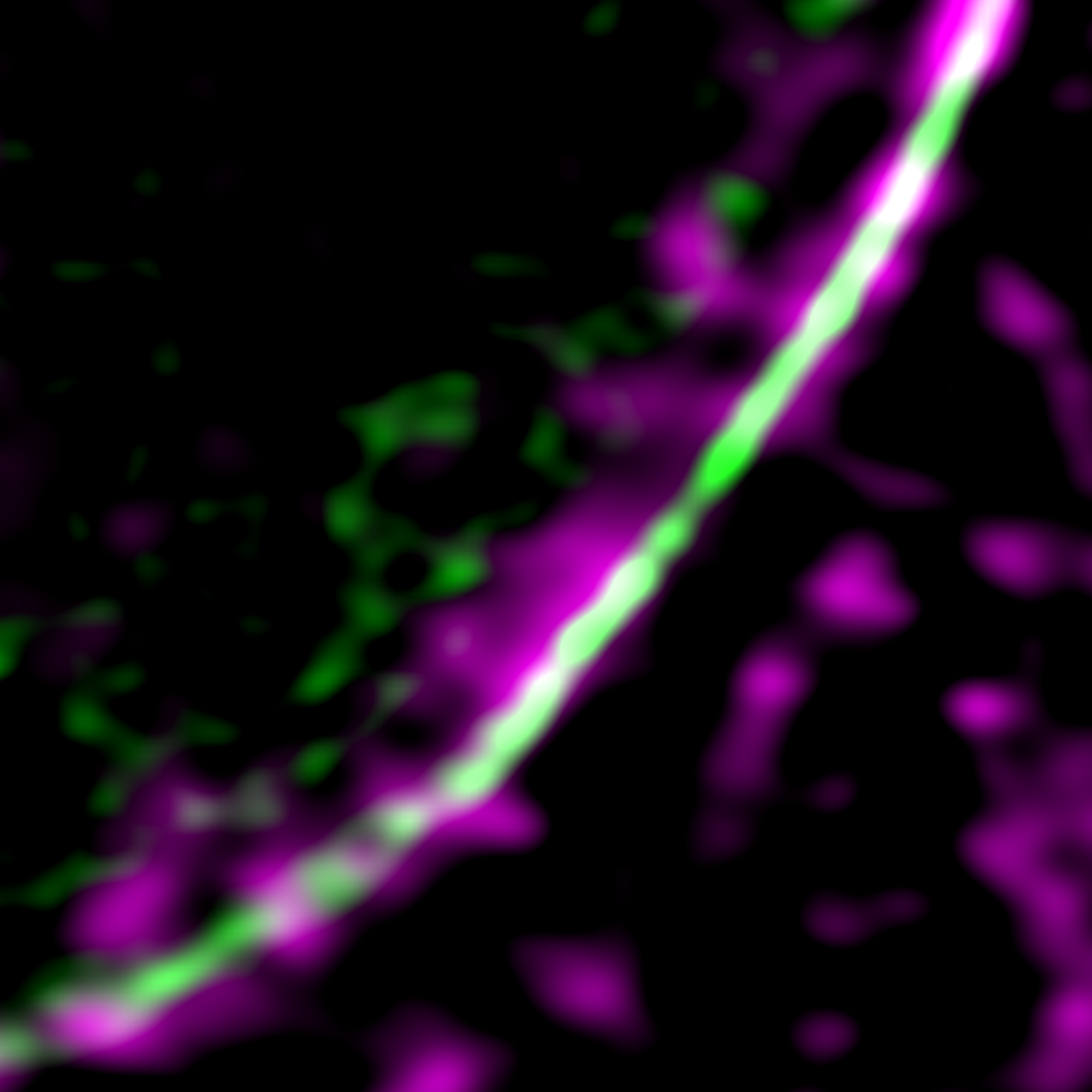

Supplement: Supplementary file 6 — Source data Fig. 2 [file 44319_2024_274_MOESM6_ESM.zip › Figure 2/2A/merge_zoom.tif]

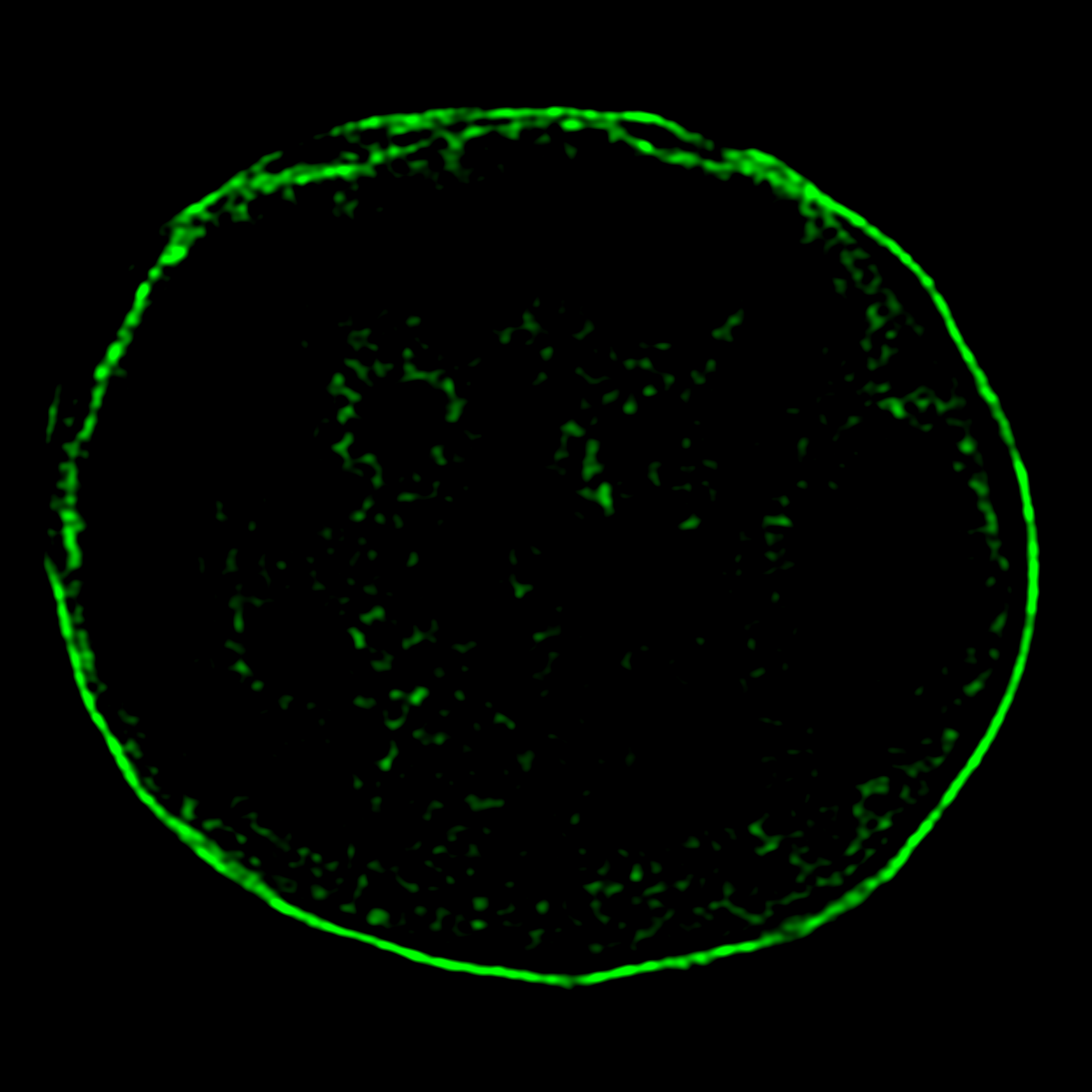

Supplement: Supplementary file 6 — Source data Fig. 2 [file 44319_2024_274_MOESM6_ESM.zip › Figure 2/2A/SUN2-488.tif]

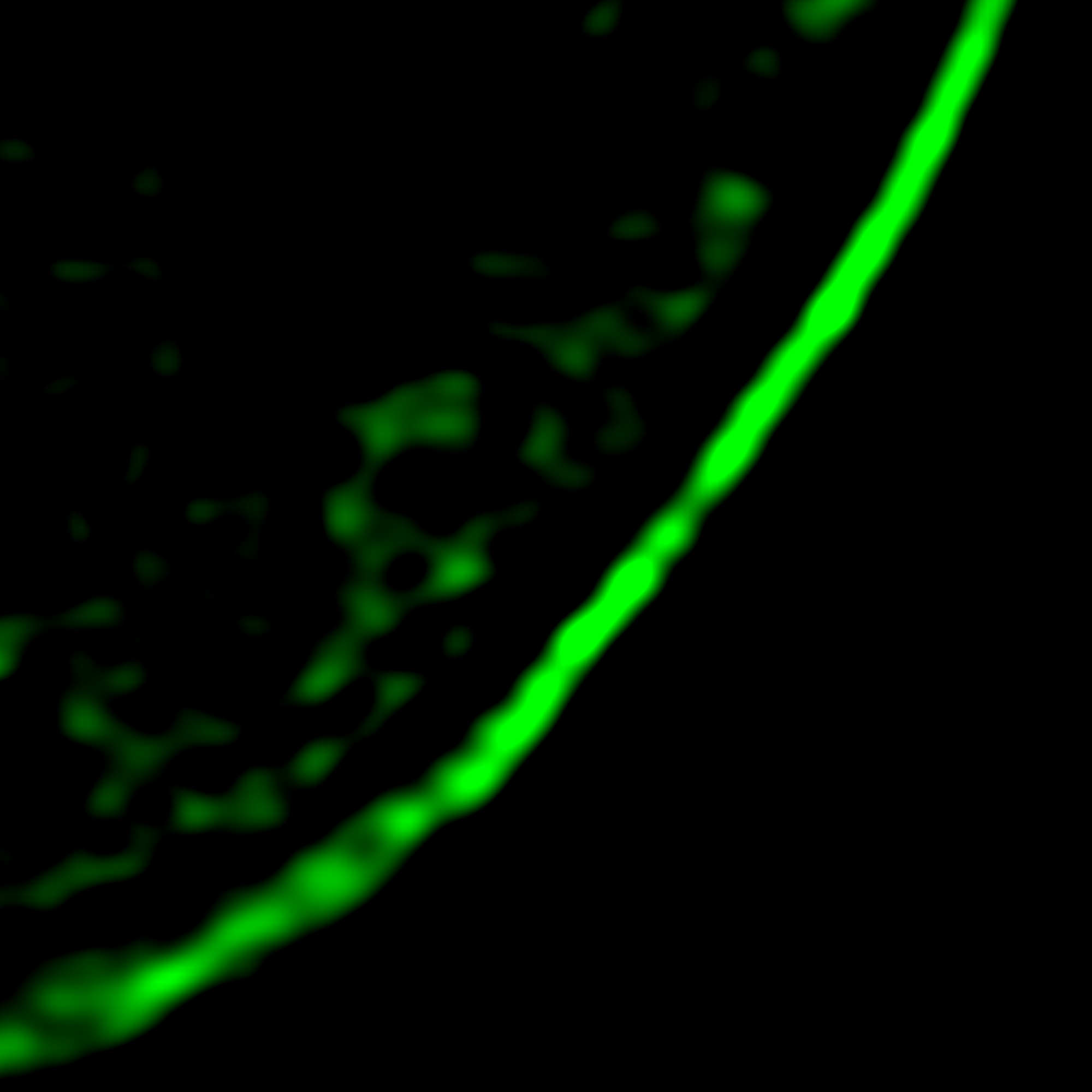

Supplement: Supplementary file 6 — Source data Fig. 2 [file 44319_2024_274_MOESM6_ESM.zip › Figure 2/2A/SUN2-488_zoom.tif]

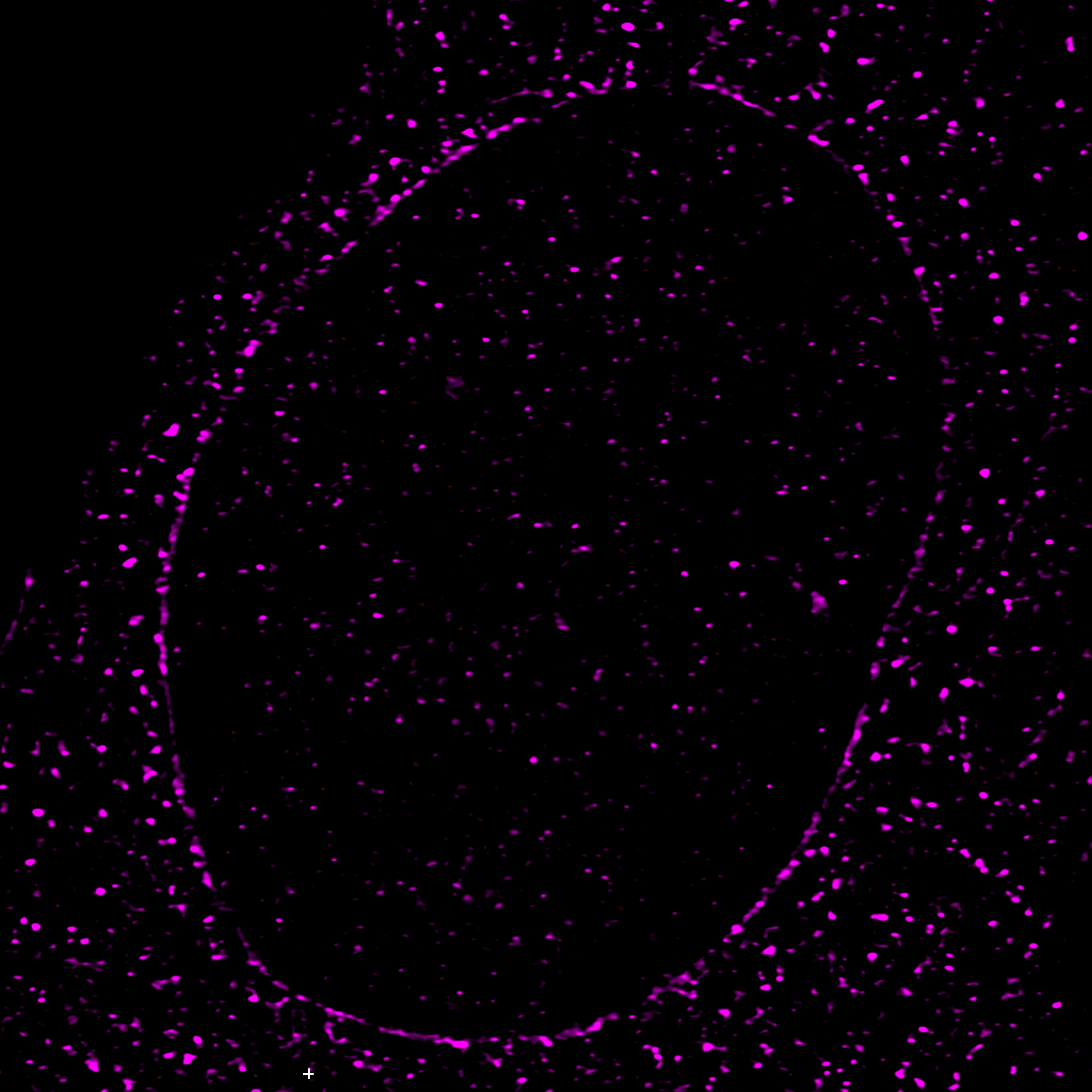

Supplement: Supplementary file 6 — Source data Fig. 2 [file 44319_2024_274_MOESM6_ESM.zip › Figure 2/2B/INF2-647.tif]

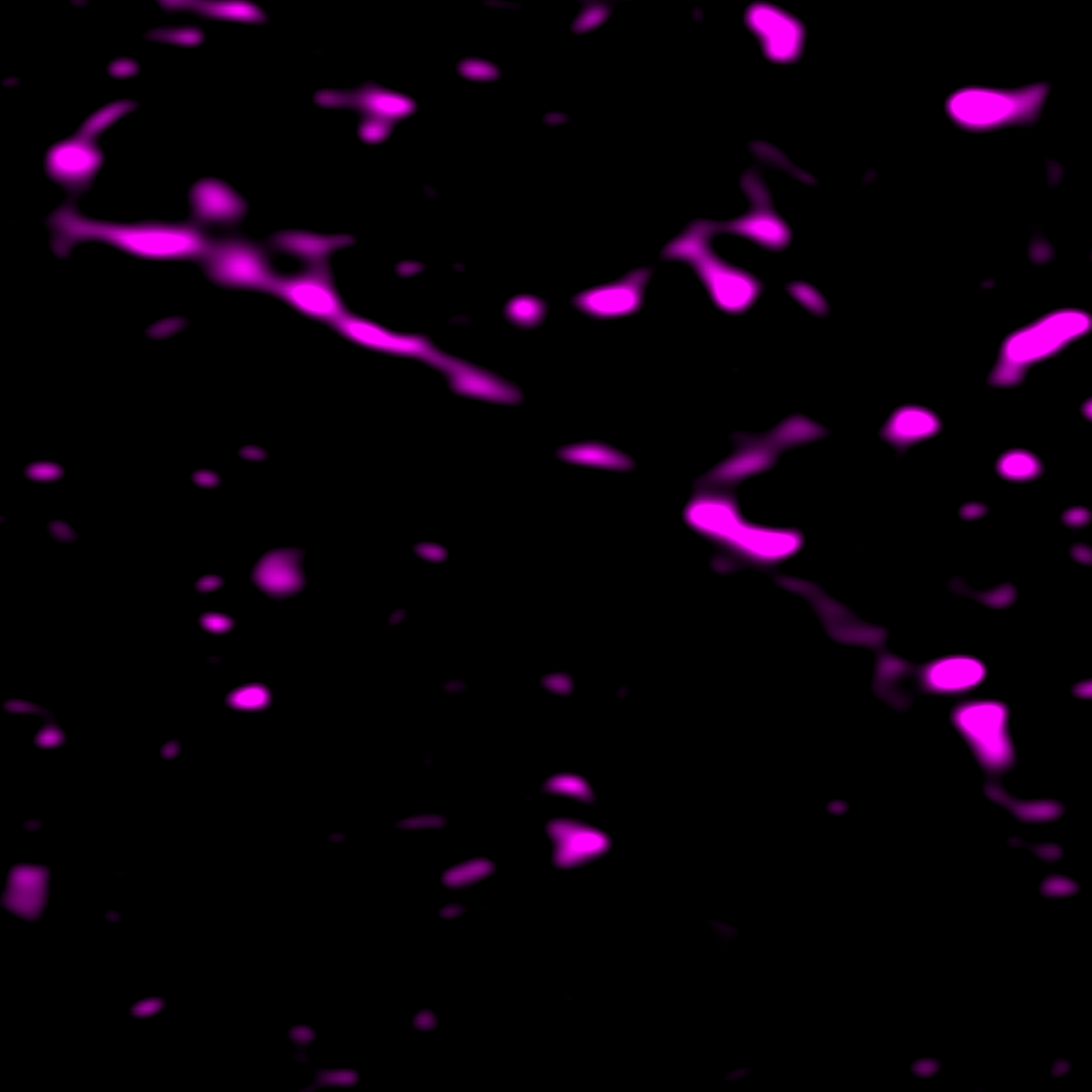

Supplement: Supplementary file 6 — Source data Fig. 2 [file 44319_2024_274_MOESM6_ESM.zip › Figure 2/2B/INF2-647_zoom.tif]

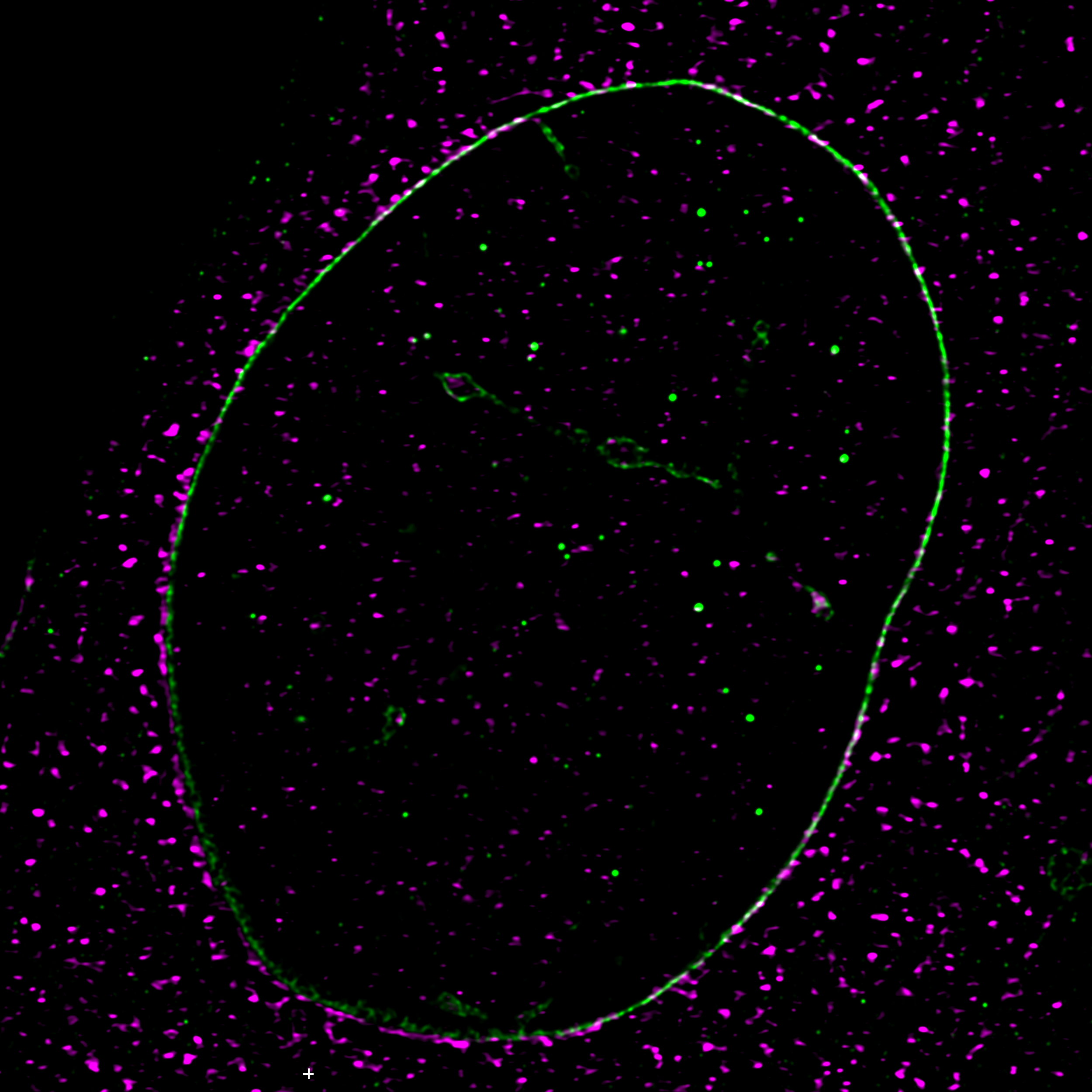

Supplement: Supplementary file 6 — Source data Fig. 2 [file 44319_2024_274_MOESM6_ESM.zip › Figure 2/2B/merge.tif]

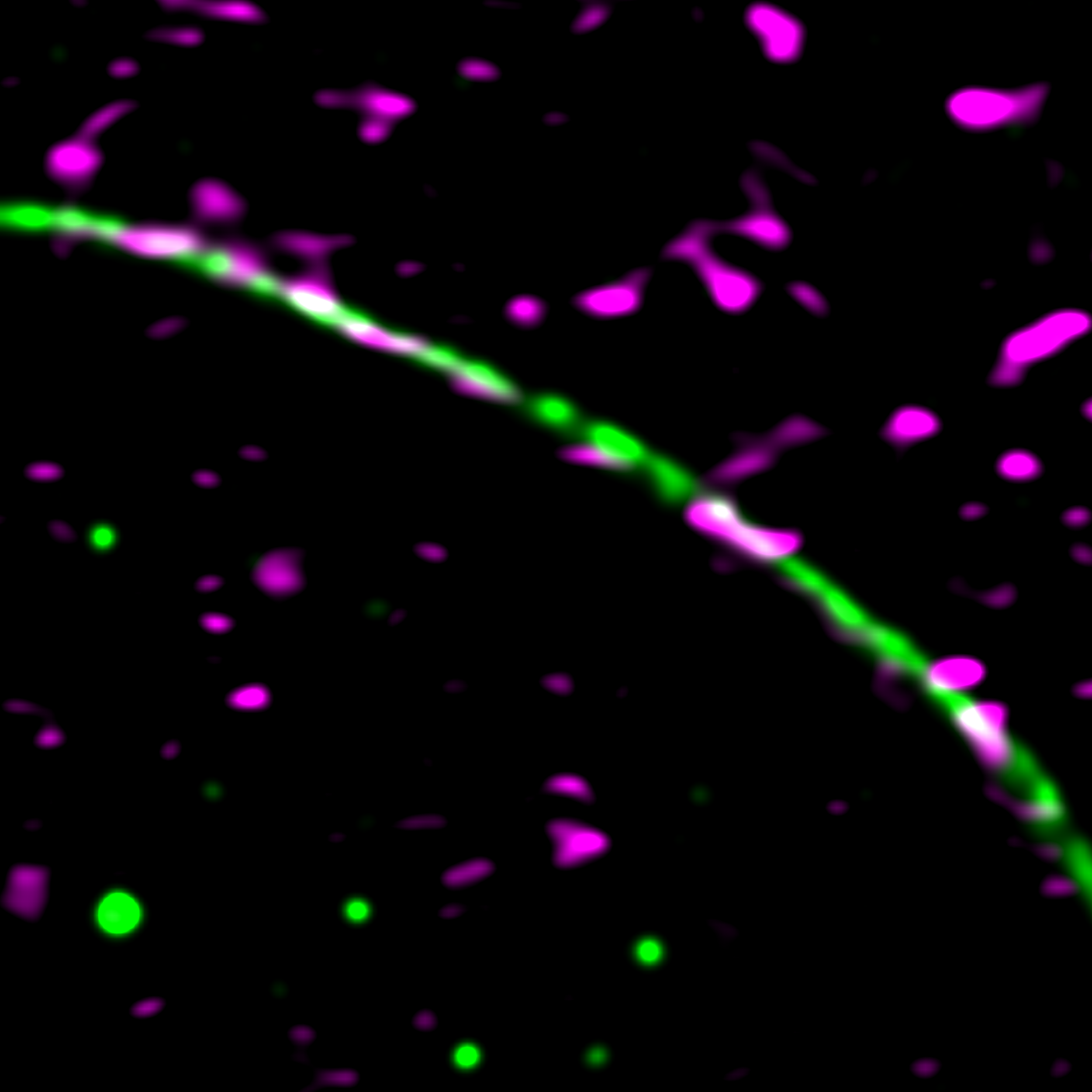

Supplement: Supplementary file 6 — Source data Fig. 2 [file 44319_2024_274_MOESM6_ESM.zip › Figure 2/2B/merge_zoom.tif]

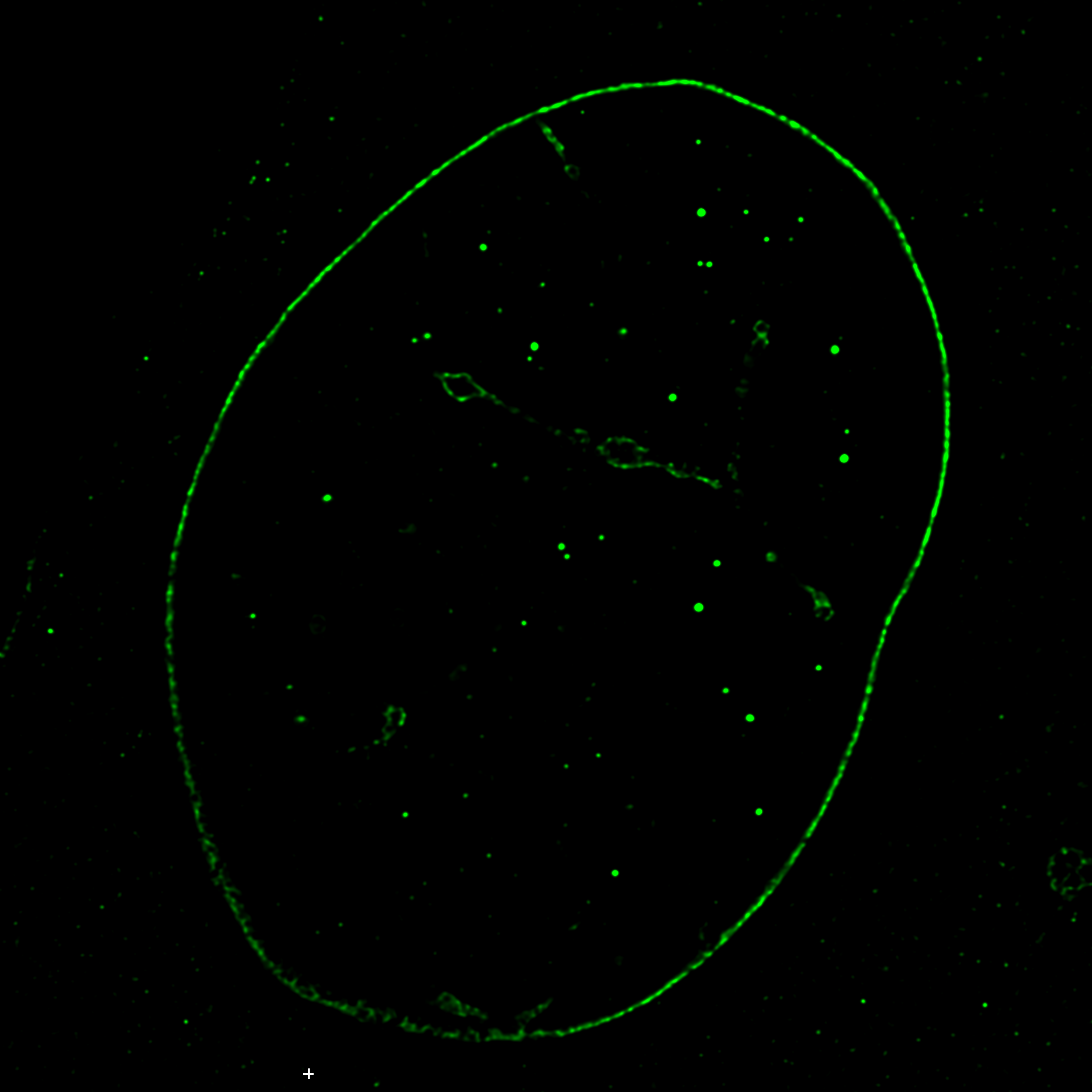

Supplement: Supplementary file 6 — Source data Fig. 2 [file 44319_2024_274_MOESM6_ESM.zip › Figure 2/2B/SUN2-488.tif]

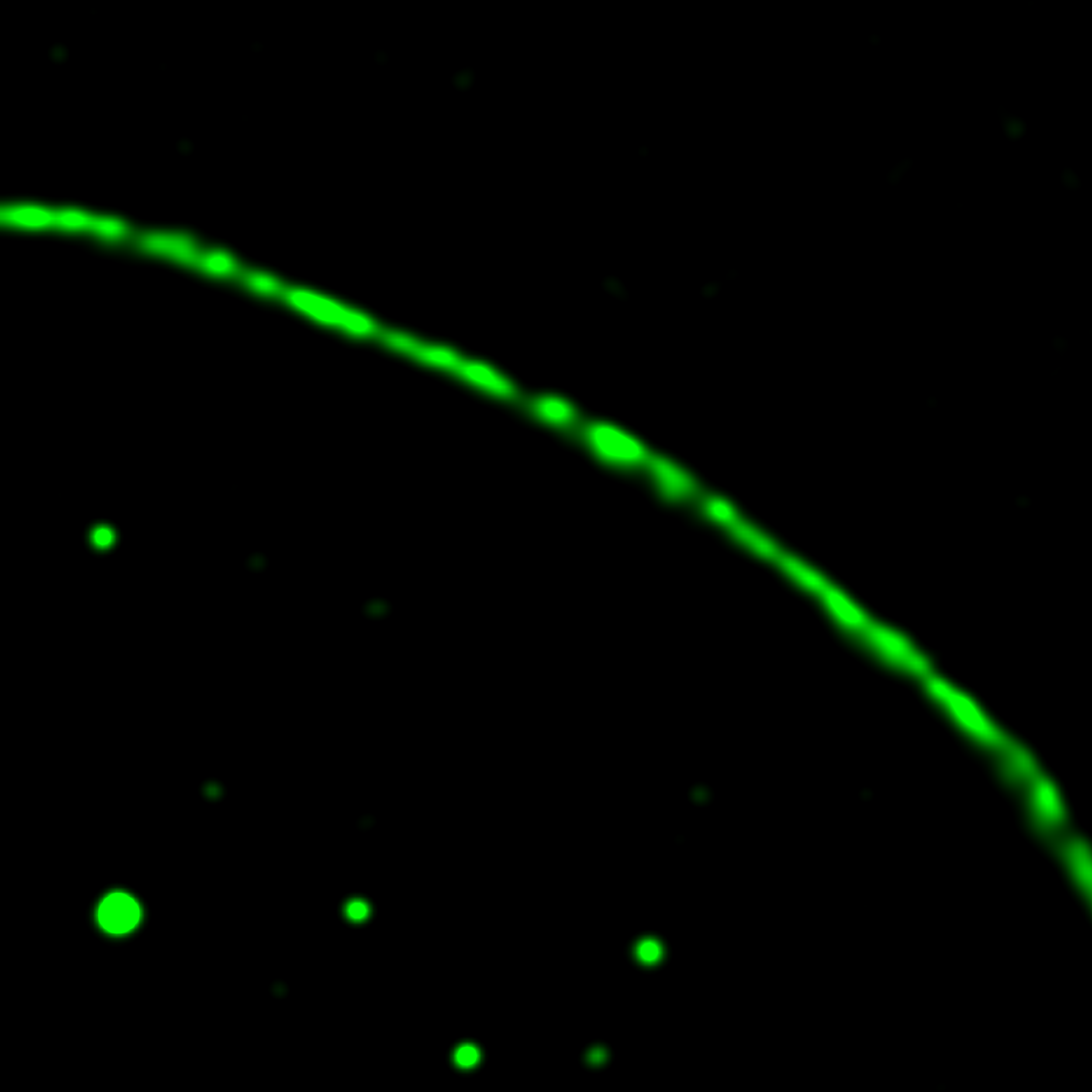

Supplement: Supplementary file 6 — Source data Fig. 2 [file 44319_2024_274_MOESM6_ESM.zip › Figure 2/2B/SUN2-488_zoom.tif]

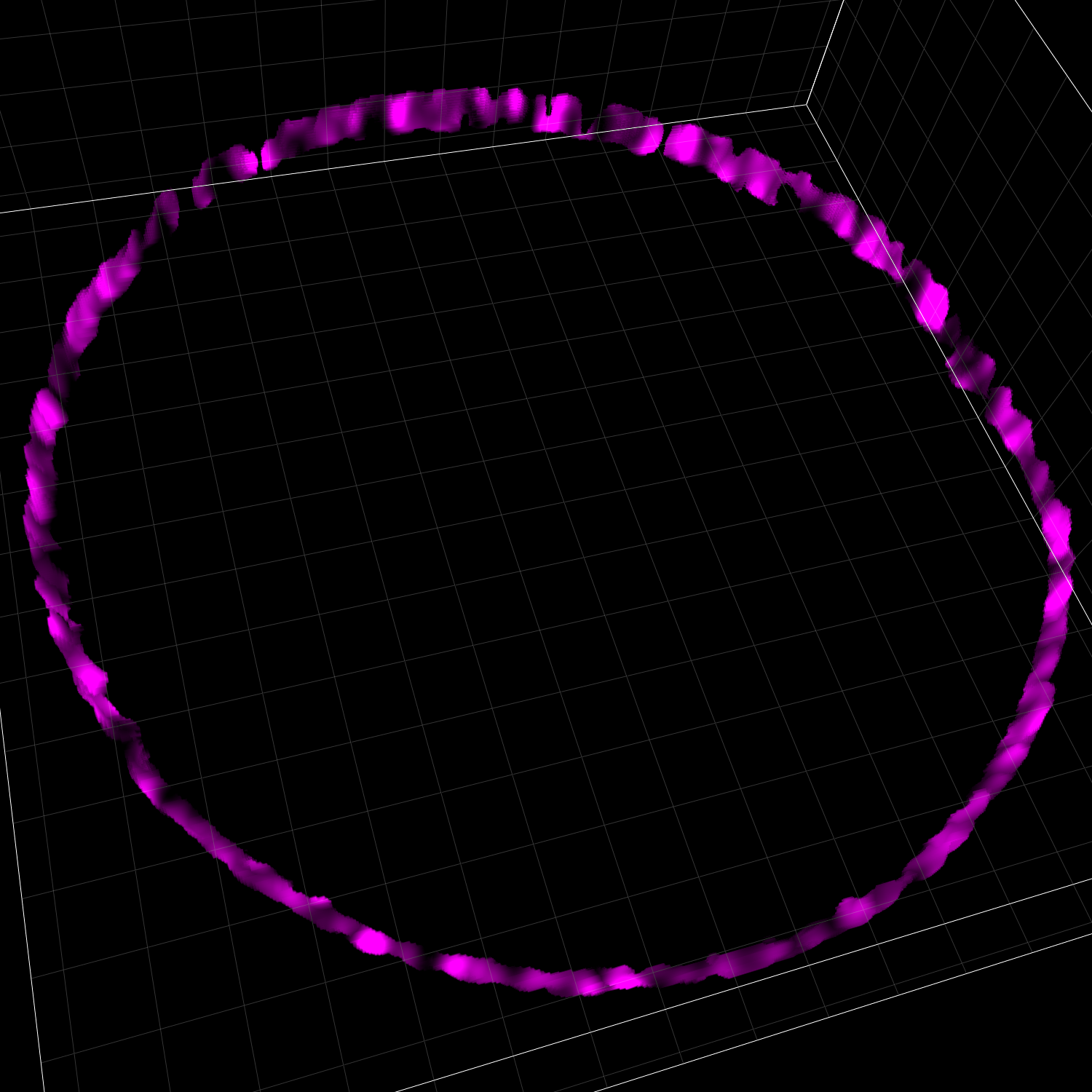

Supplement: Supplementary file 6 — Source data Fig. 2 [file 44319_2024_274_MOESM6_ESM.zip › Figure 2/2C/INF2 within SUN2 mask.tif]

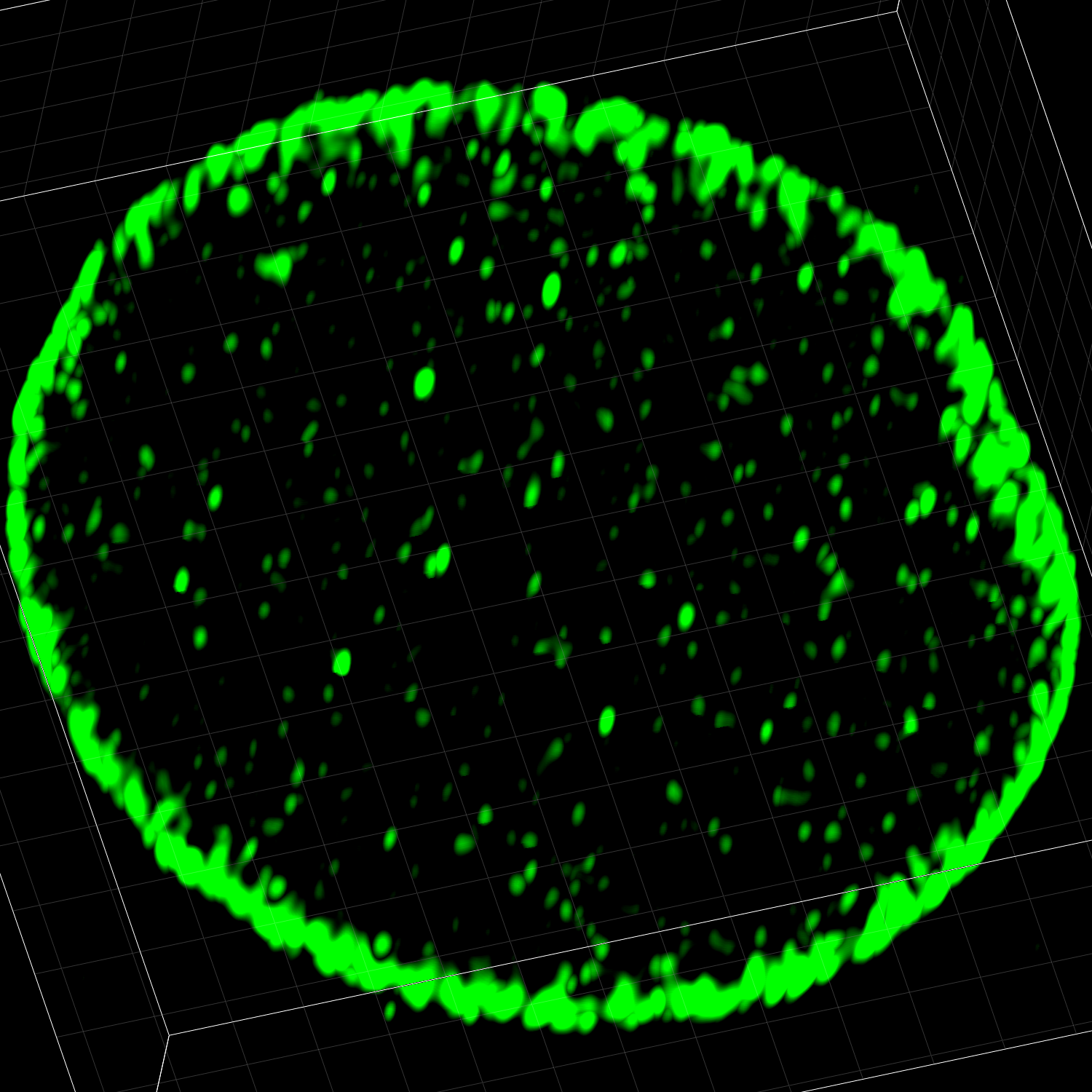

Supplement: Supplementary file 6 — Source data Fig. 2 [file 44319_2024_274_MOESM6_ESM.zip › Figure 2/2C/SUN2.tif]

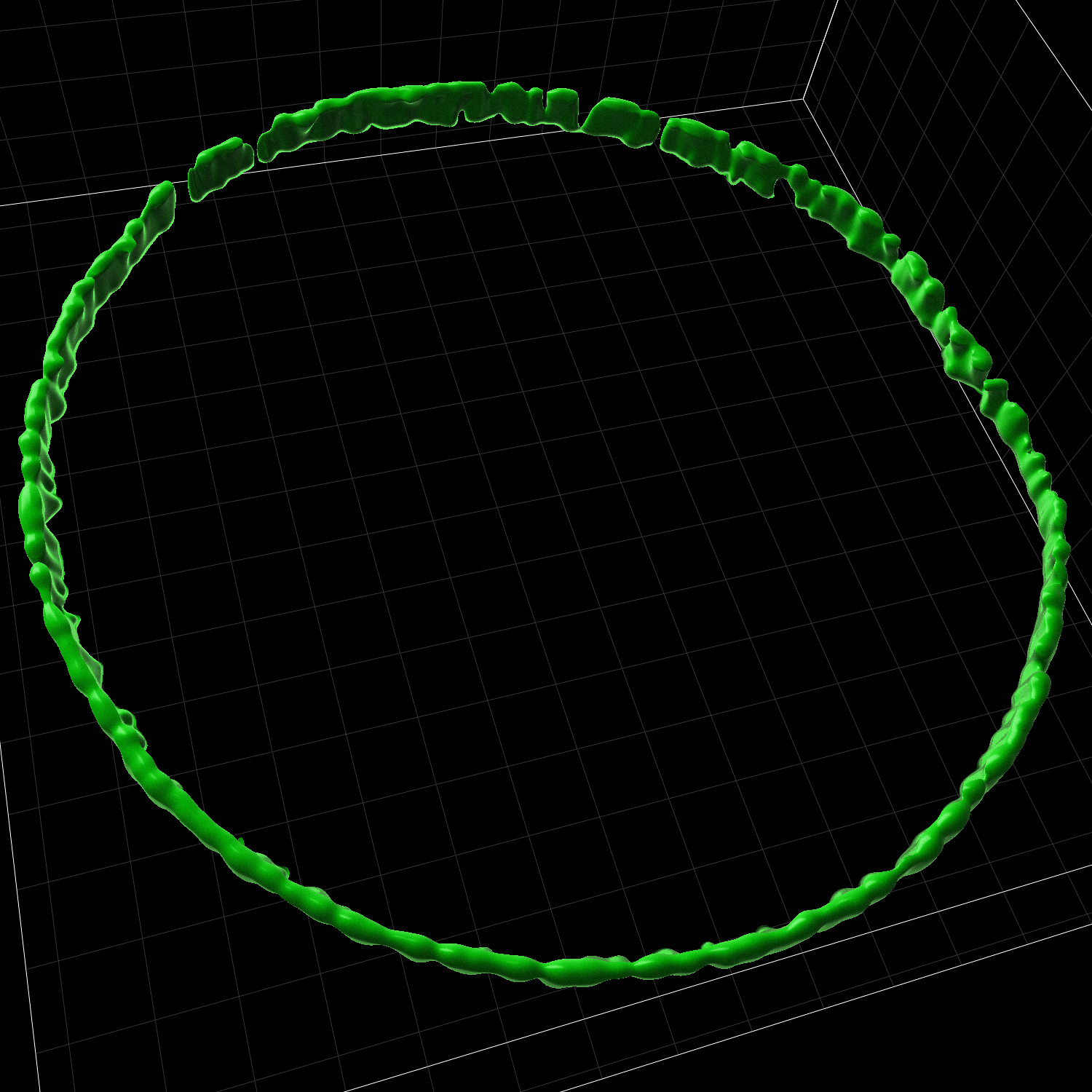

Supplement: Supplementary file 6 — Source data Fig. 2 [file 44319_2024_274_MOESM6_ESM.zip › Figure 2/2C/SUN2_mask.tif]

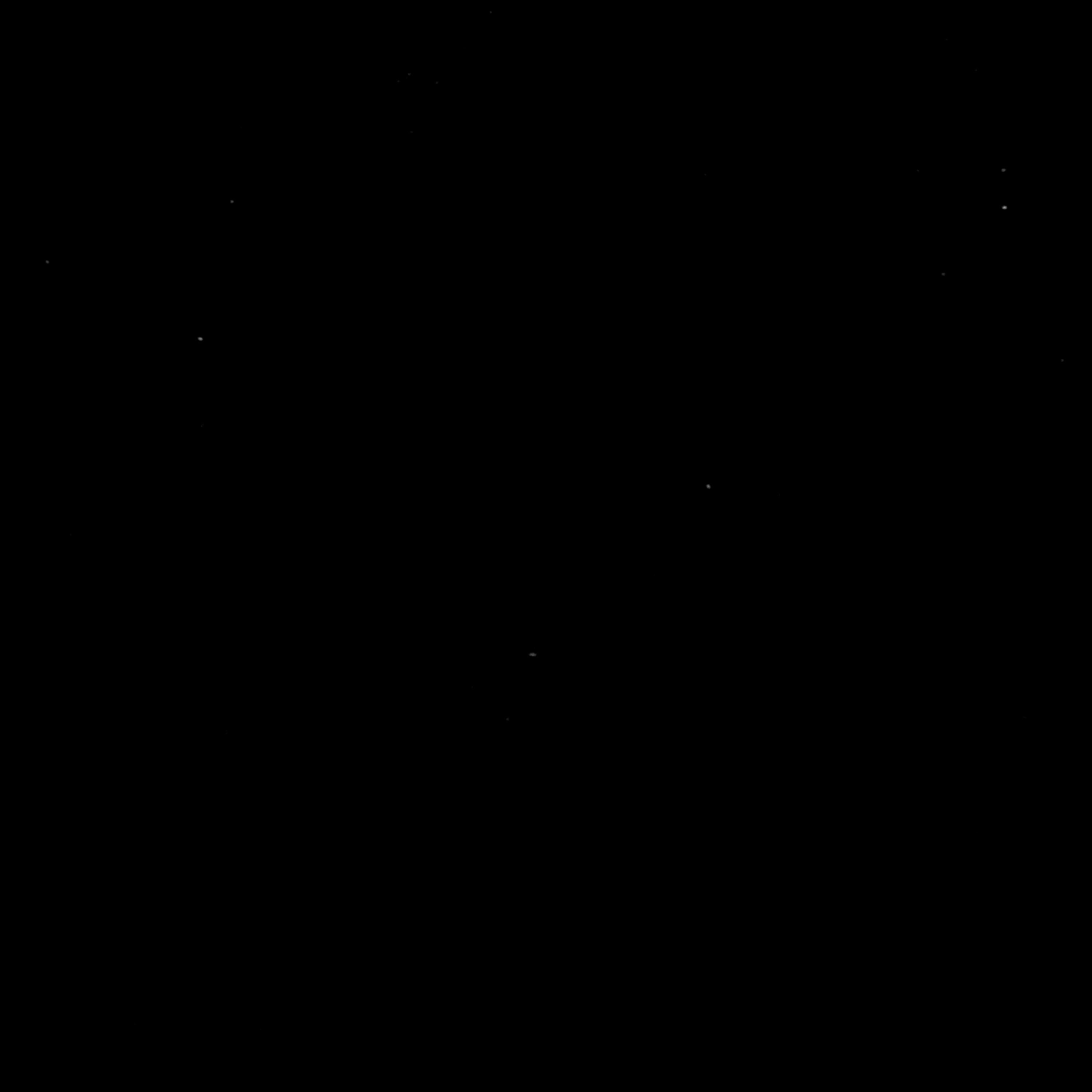

Supplement: Supplementary file 7 — Source data Fig. 3 [file 44319_2024_274_MOESM7_ESM.zip › Figure 3/3A/INF2 only_Cy5.tif]

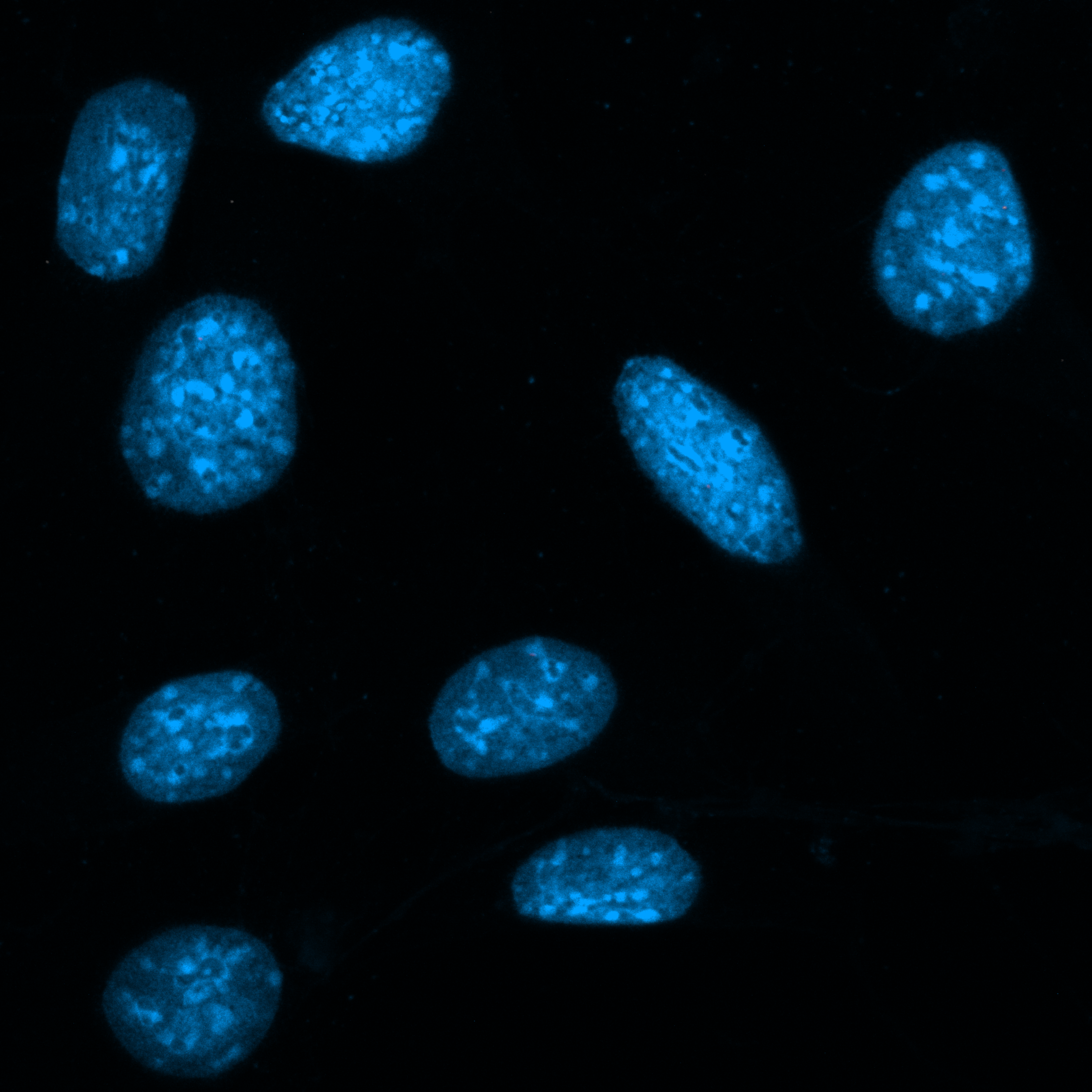

Supplement: Supplementary file 7 — Source data Fig. 3 [file 44319_2024_274_MOESM7_ESM.zip › Figure 3/3A/INF2 only_merge.tif]

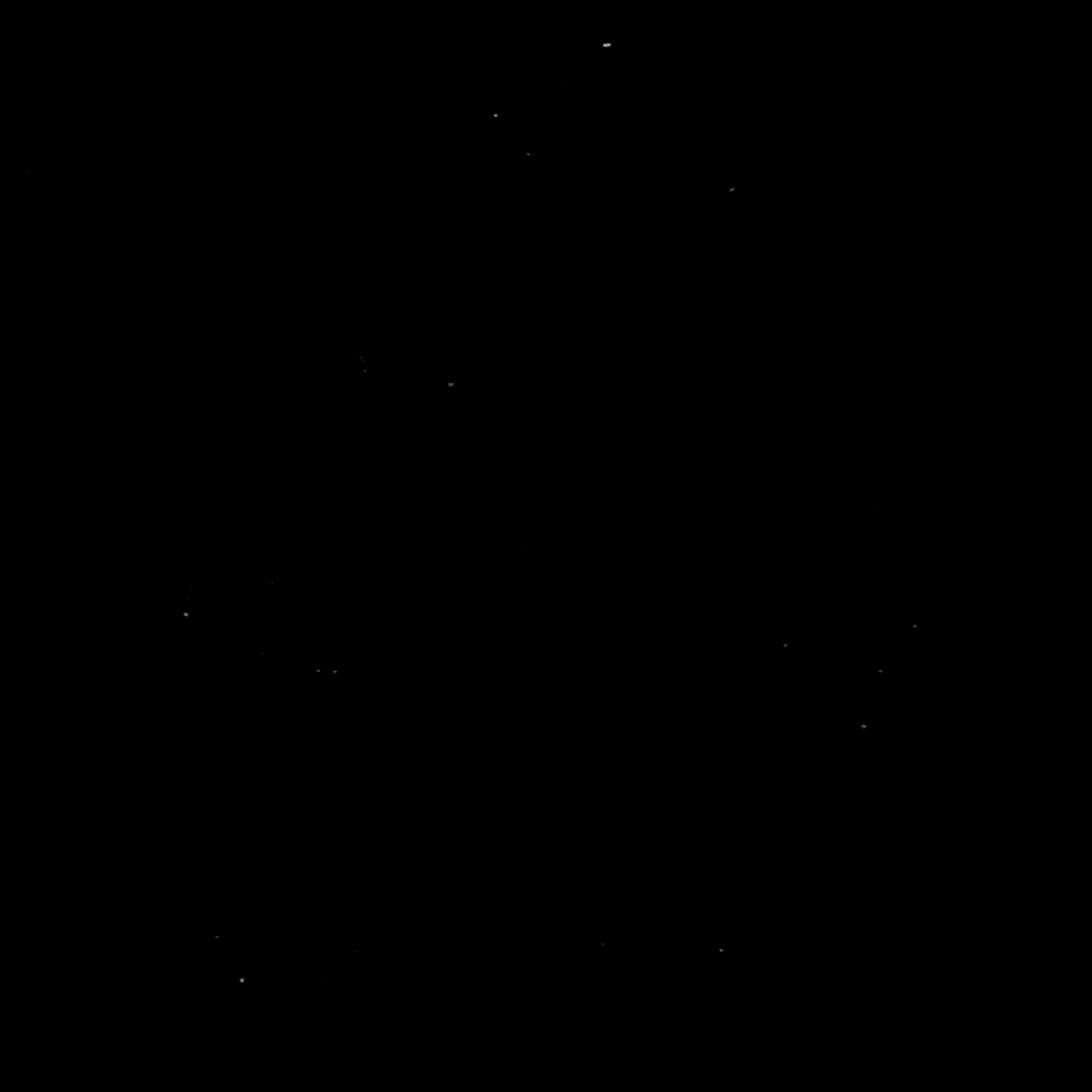

Supplement: Supplementary file 7 — Source data Fig. 3 [file 44319_2024_274_MOESM7_ESM.zip › Figure 3/3A/SUN2 only_Cy5.tif]

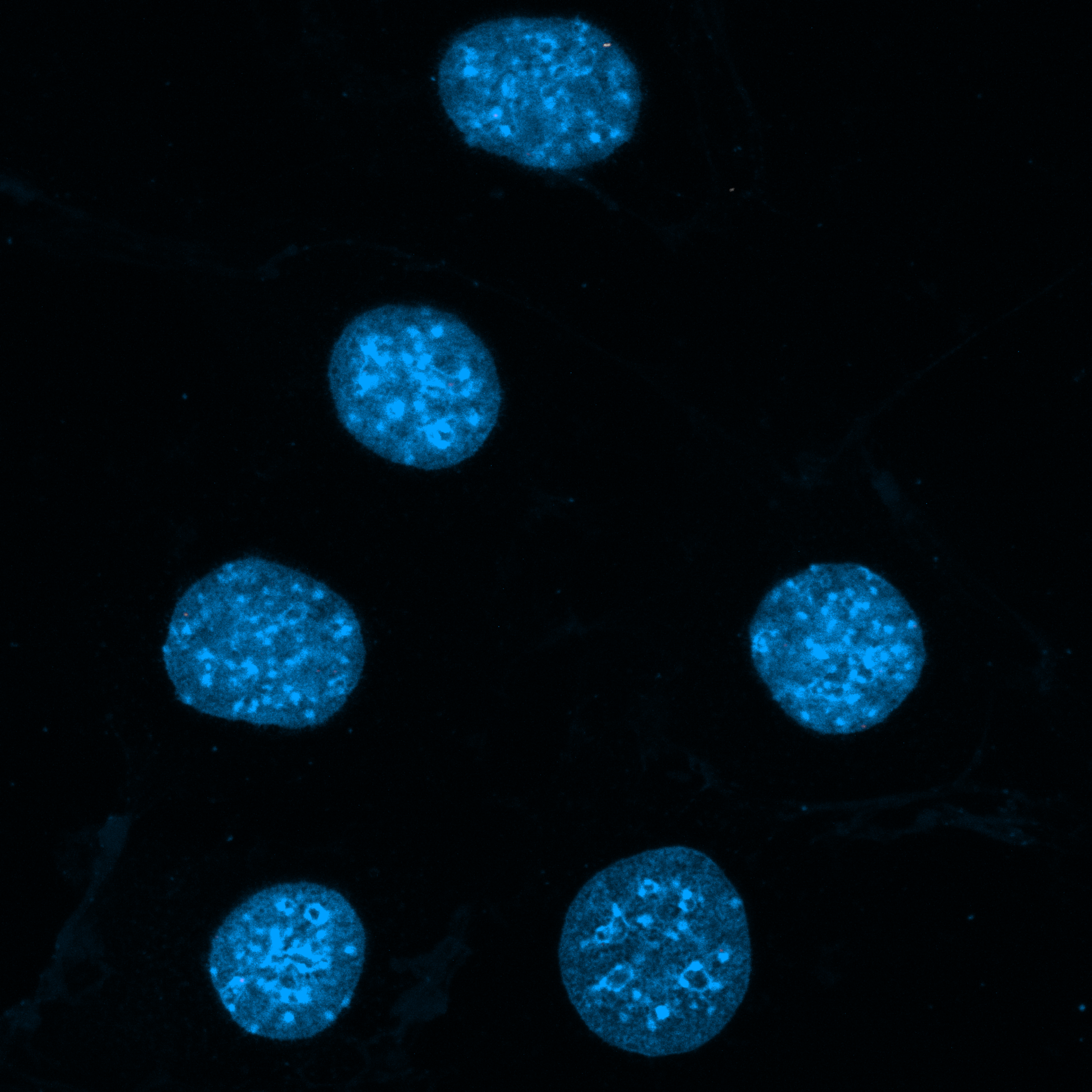

Supplement: Supplementary file 7 — Source data Fig. 3 [file 44319_2024_274_MOESM7_ESM.zip › Figure 3/3A/SUN2 only_merge.tif]

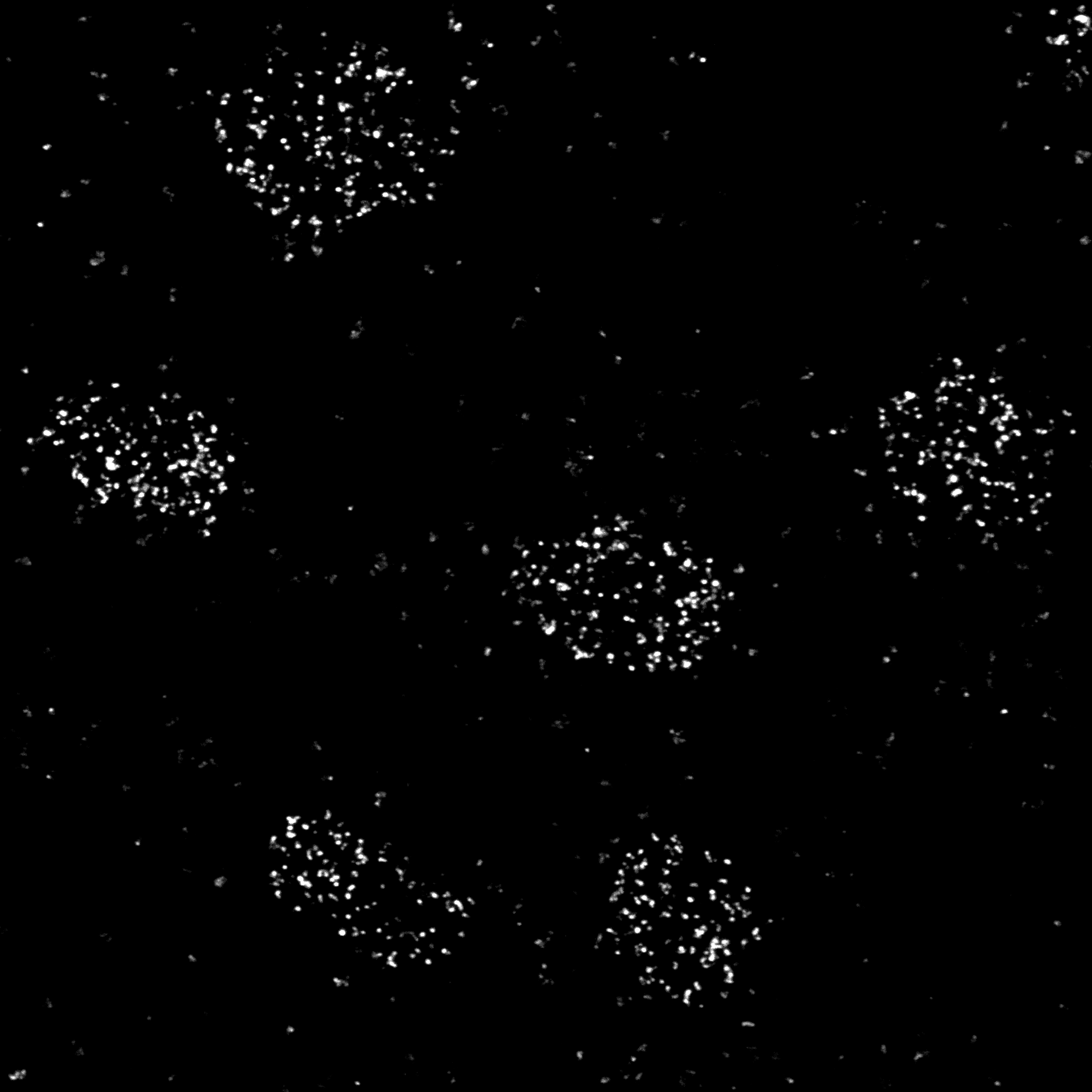

Supplement: Supplementary file 7 — Source data Fig. 3 [file 44319_2024_274_MOESM7_ESM.zip › Figure 3/3A/SUN2+ INF2 +A23187_Cy5.tif]

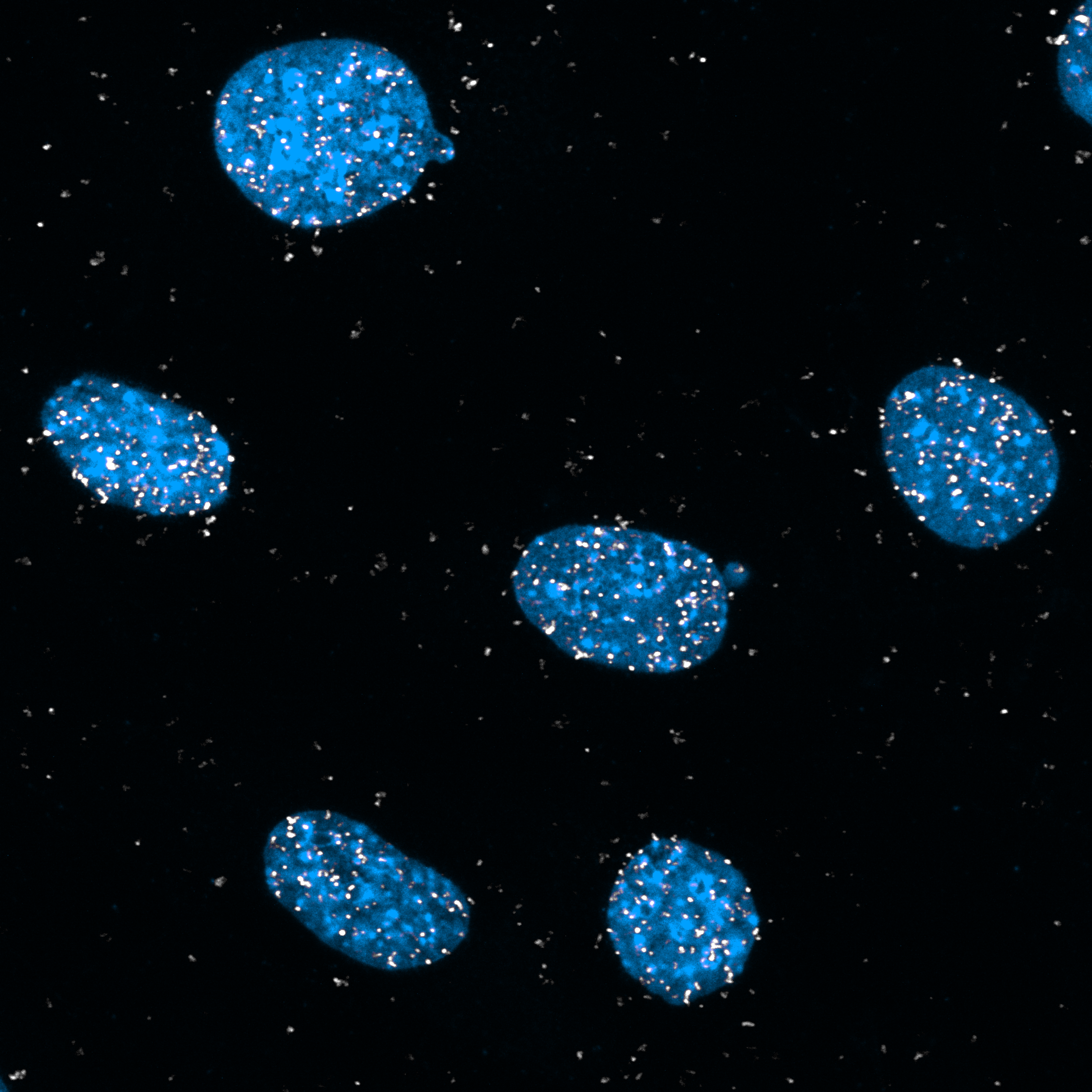

Supplement: Supplementary file 7 — Source data Fig. 3 [file 44319_2024_274_MOESM7_ESM.zip › Figure 3/3A/SUN2+ INF2 +A23187_merge.tif]

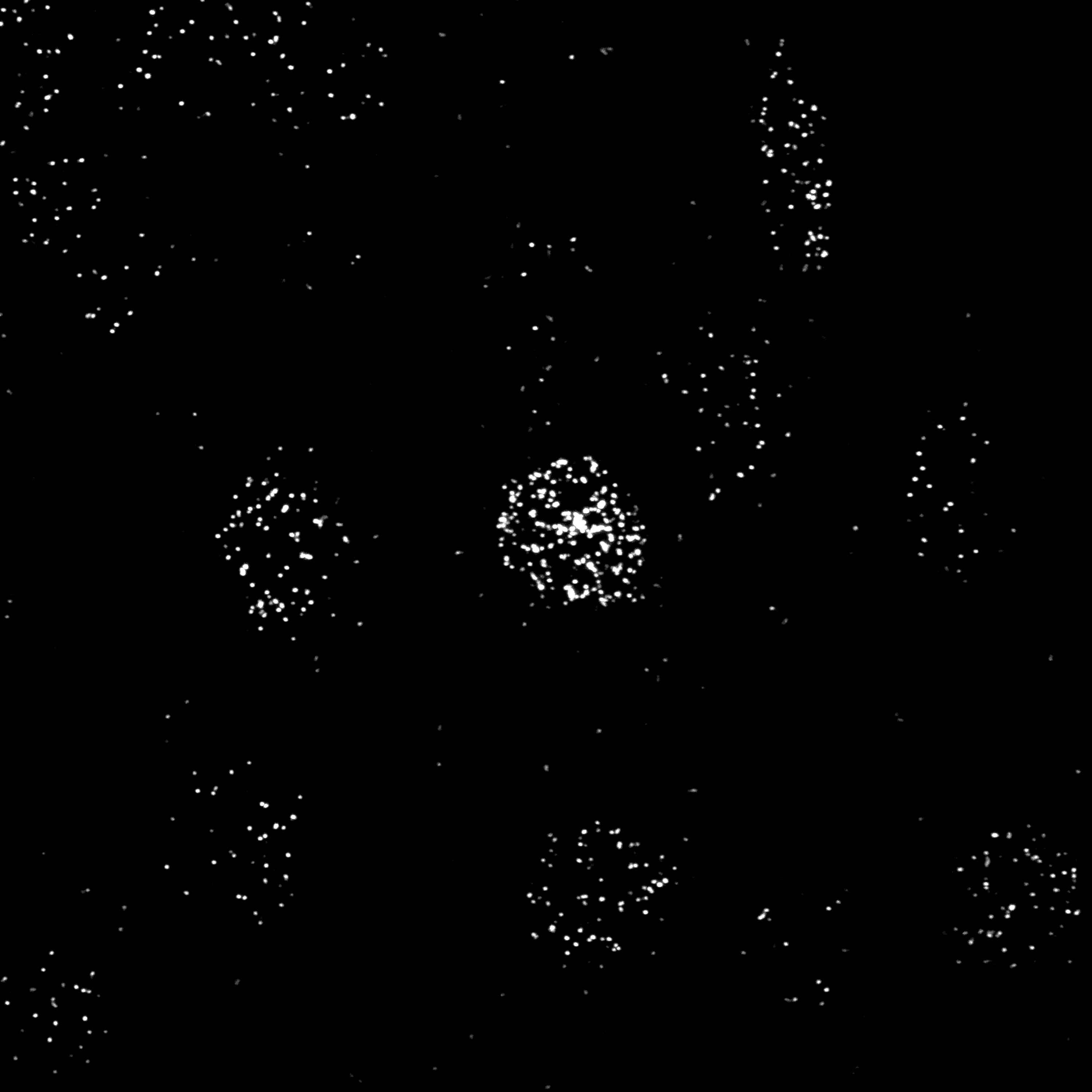

Supplement: Supplementary file 7 — Source data Fig. 3 [file 44319_2024_274_MOESM7_ESM.zip › Figure 3/3A/SUN2+INF2_Cy5.tif]

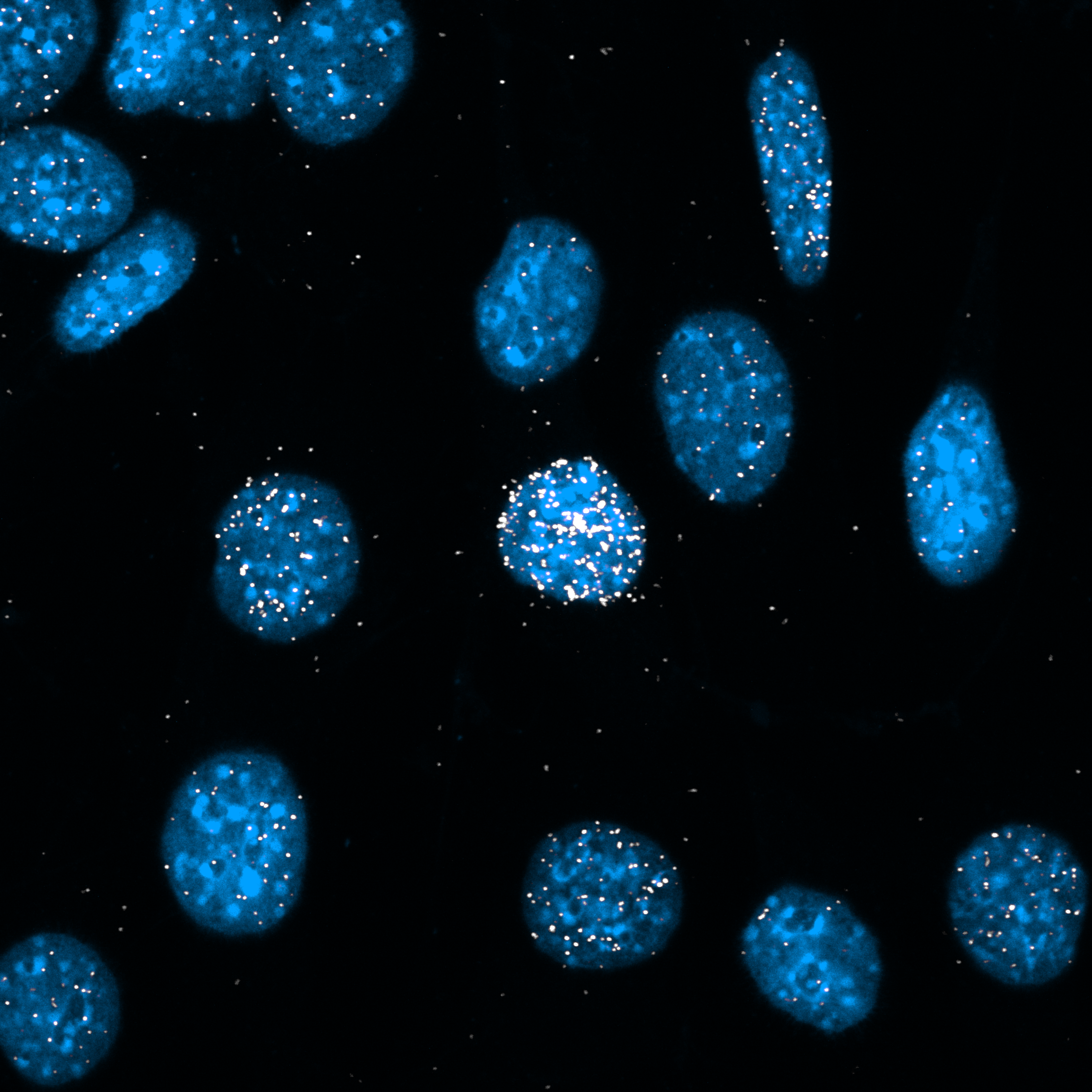

Supplement: Supplementary file 7 — Source data Fig. 3 [file 44319_2024_274_MOESM7_ESM.zip › Figure 3/3A/SUN2+INF2_merge.tif]

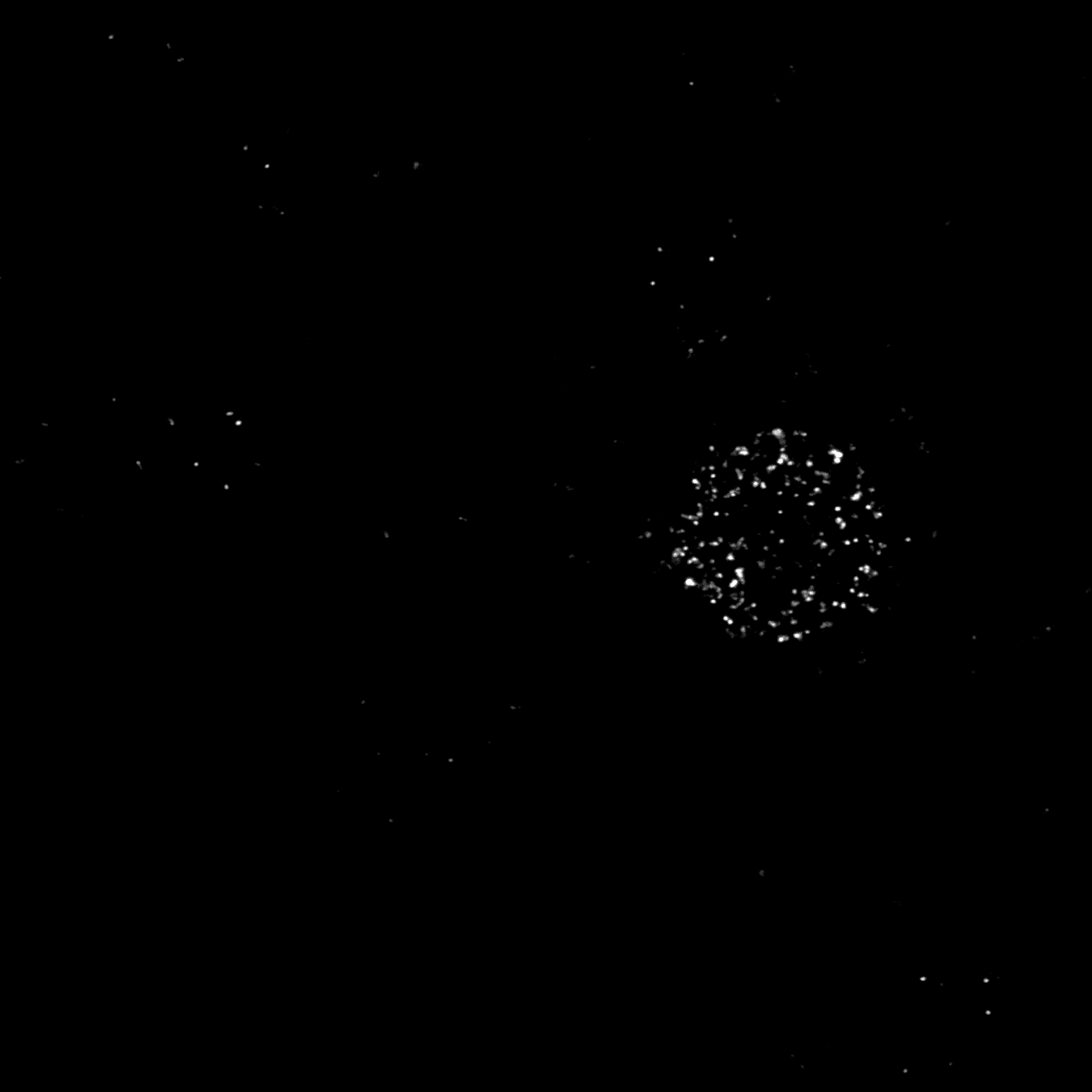

Supplement: Supplementary file 7 — Source data Fig. 3 [file 44319_2024_274_MOESM7_ESM.zip › Figure 3/3A/SUN2+INF2_siSUN2_Cy5.tif]

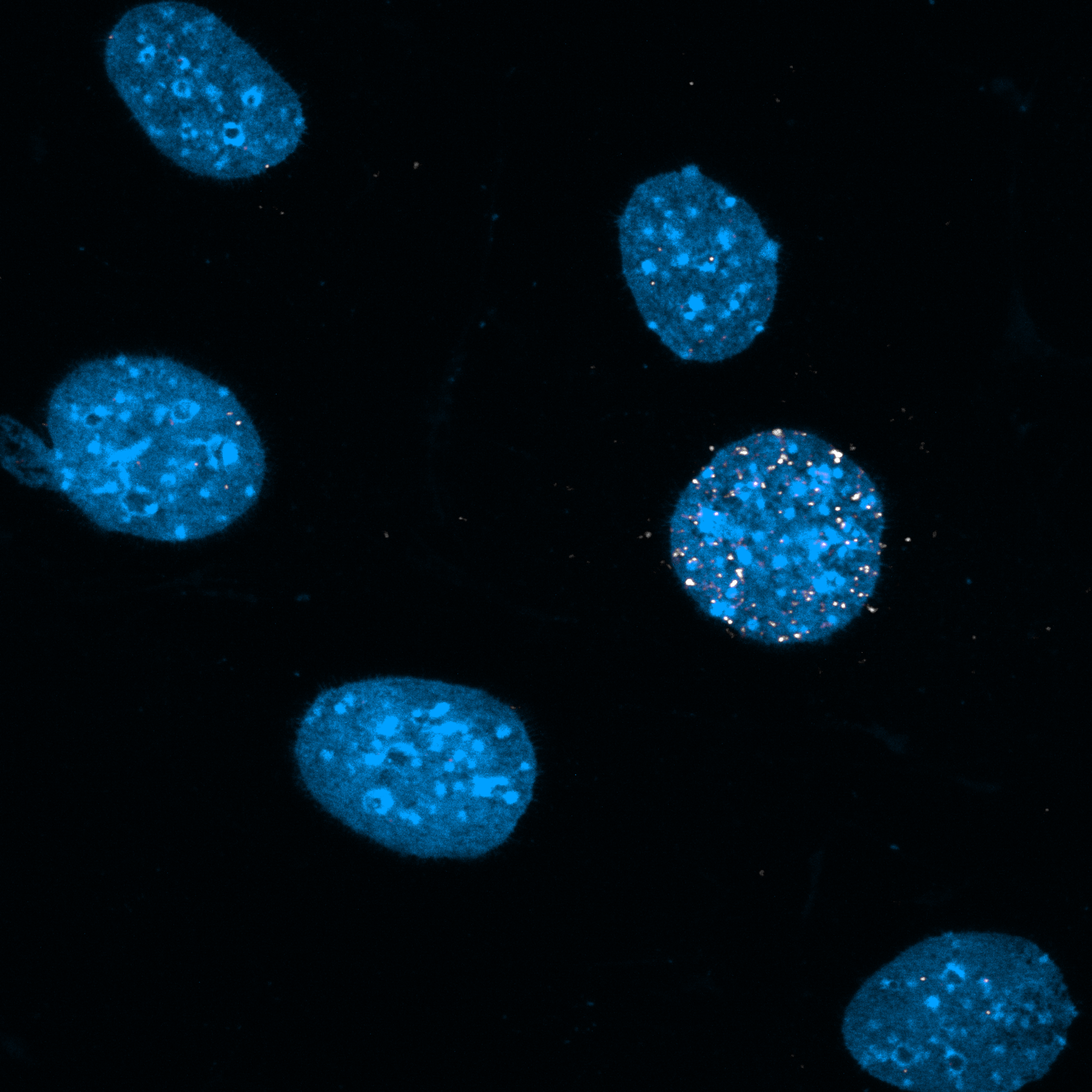

Supplement: Supplementary file 7 — Source data Fig. 3 [file 44319_2024_274_MOESM7_ESM.zip › Figure 3/3A/SUN2+INF2_siSUN2_merge.tif]

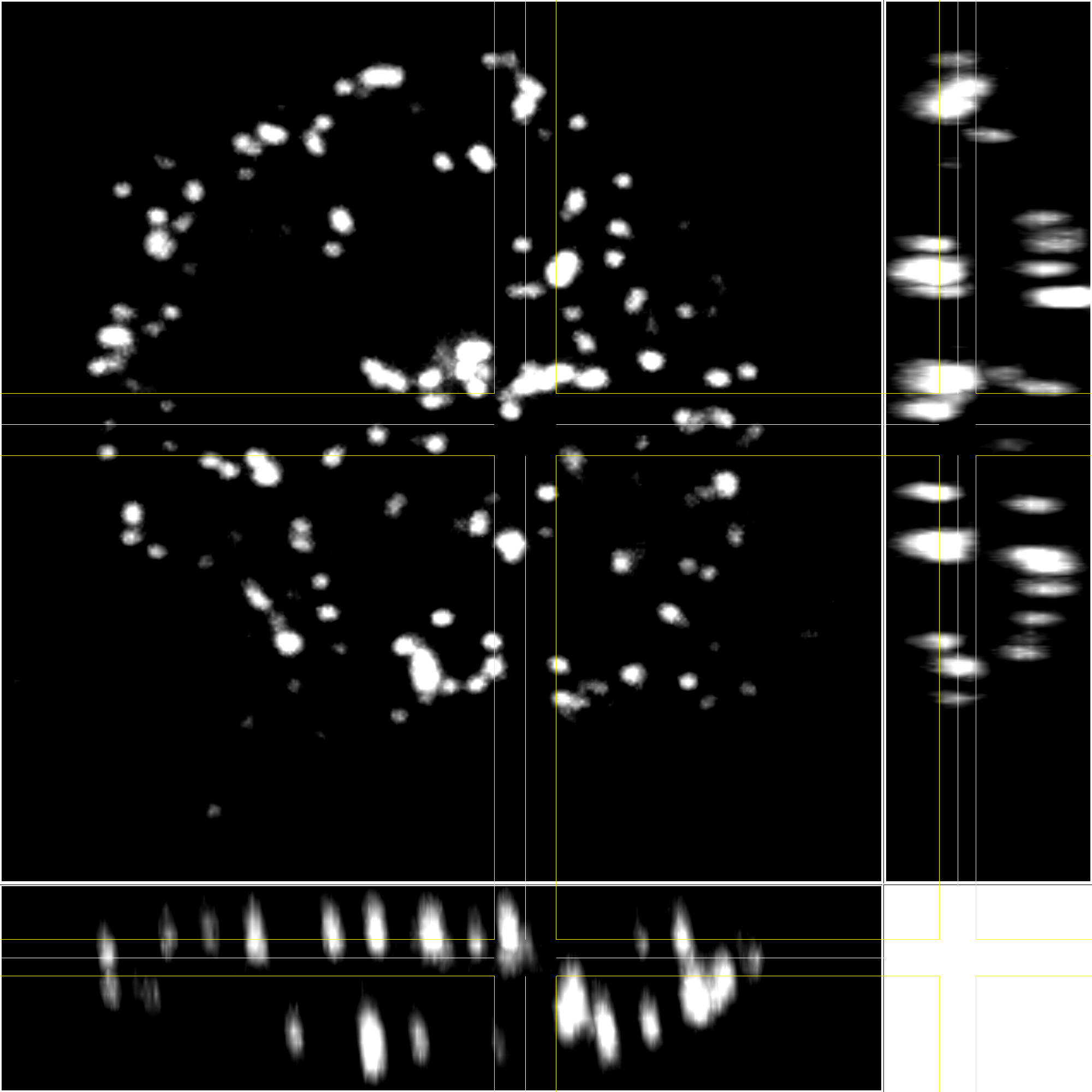

Supplement: Supplementary file 7 — Source data Fig. 3 [file 44319_2024_274_MOESM7_ESM.zip › Figure 3/3C/SUN2+INF2_side view_Cy5.tif]

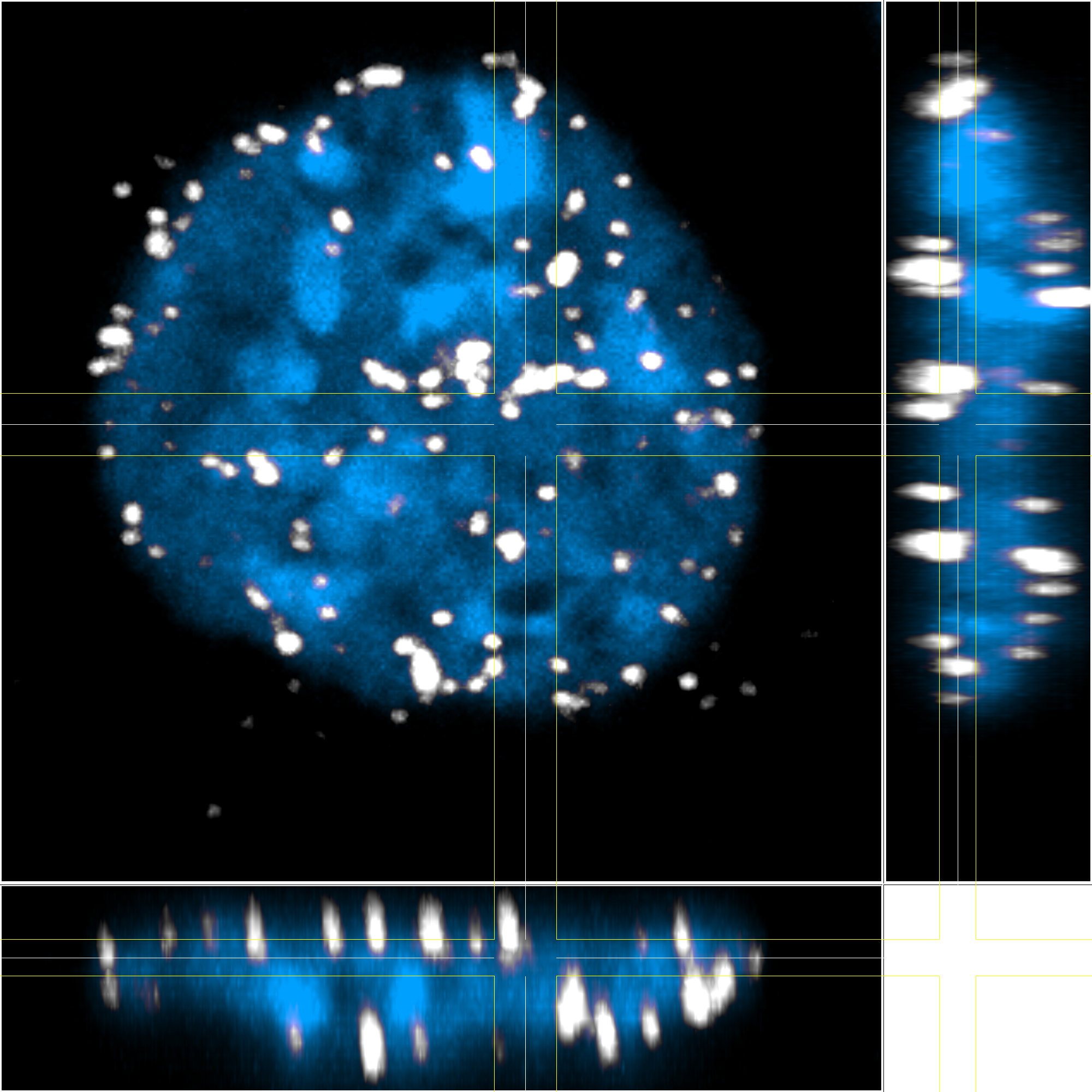

Supplement: Supplementary file 7 — Source data Fig. 3 [file 44319_2024_274_MOESM7_ESM.zip › Figure 3/3C/SUN2+INF2_side view_merge.tif]

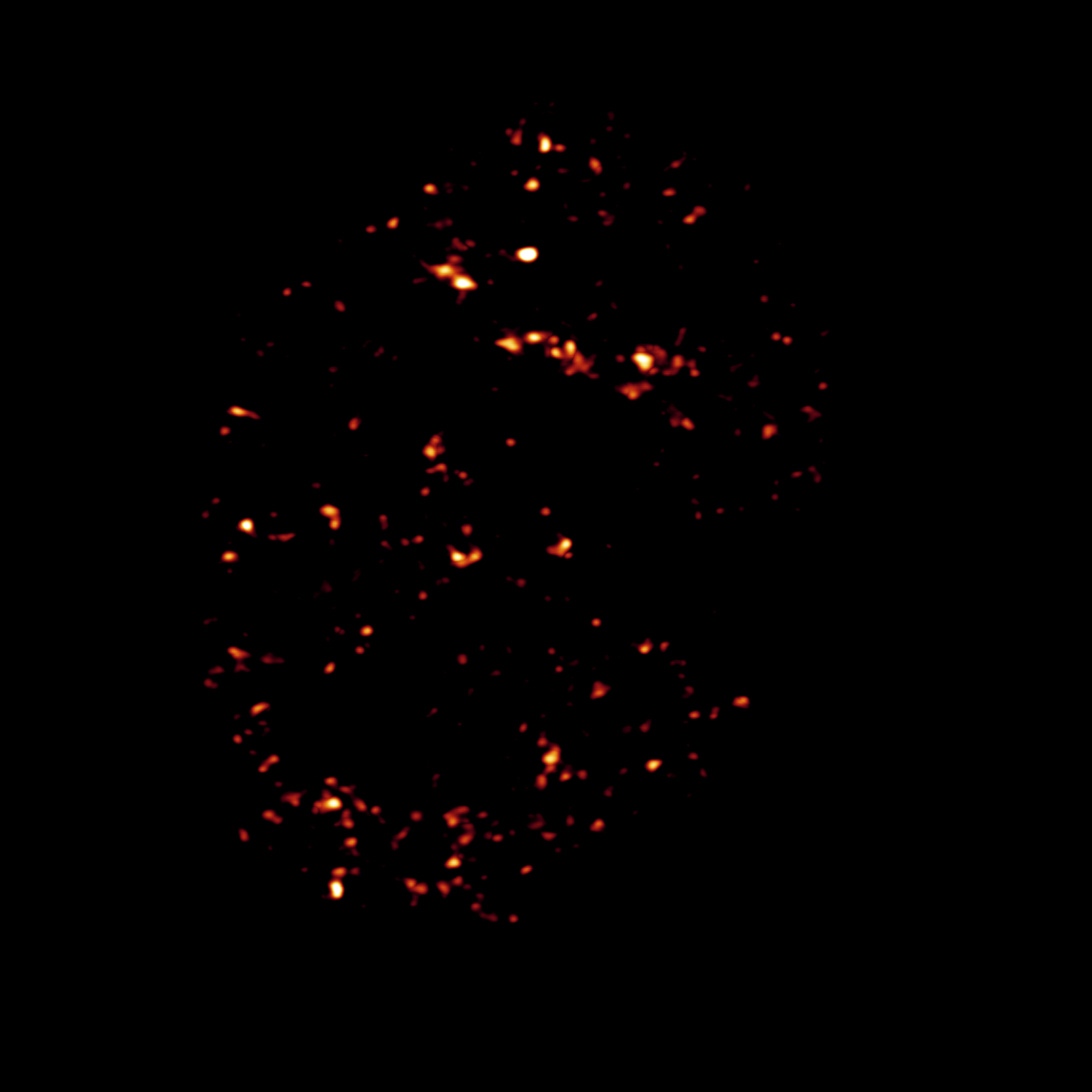

Supplement: Supplementary file 8 — Source data Fig. 4 [file 44319_2024_274_MOESM8_ESM.zip › Figure 4/4A/siCtrl_0 min.tif]

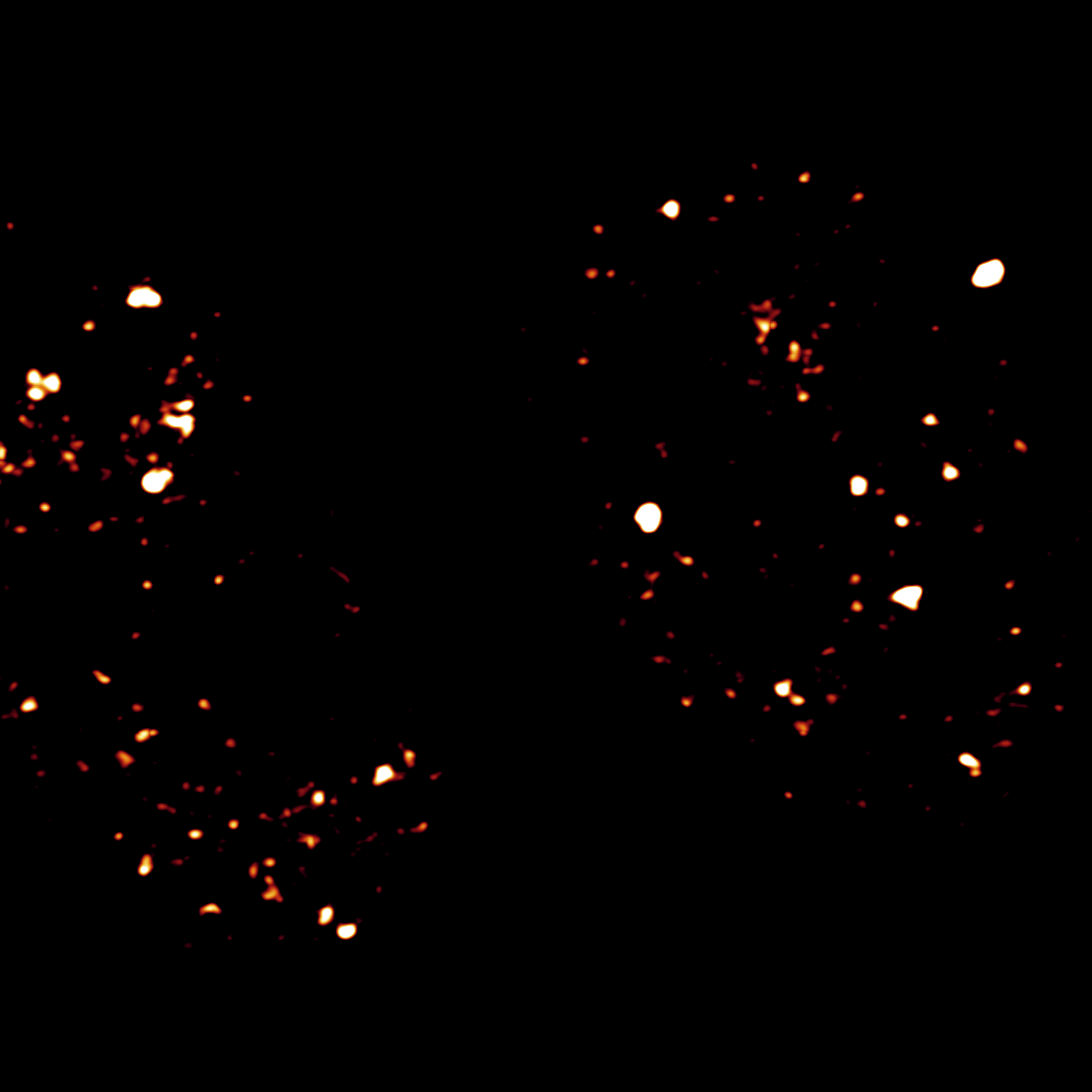

Supplement: Supplementary file 8 — Source data Fig. 4 [file 44319_2024_274_MOESM8_ESM.zip › Figure 4/4A/siCtrl_10 min.tif]

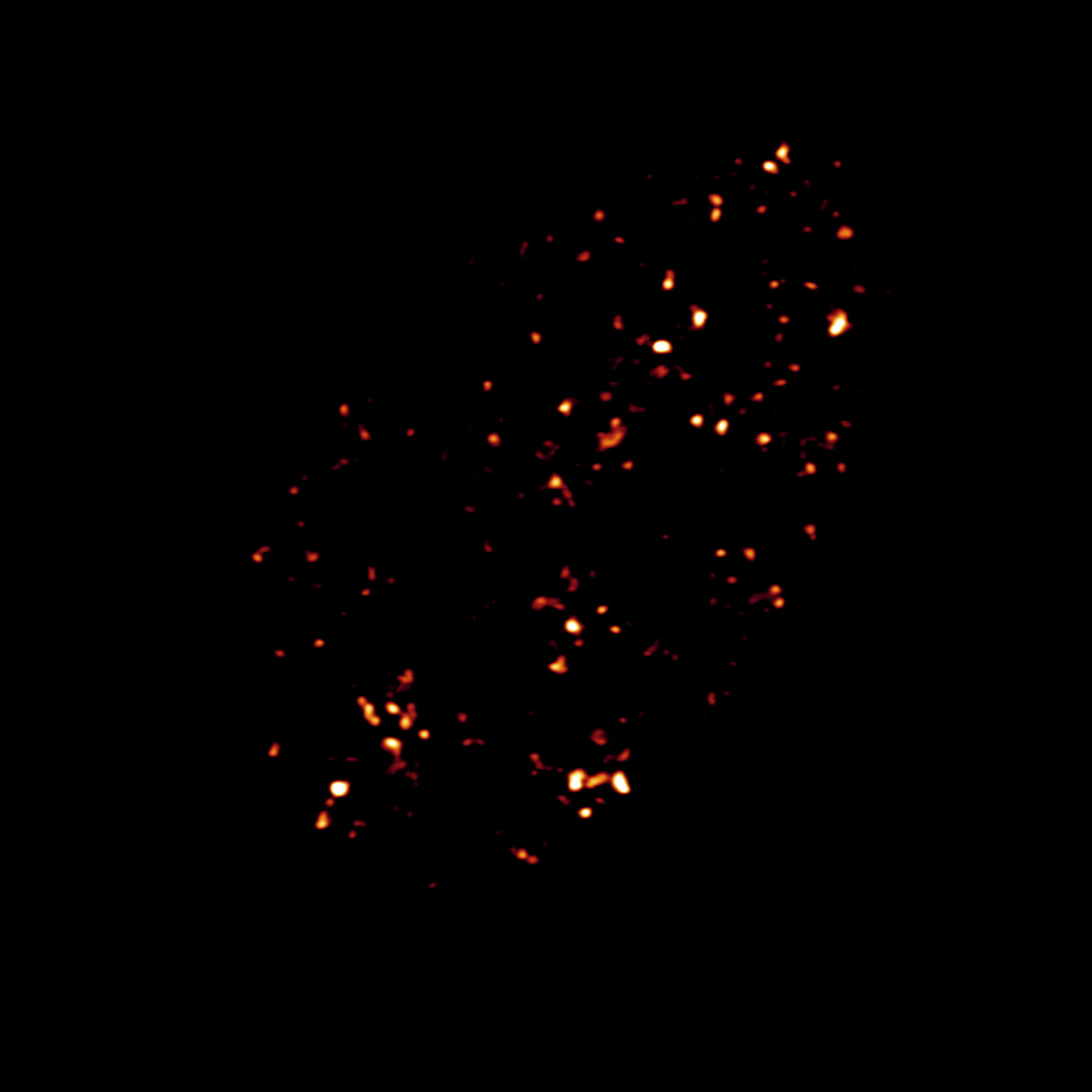

Supplement: Supplementary file 8 — Source data Fig. 4 [file 44319_2024_274_MOESM8_ESM.zip › Figure 4/4A/siCtrl_120min.tif]

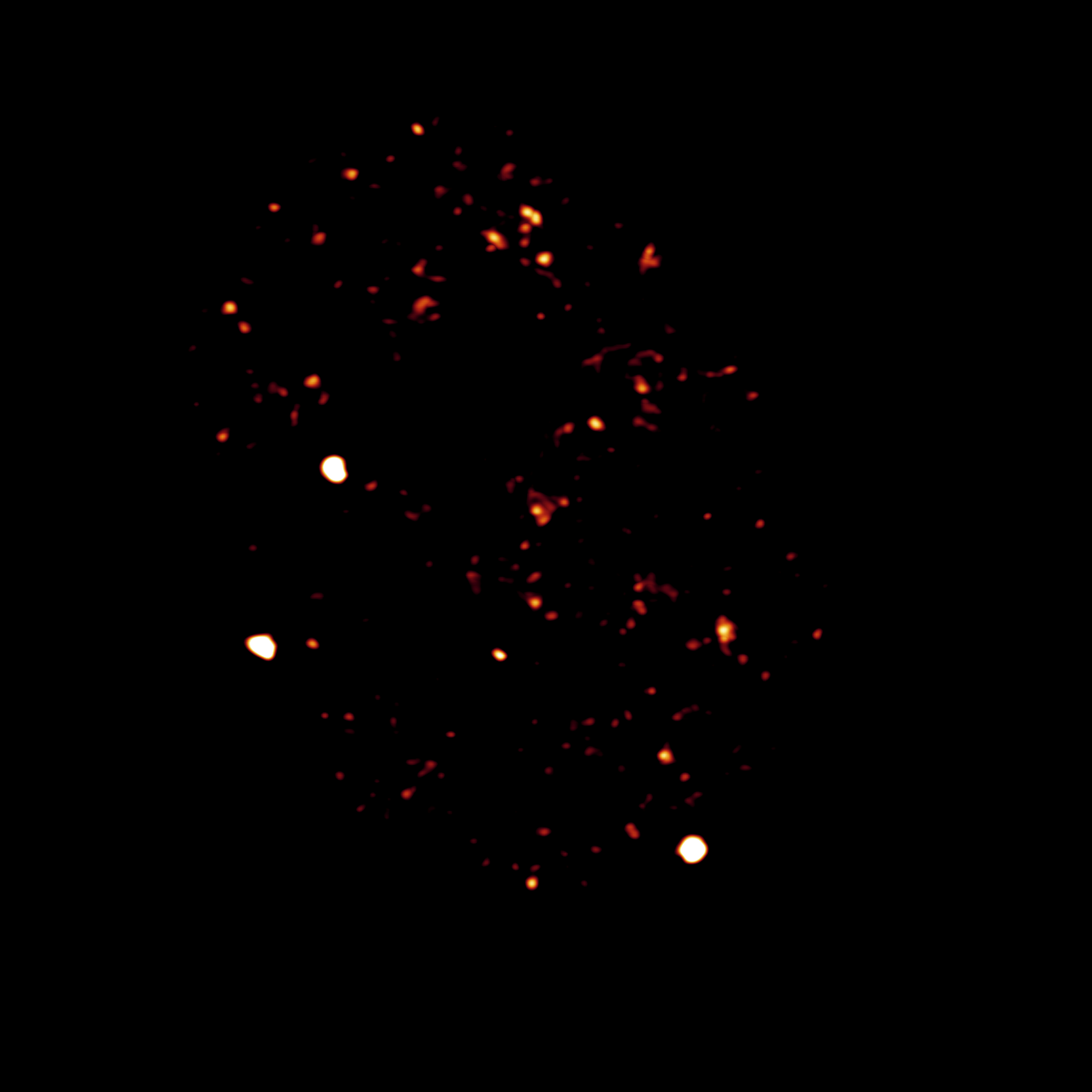

Supplement: Supplementary file 8 — Source data Fig. 4 [file 44319_2024_274_MOESM8_ESM.zip › Figure 4/4A/siCtrl_30 min.tif]

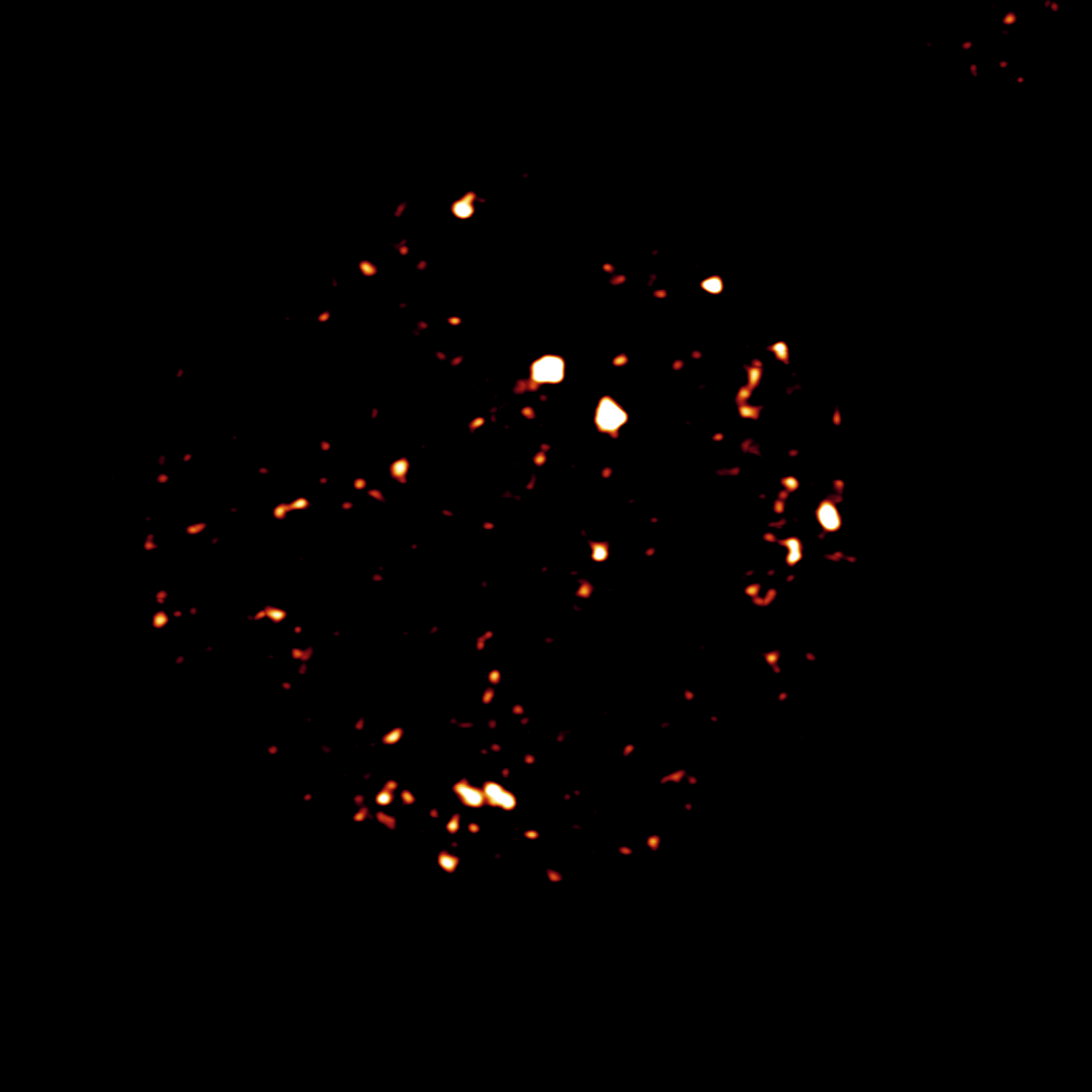

Supplement: Supplementary file 8 — Source data Fig. 4 [file 44319_2024_274_MOESM8_ESM.zip › Figure 4/4A/siCtrl_5 min.tif]

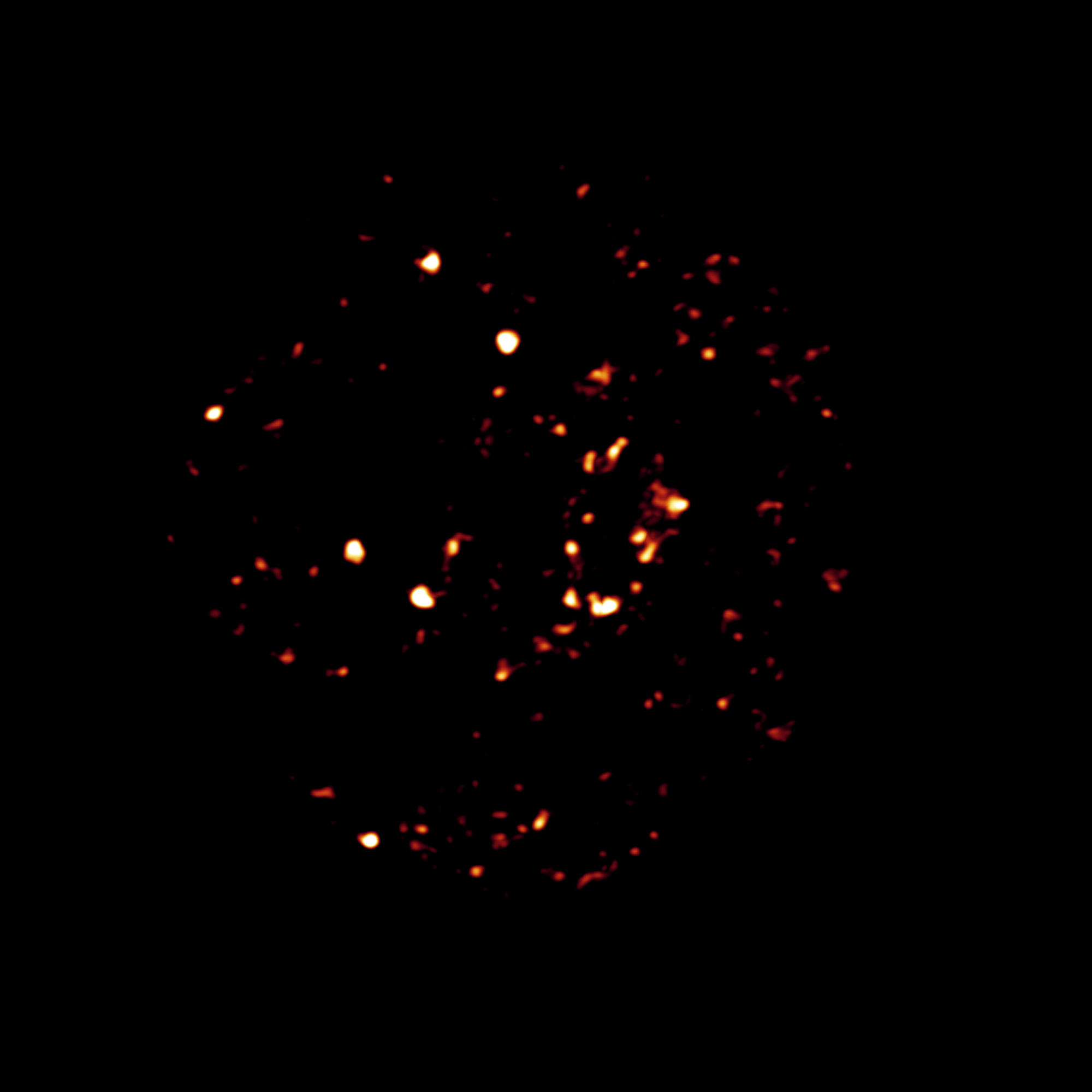

Supplement: Supplementary file 8 — Source data Fig. 4 [file 44319_2024_274_MOESM8_ESM.zip › Figure 4/4B/siSUN2_0 min.tif]

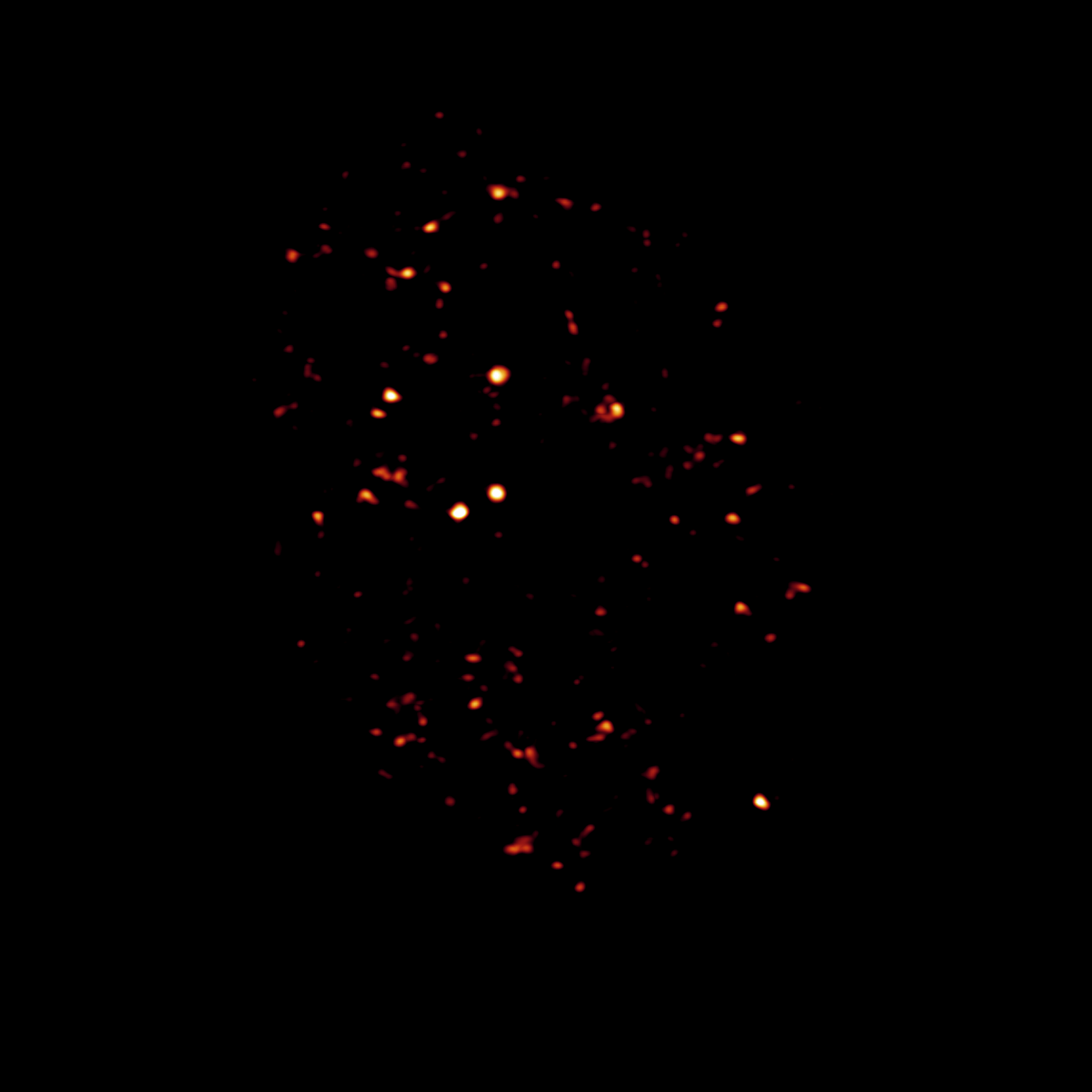

Supplement: Supplementary file 8 — Source data Fig. 4 [file 44319_2024_274_MOESM8_ESM.zip › Figure 4/4B/siSUN2_10 min.tif]

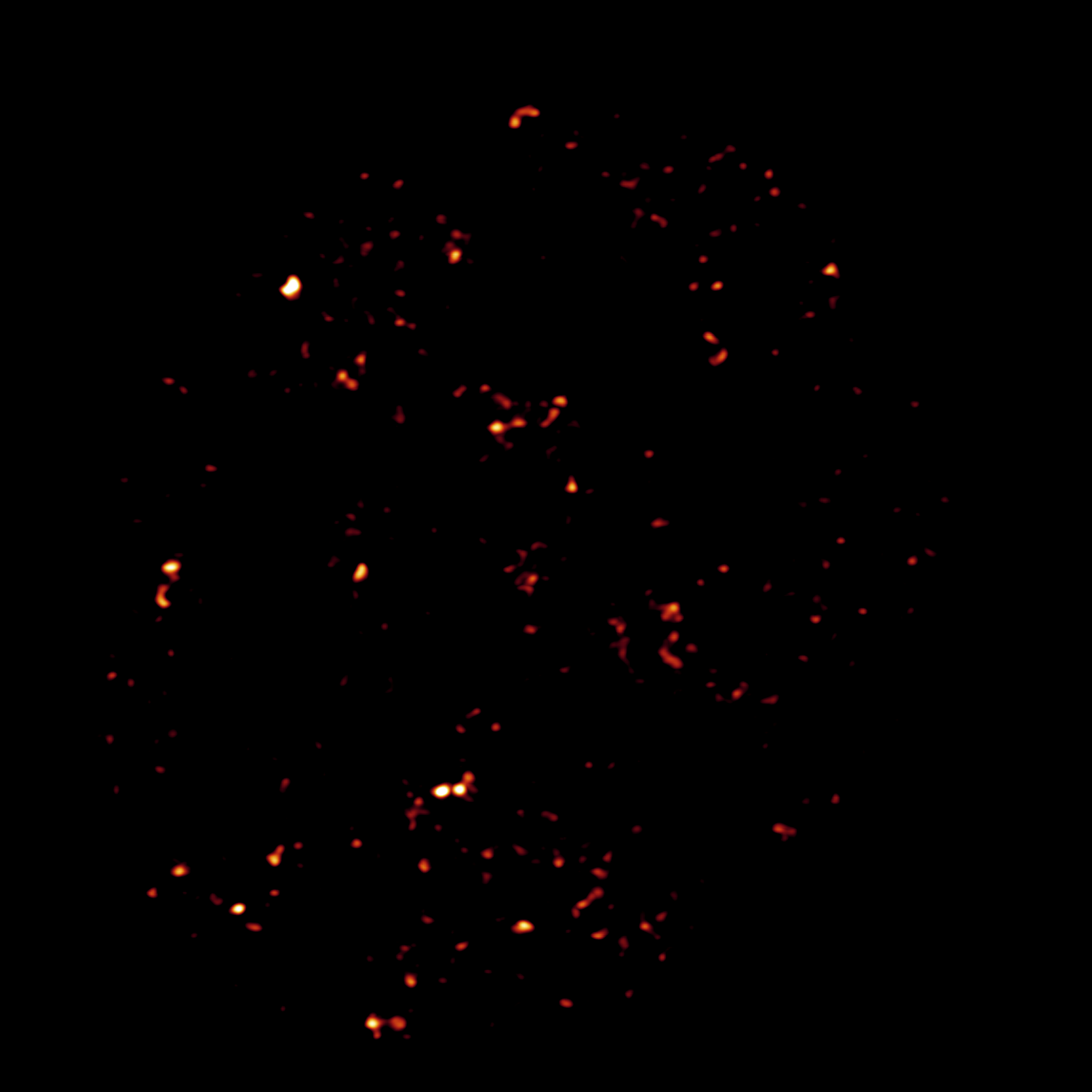

Supplement: Supplementary file 8 — Source data Fig. 4 [file 44319_2024_274_MOESM8_ESM.zip › Figure 4/4B/siSUN2_120 min.tif]

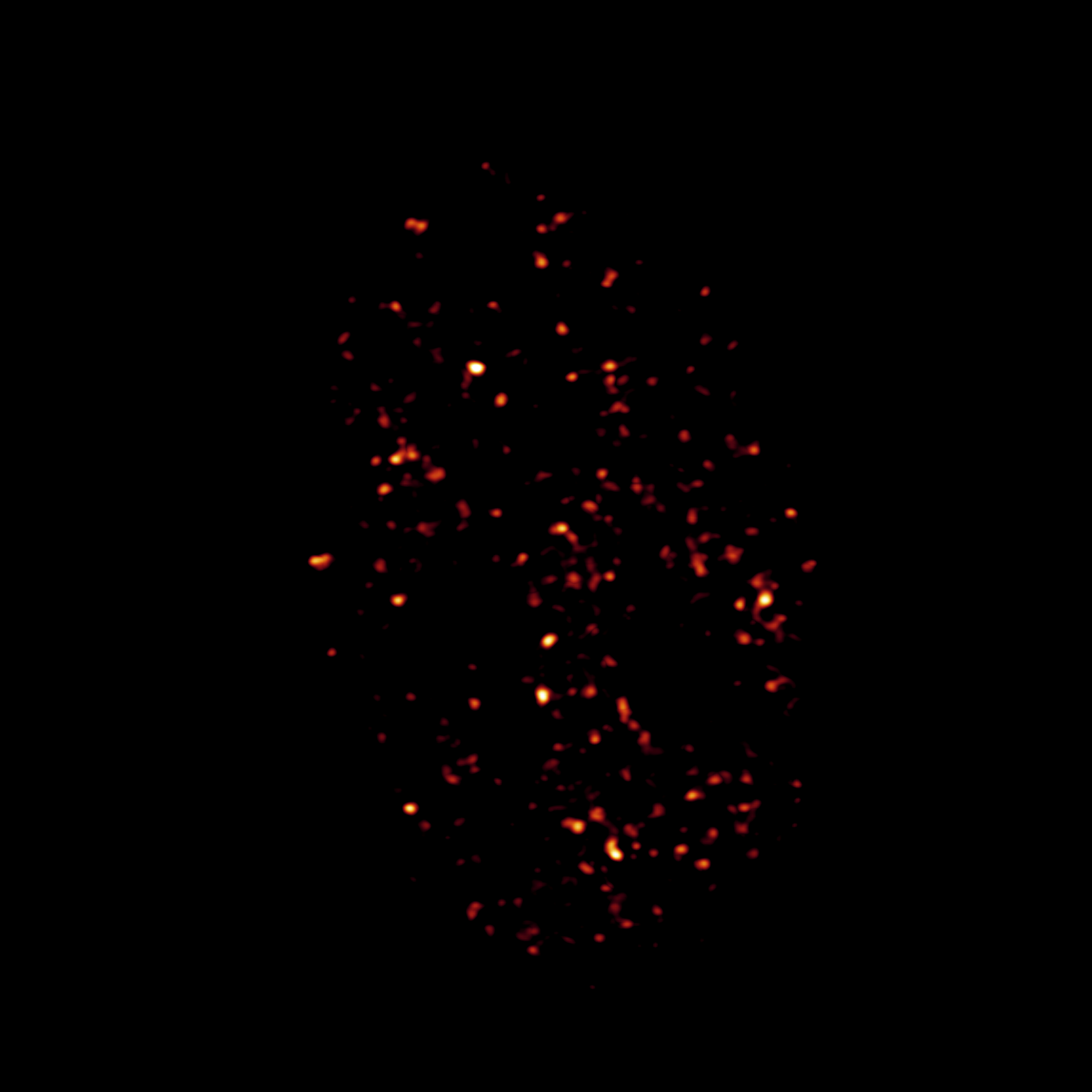

Supplement: Supplementary file 8 — Source data Fig. 4 [file 44319_2024_274_MOESM8_ESM.zip › Figure 4/4B/siSUN2_30 min.tif]

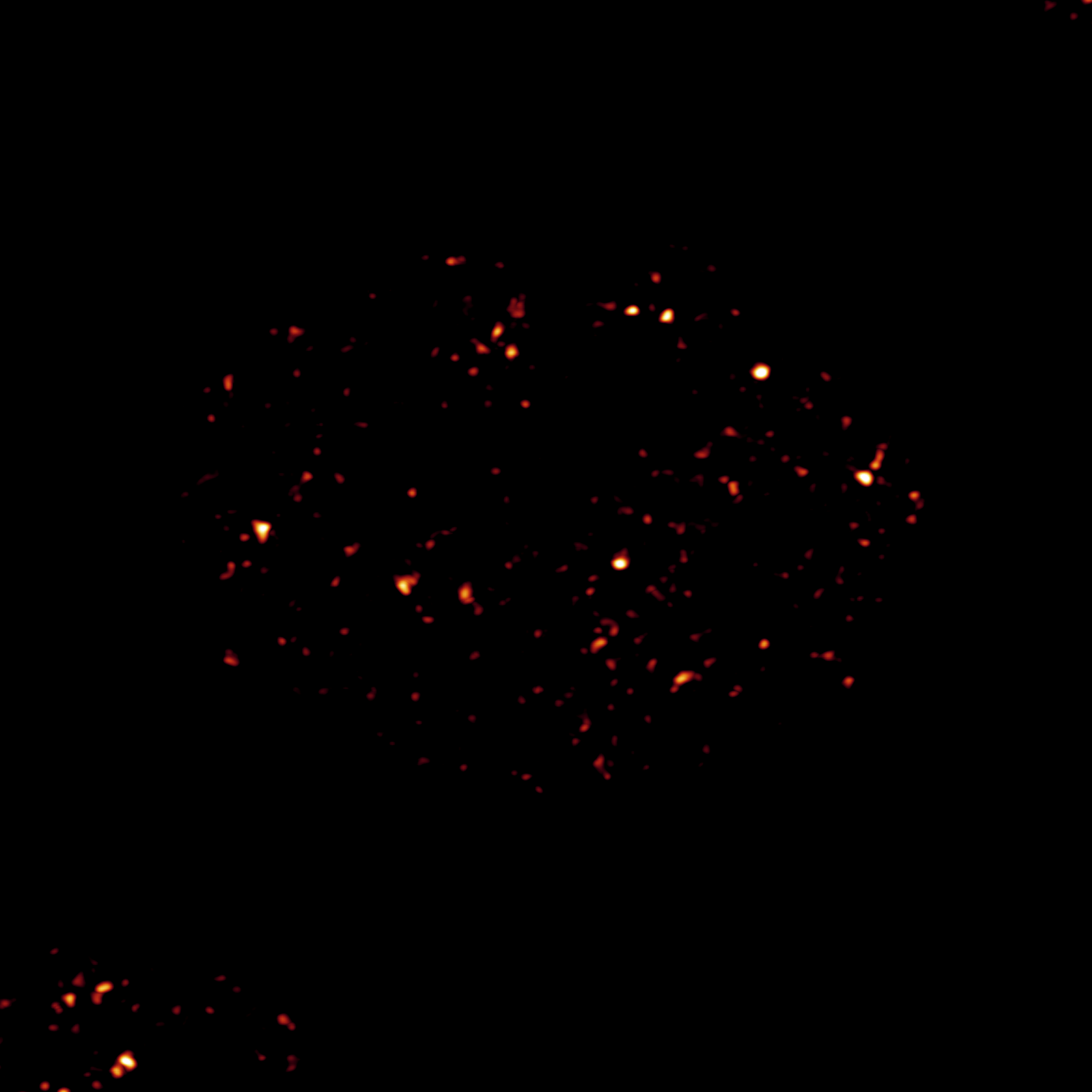

Supplement: Supplementary file 8 — Source data Fig. 4 [file 44319_2024_274_MOESM8_ESM.zip › Figure 4/4B/siSUN2_5 min.tif]

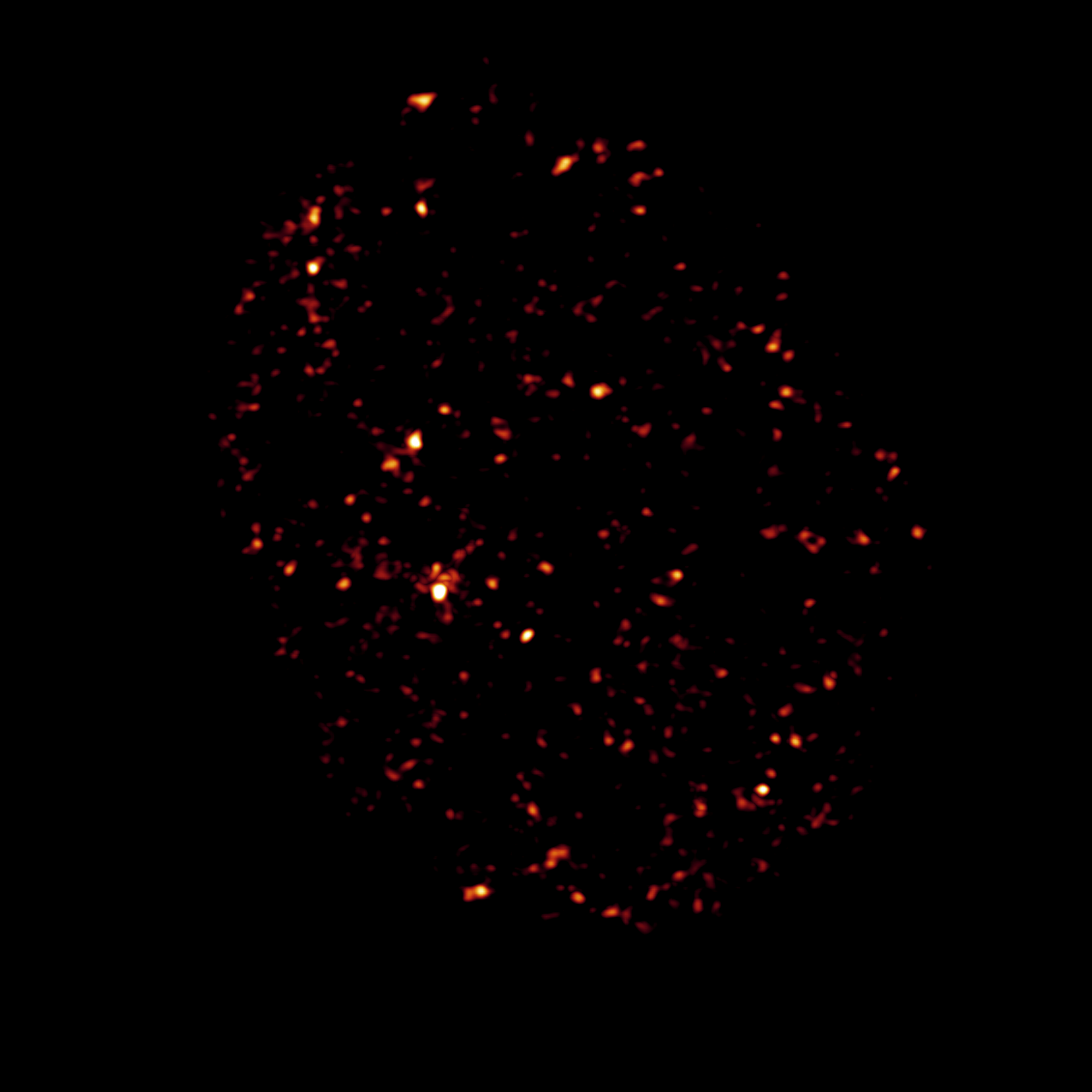

Supplement: Supplementary file 9 — Source data Fig. 5 [file 44319_2024_274_MOESM9_ESM.zip › Figure 5/5A/siCtrl_0 min.tif]

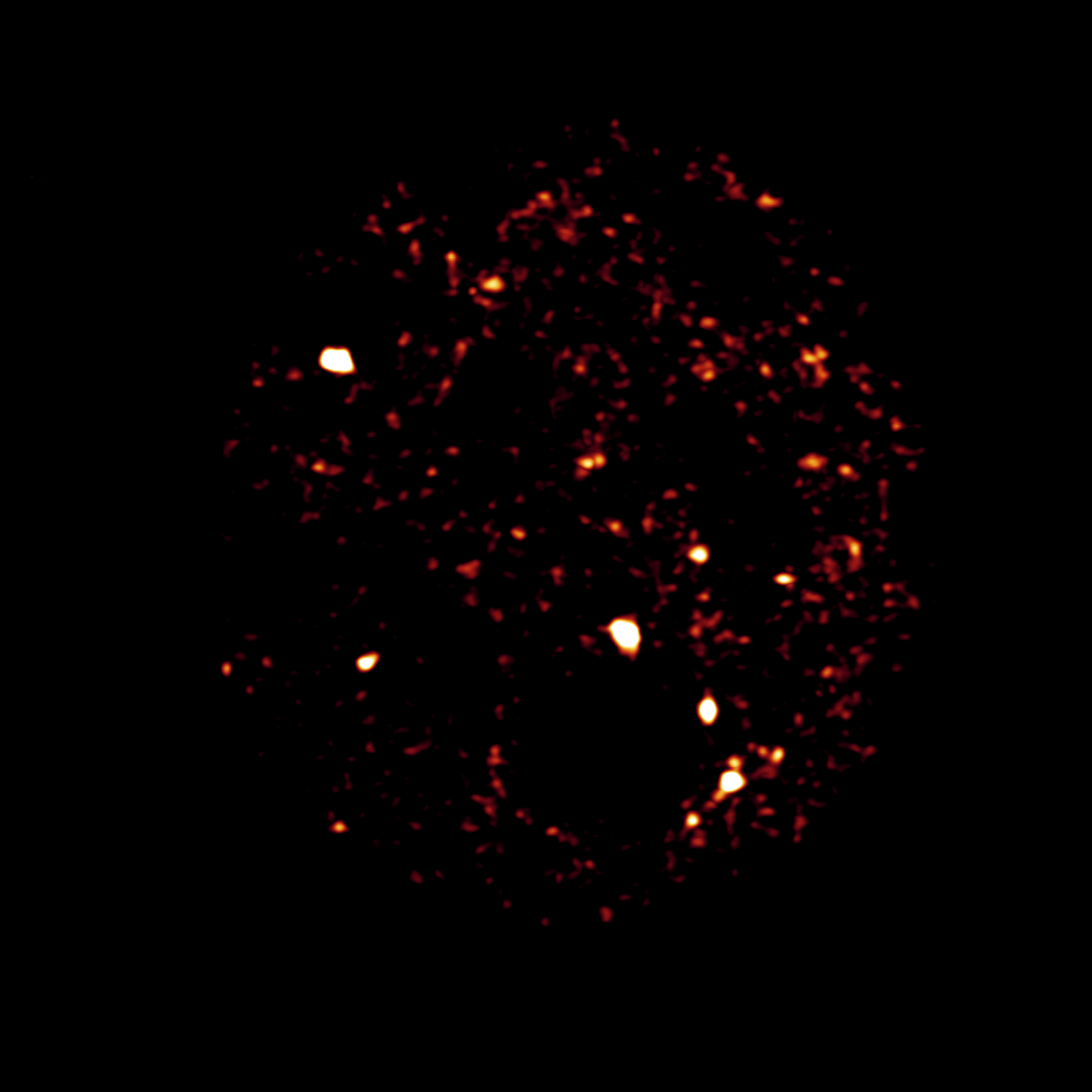

Supplement: Supplementary file 9 — Source data Fig. 5 [file 44319_2024_274_MOESM9_ESM.zip › Figure 5/5A/siCtrl_10 min.tif]

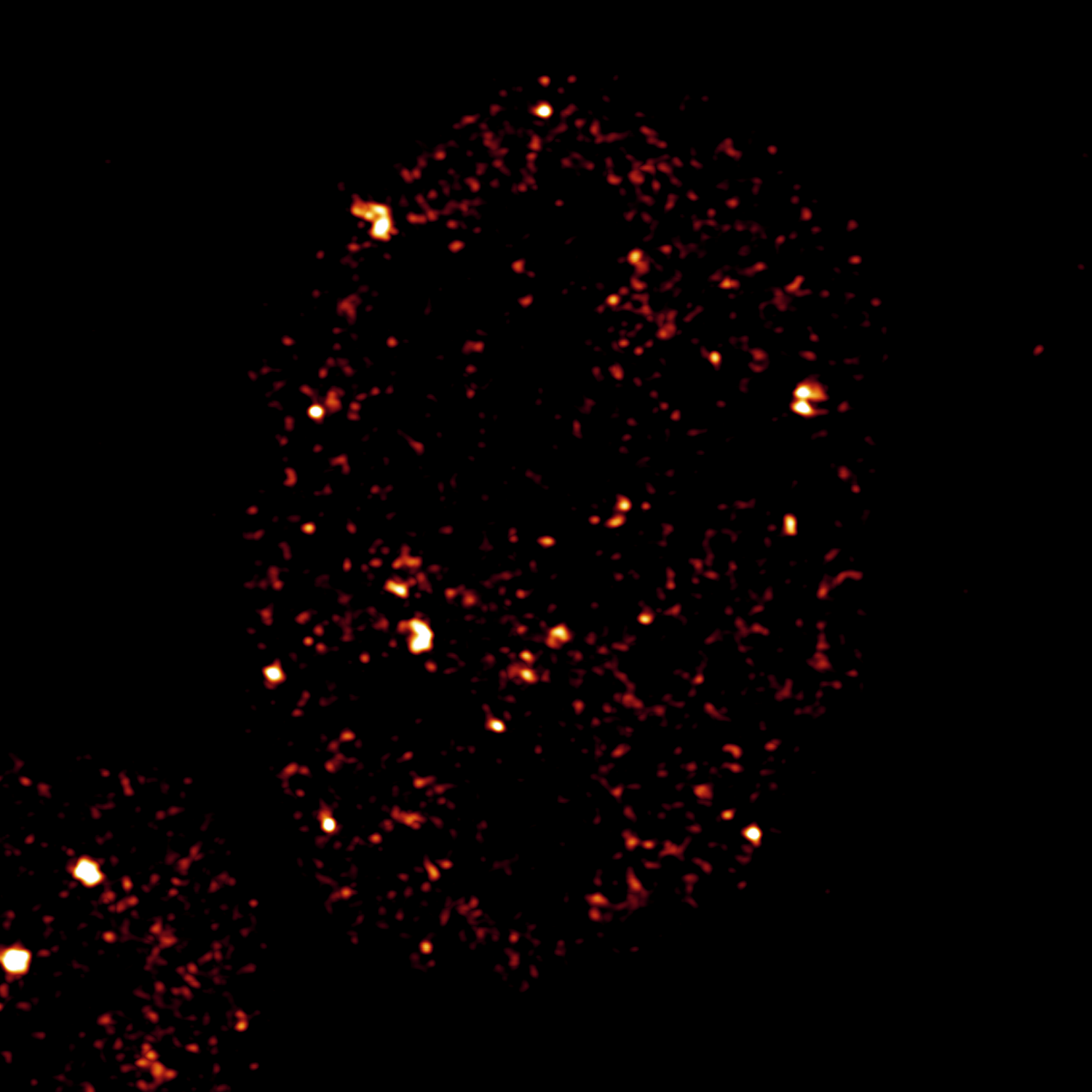

Supplement: Supplementary file 9 — Source data Fig. 5 [file 44319_2024_274_MOESM9_ESM.zip › Figure 5/5A/siCtrl_120 min.tif]

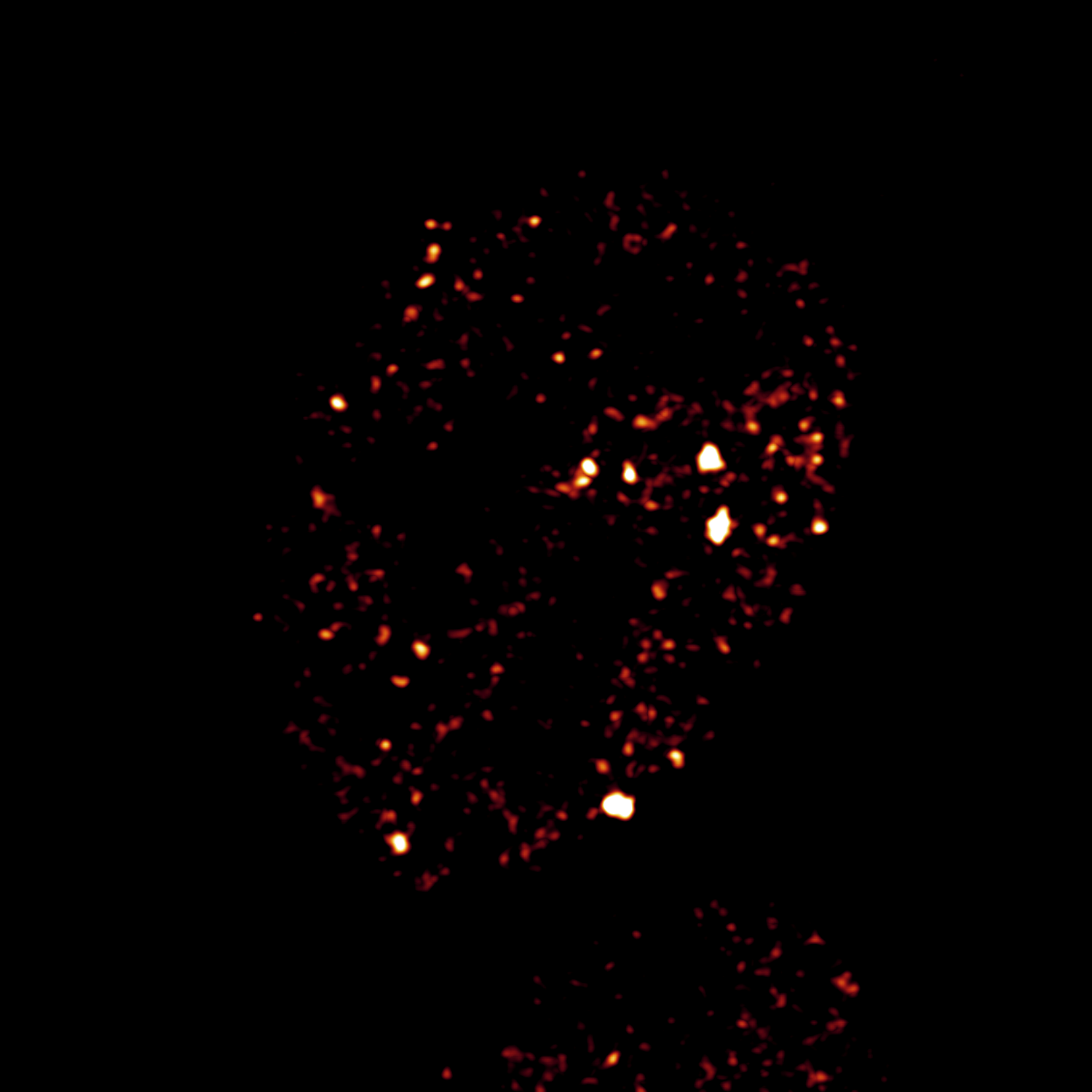

Supplement: Supplementary file 9 — Source data Fig. 5 [file 44319_2024_274_MOESM9_ESM.zip › Figure 5/5A/siCtrl_30 min.tif]

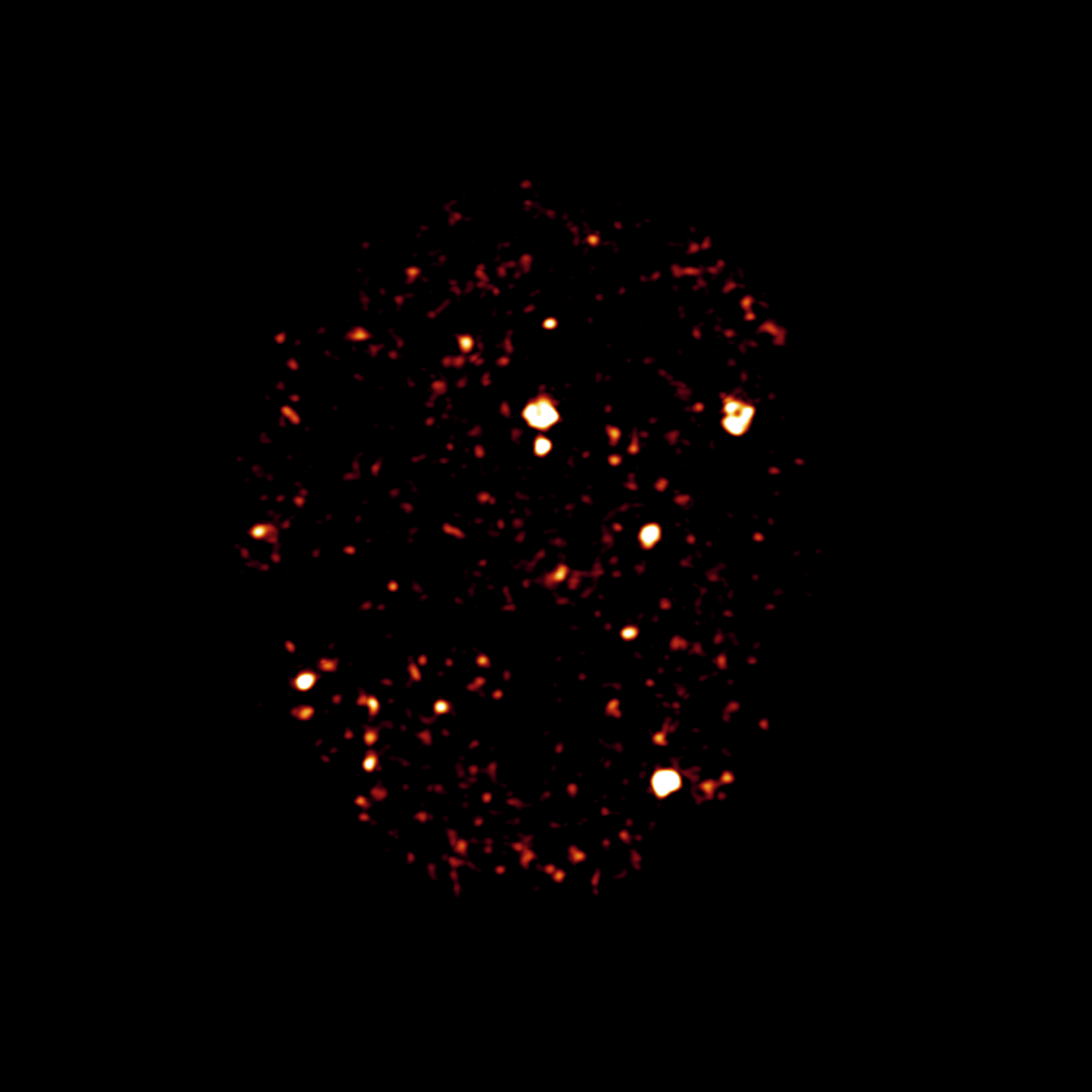

Supplement: Supplementary file 9 — Source data Fig. 5 [file 44319_2024_274_MOESM9_ESM.zip › Figure 5/5A/siCtrl_5 min.tif]

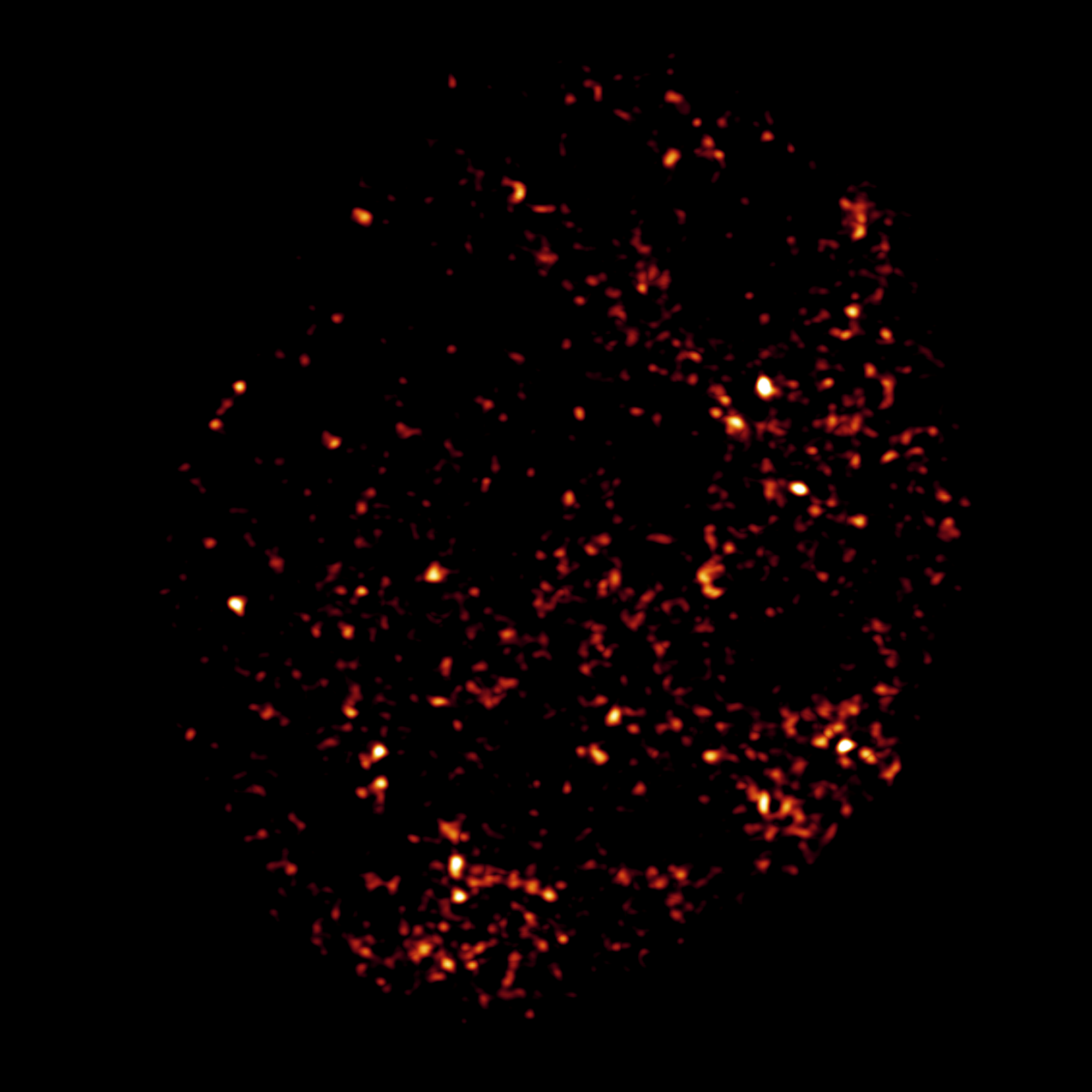

Supplement: Supplementary file 9 — Source data Fig. 5 [file 44319_2024_274_MOESM9_ESM.zip › Figure 5/5B/siINF2_0 min.tif]

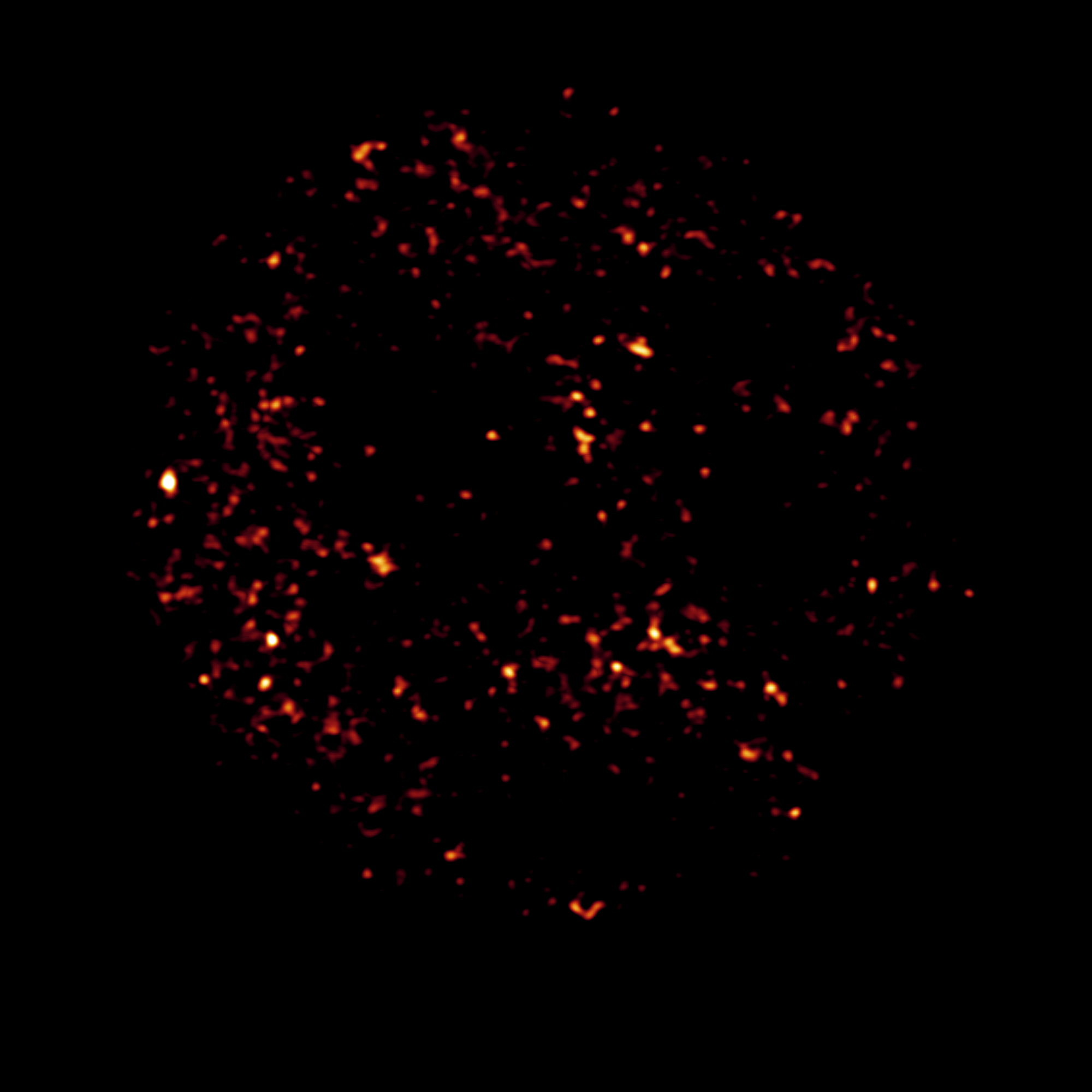

Supplement: Supplementary file 9 — Source data Fig. 5 [file 44319_2024_274_MOESM9_ESM.zip › Figure 5/5B/siINF2_10 min.tif]

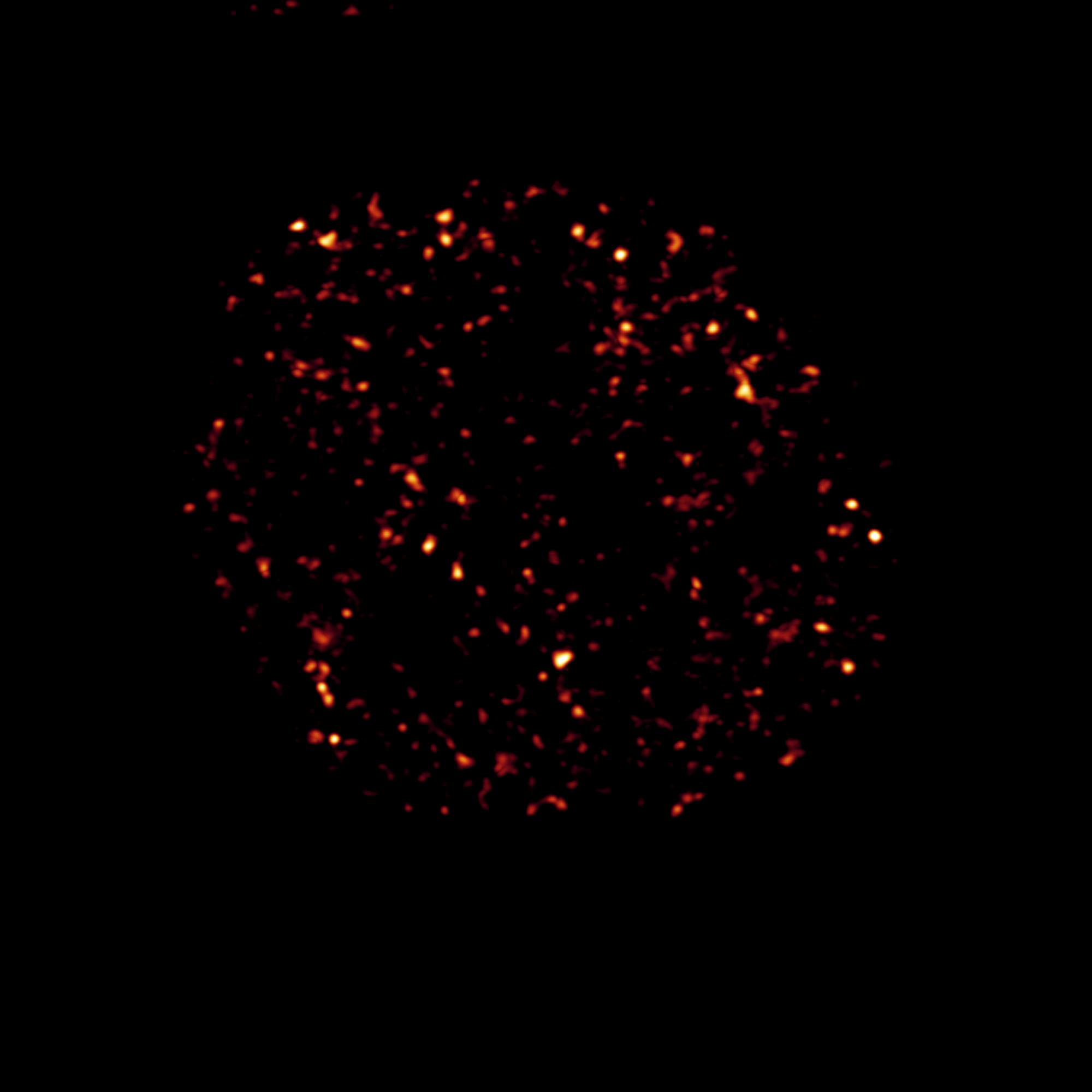

Supplement: Supplementary file 9 — Source data Fig. 5 [file 44319_2024_274_MOESM9_ESM.zip › Figure 5/5B/siINF2_120 min.tif]

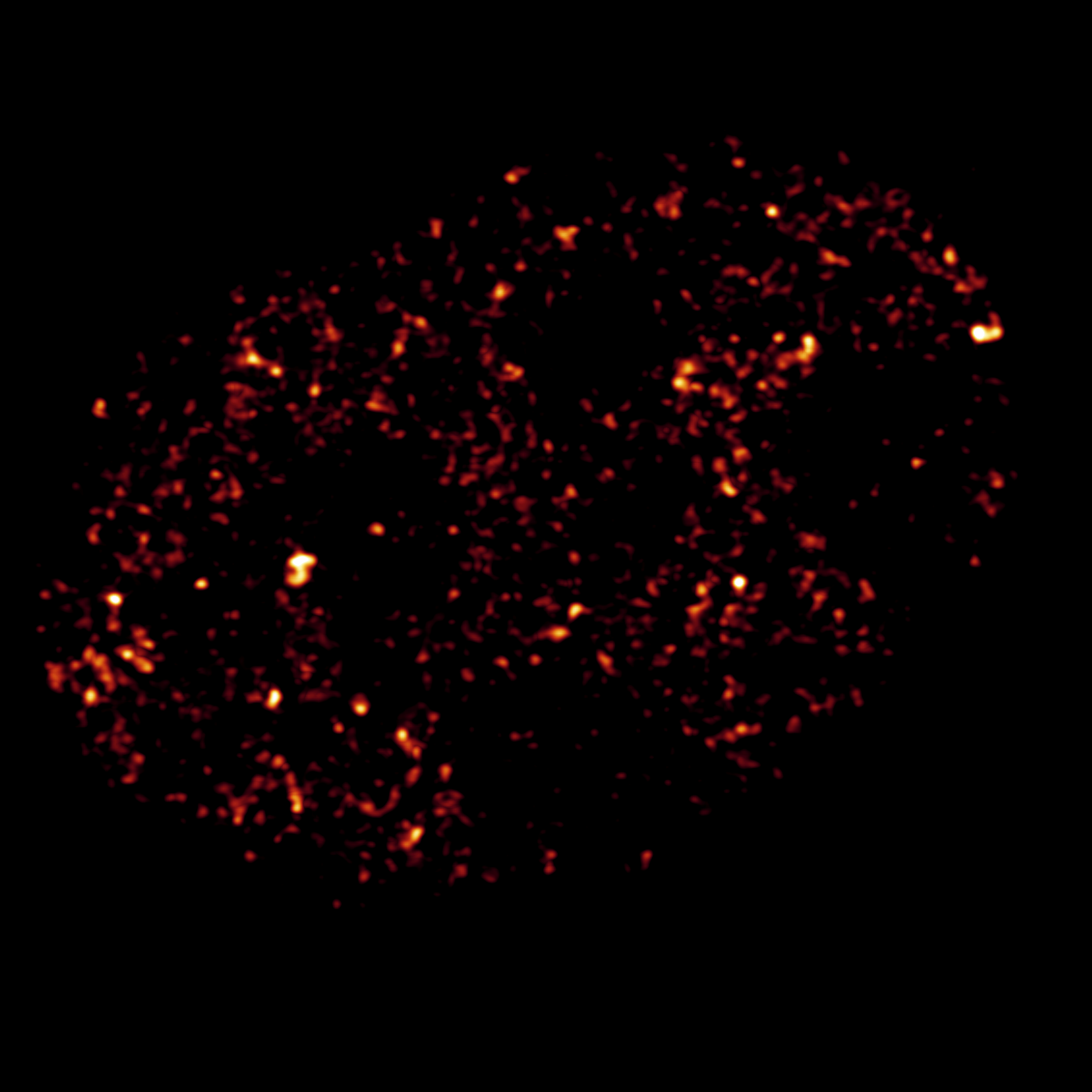

Supplement: Supplementary file 9 — Source data Fig. 5 [file 44319_2024_274_MOESM9_ESM.zip › Figure 5/5B/siINF2_30 min.tif]

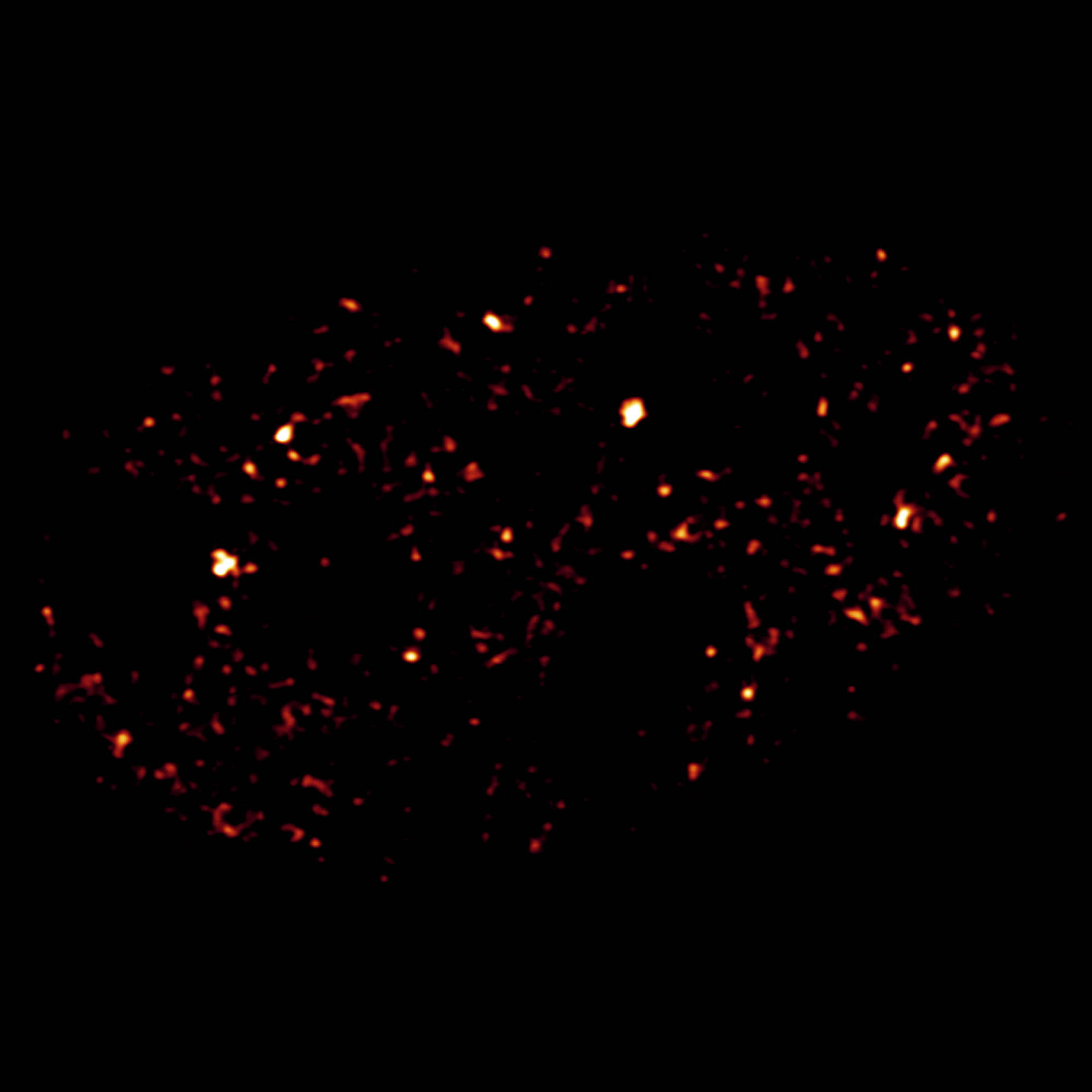

Supplement: Supplementary file 9 — Source data Fig. 5 [file 44319_2024_274_MOESM9_ESM.zip › Figure 5/5B/siINF2_5 min.tif]

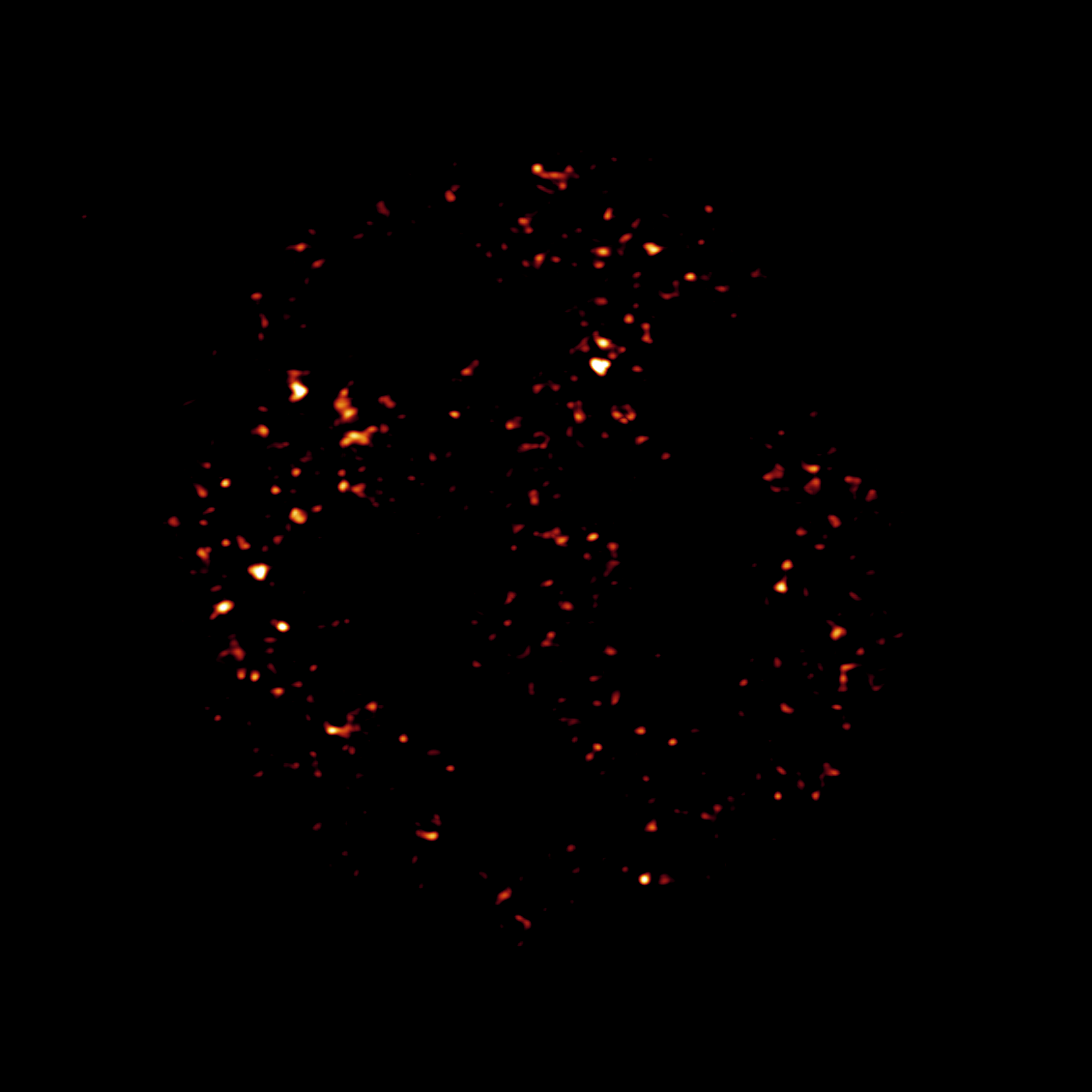

Supplement: Supplementary file 10 — Source data Fig. 6 [file 44319_2024_274_MOESM10_ESM.zip › Figure 6/6A/NLS-mSc_0 min.tif]

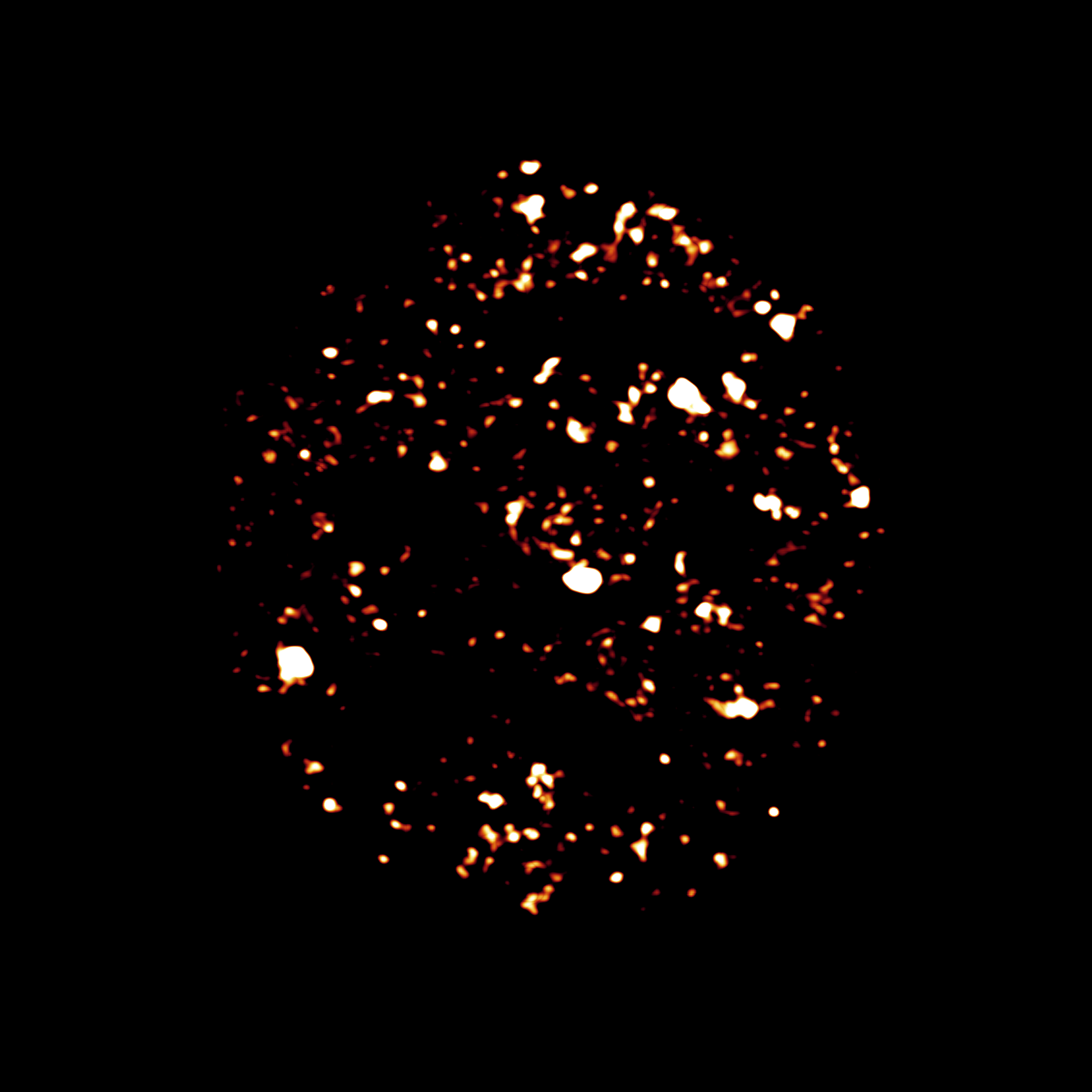

Supplement: Supplementary file 10 — Source data Fig. 6 [file 44319_2024_274_MOESM10_ESM.zip › Figure 6/6A/NLS-mSc_10 min.tif]

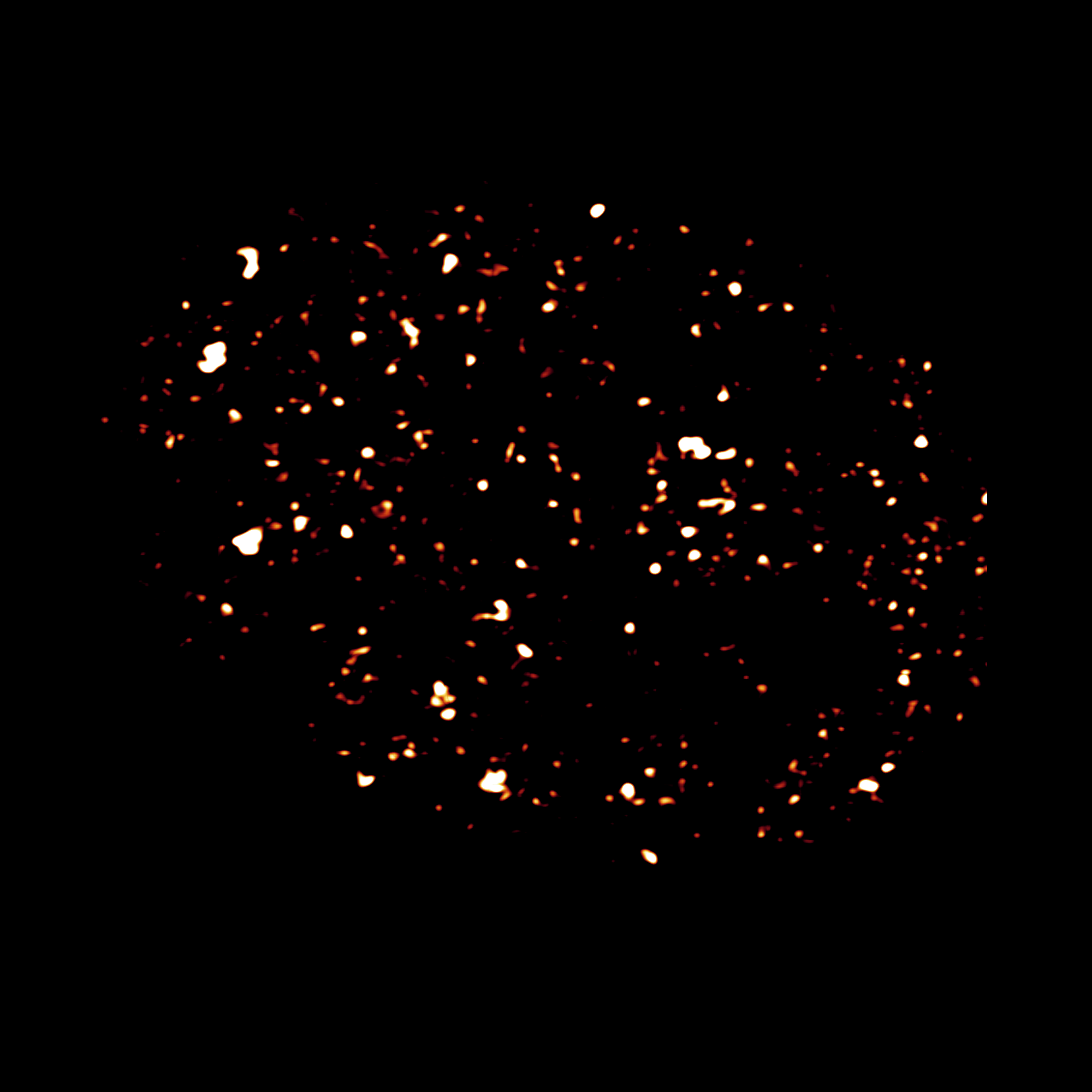

Supplement: Supplementary file 10 — Source data Fig. 6 [file 44319_2024_274_MOESM10_ESM.zip › Figure 6/6A/NLS-mSc_120 min.tif]

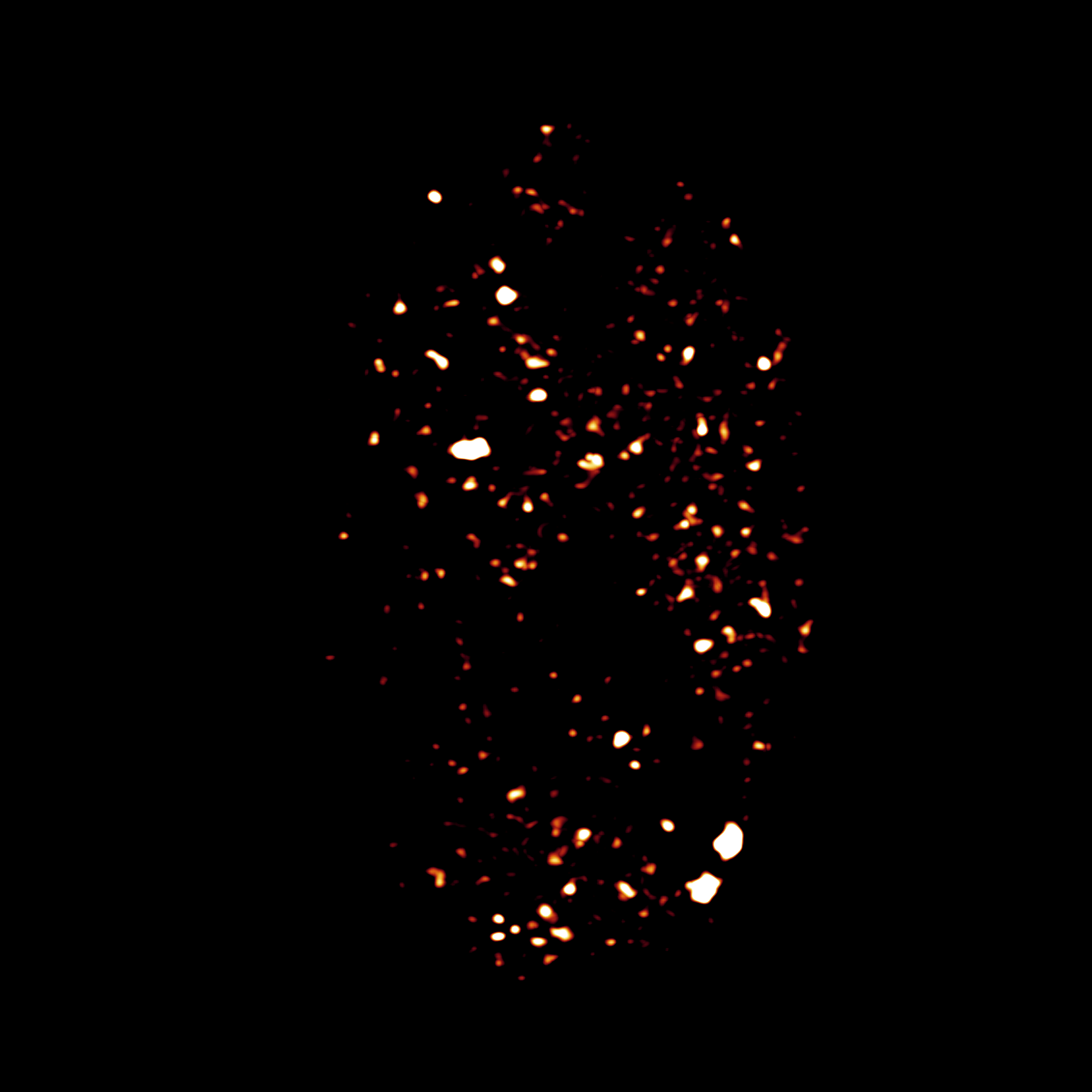

Supplement: Supplementary file 10 — Source data Fig. 6 [file 44319_2024_274_MOESM10_ESM.zip › Figure 6/6A/NLS-mSc_30 min.tif]

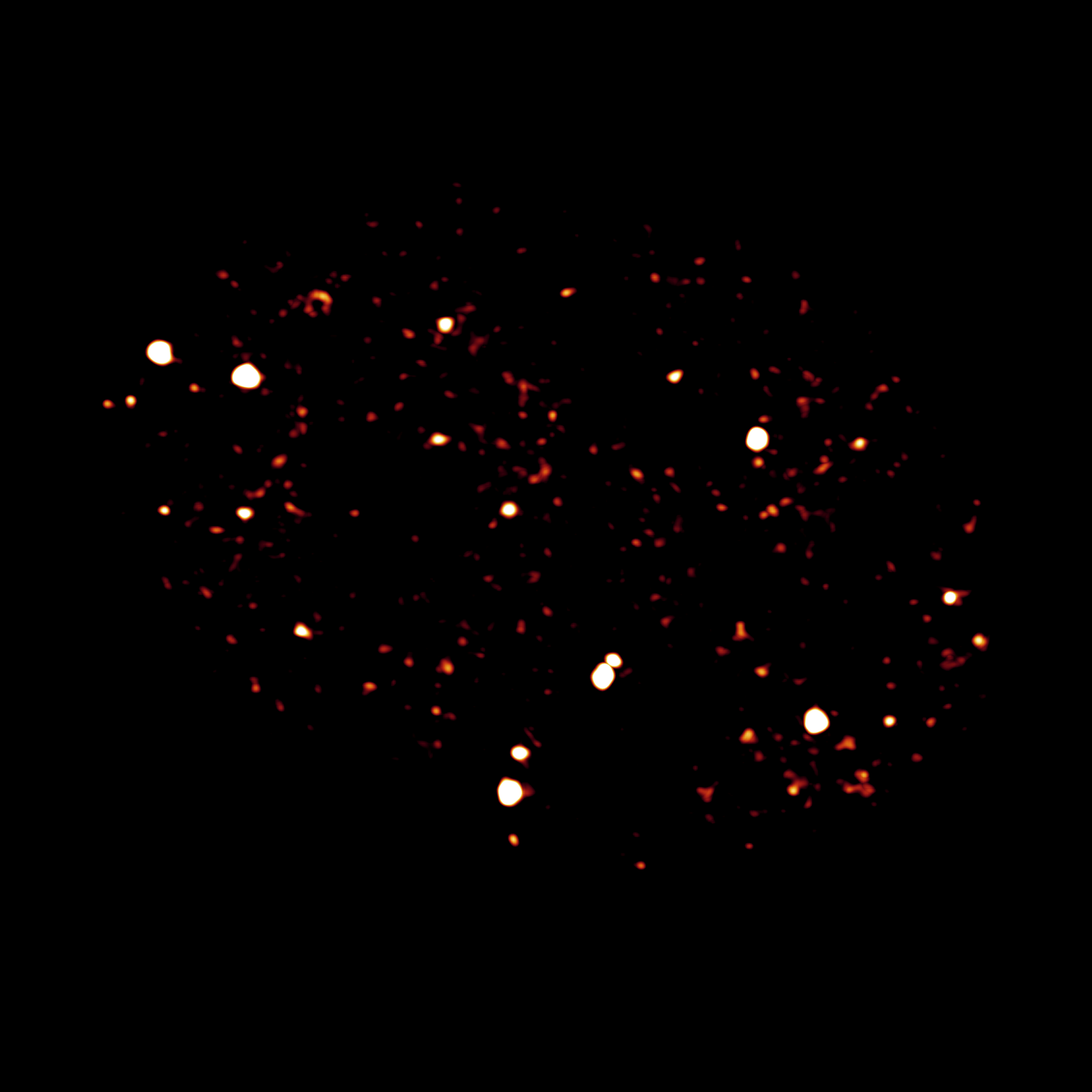

Supplement: Supplementary file 10 — Source data Fig. 6 [file 44319_2024_274_MOESM10_ESM.zip › Figure 6/6A/NLS-mSc_5 min.tif]

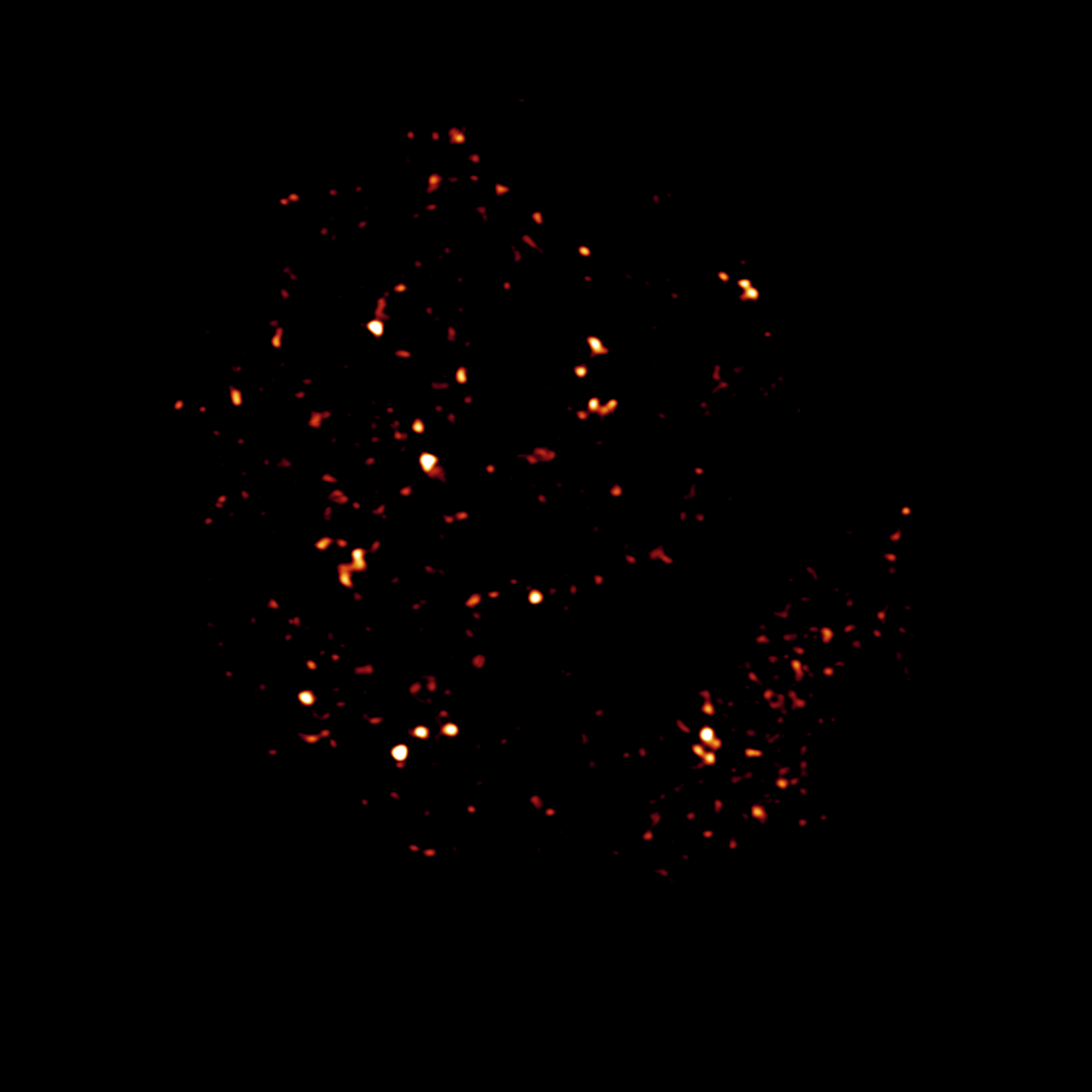

Supplement: Supplementary file 10 — Source data Fig. 6 [file 44319_2024_274_MOESM10_ESM.zip › Figure 6/6B/NLS-R62D_0 min.tif]

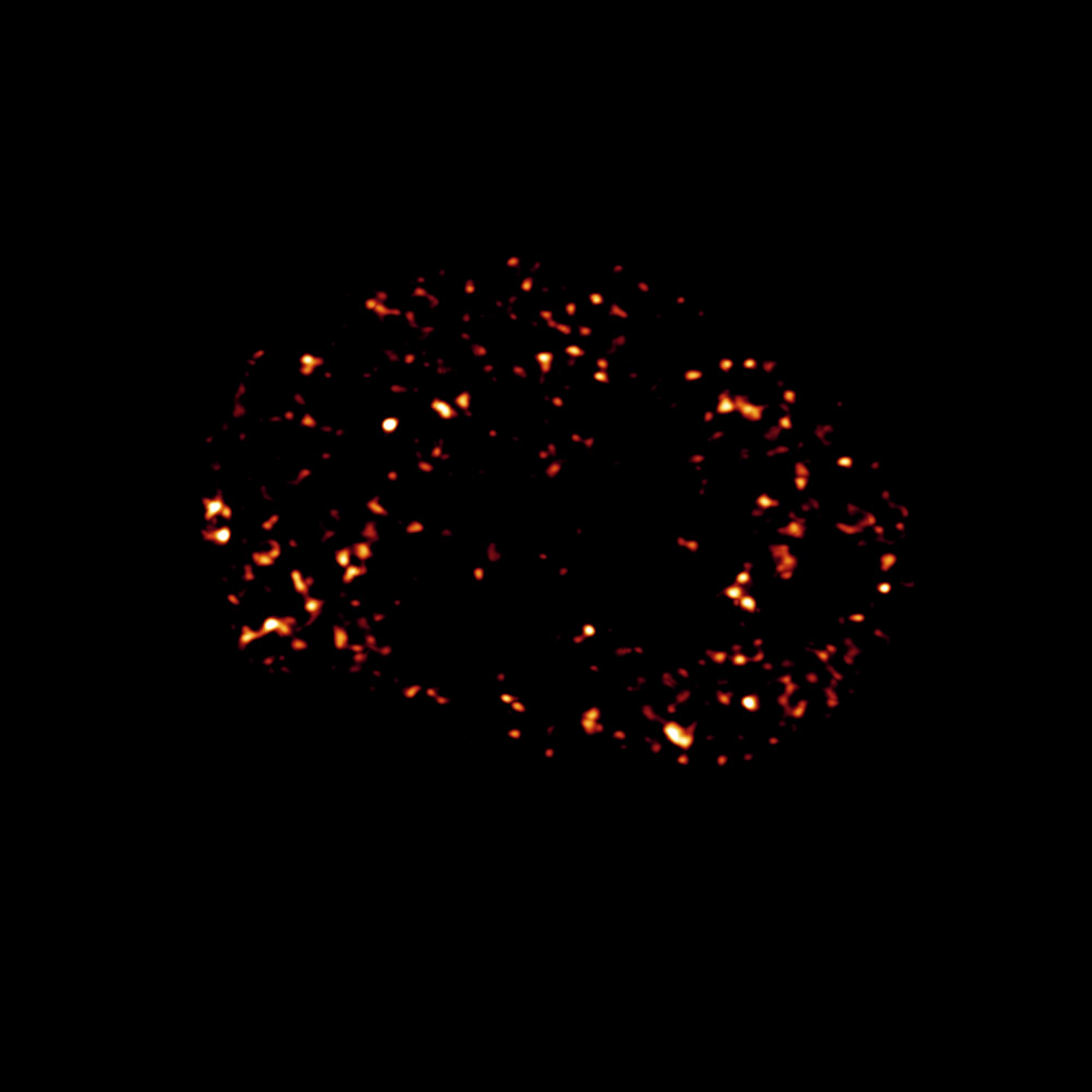

Supplement: Supplementary file 10 — Source data Fig. 6 [file 44319_2024_274_MOESM10_ESM.zip › Figure 6/6B/NLS-R62D_10 min.tif]

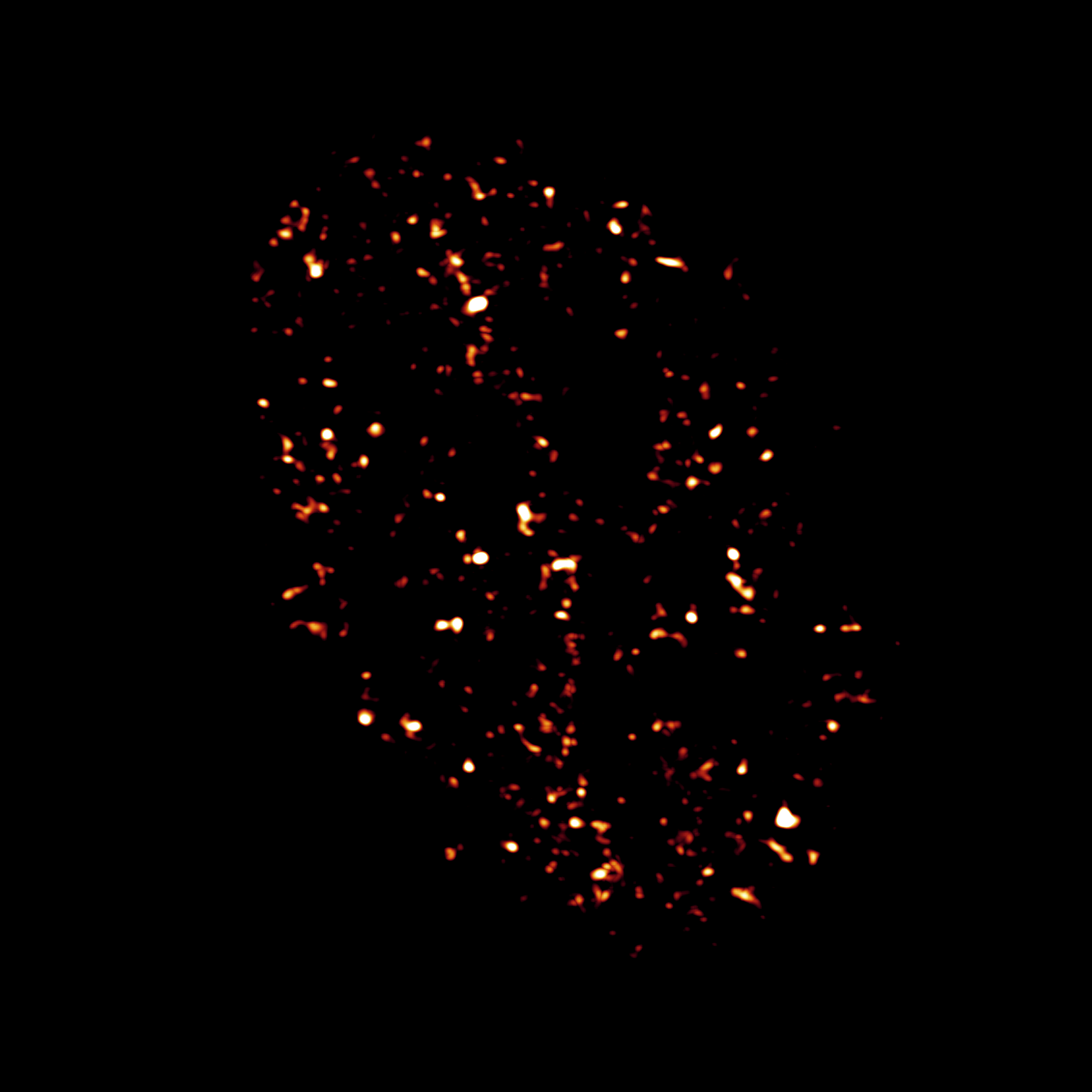

Supplement: Supplementary file 10 — Source data Fig. 6 [file 44319_2024_274_MOESM10_ESM.zip › Figure 6/6B/NLS-R62D_120 min.tif]

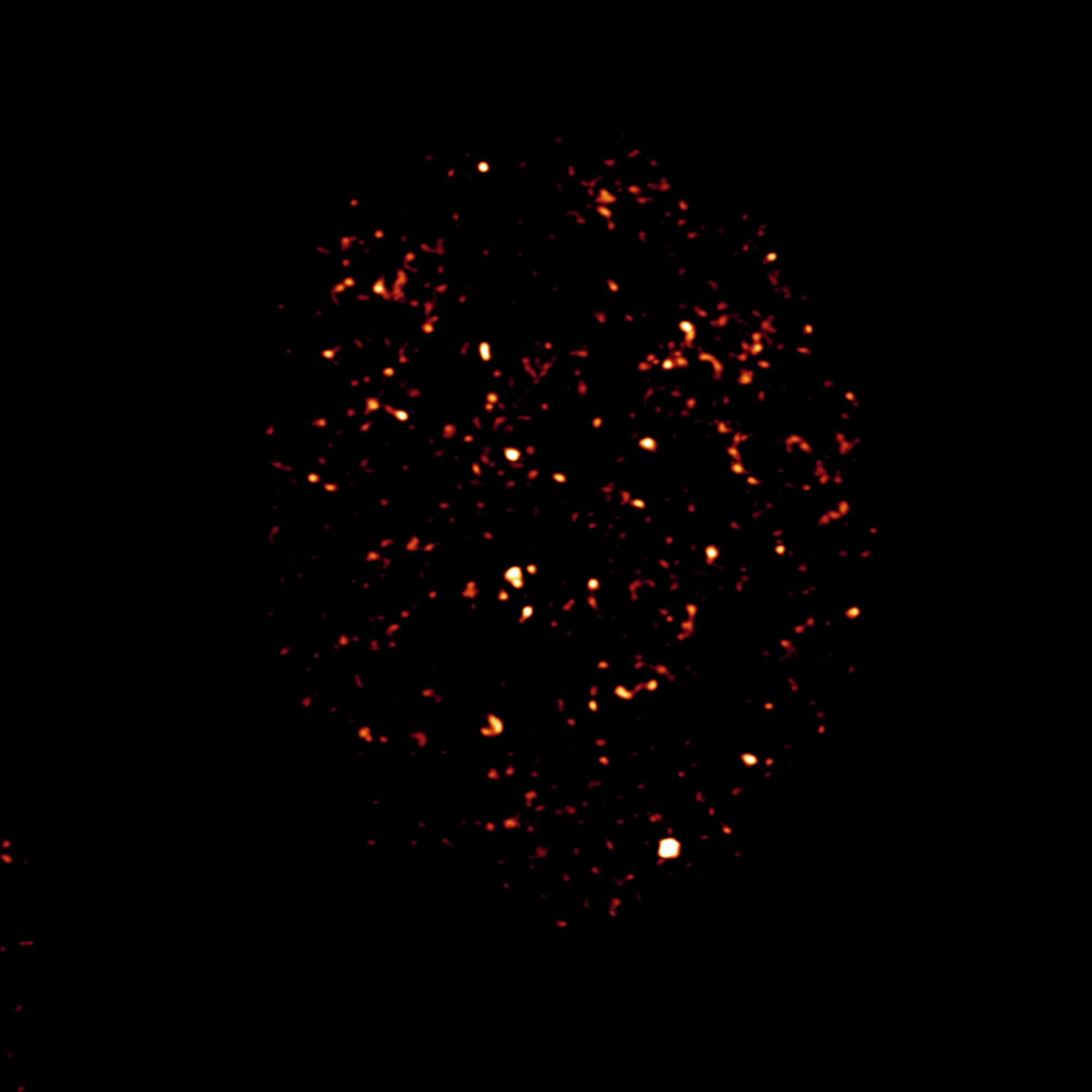

Supplement: Supplementary file 10 — Source data Fig. 6 [file 44319_2024_274_MOESM10_ESM.zip › Figure 6/6B/NLS-R62D_30 min.tif]

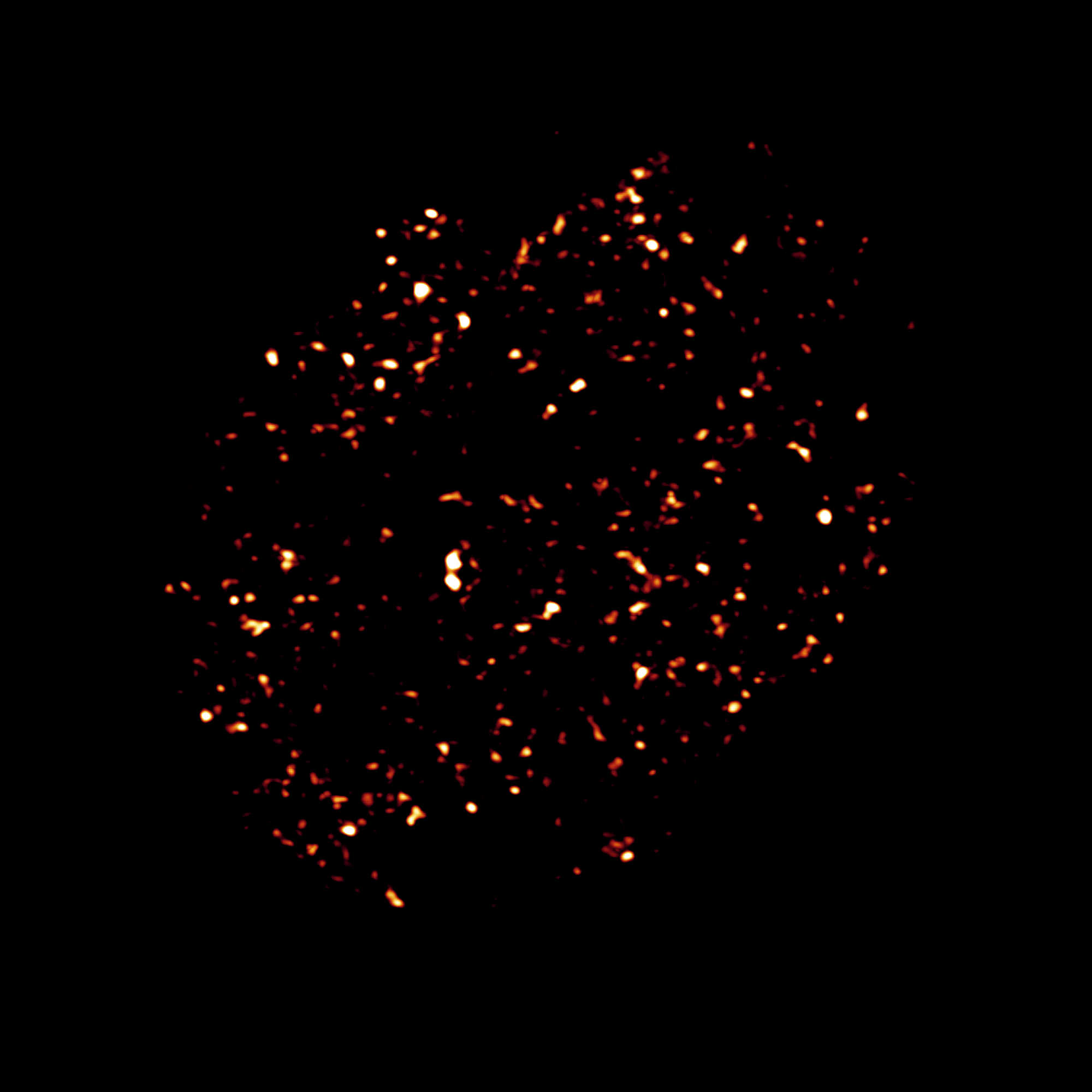

Supplement: Supplementary file 10 — Source data Fig. 6 [file 44319_2024_274_MOESM10_ESM.zip › Figure 6/6B/NLS-R62D_5 min.tif]
