# Supplementary material for: SUN2 mediates calcium-triggered nuclear actin polymerization to cluster active RNA polymerase II
Source: EMBO Rep. 2024 Sep 24;25(11):9. doi: 10.1038/s44319-024-00274-8 (PMC11549082; doi:10.1038/s44319-024-00274-8)
Supplement: Supplementary file 11 — Expanded View Figures [file 44319_2024_274_MOESM11_ESM.pdf]

Expanded View Figures

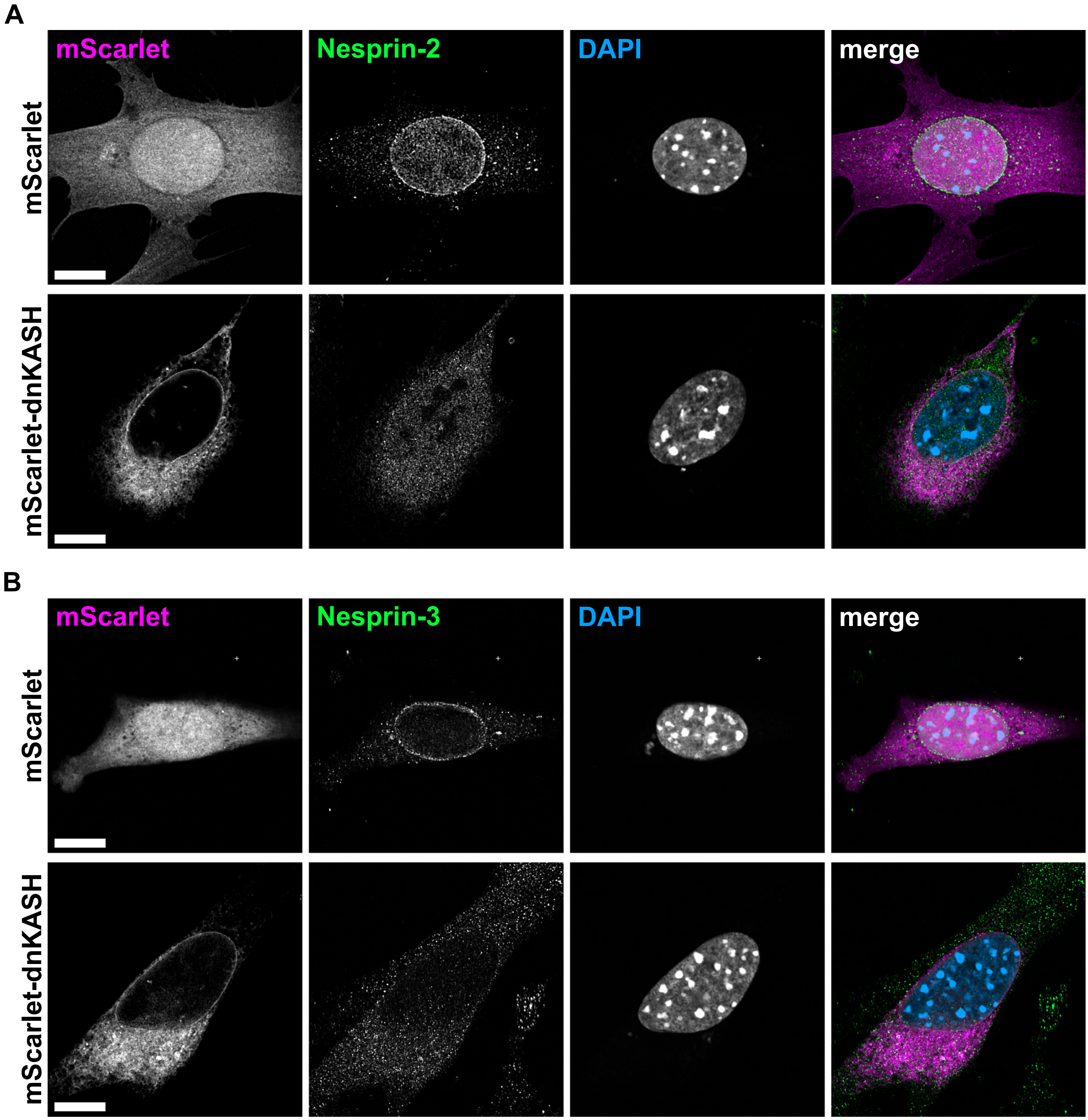

Figure EV1. Dominant-negative nesprin construct displaces endogenous nesprins from nuclear envelope.

(**A**, **B**) Immunofluorescence images of NIH3T3 cells transiently expressing mScarlet (magenta) or mScarlet-dnKASH (magenta). Cells were stained for (**A**) nesprin2 (green) or (**B**) nesprin3 (green) and DNA (DAPI, blue). scale bar = 10 µm.

© The Author(s)

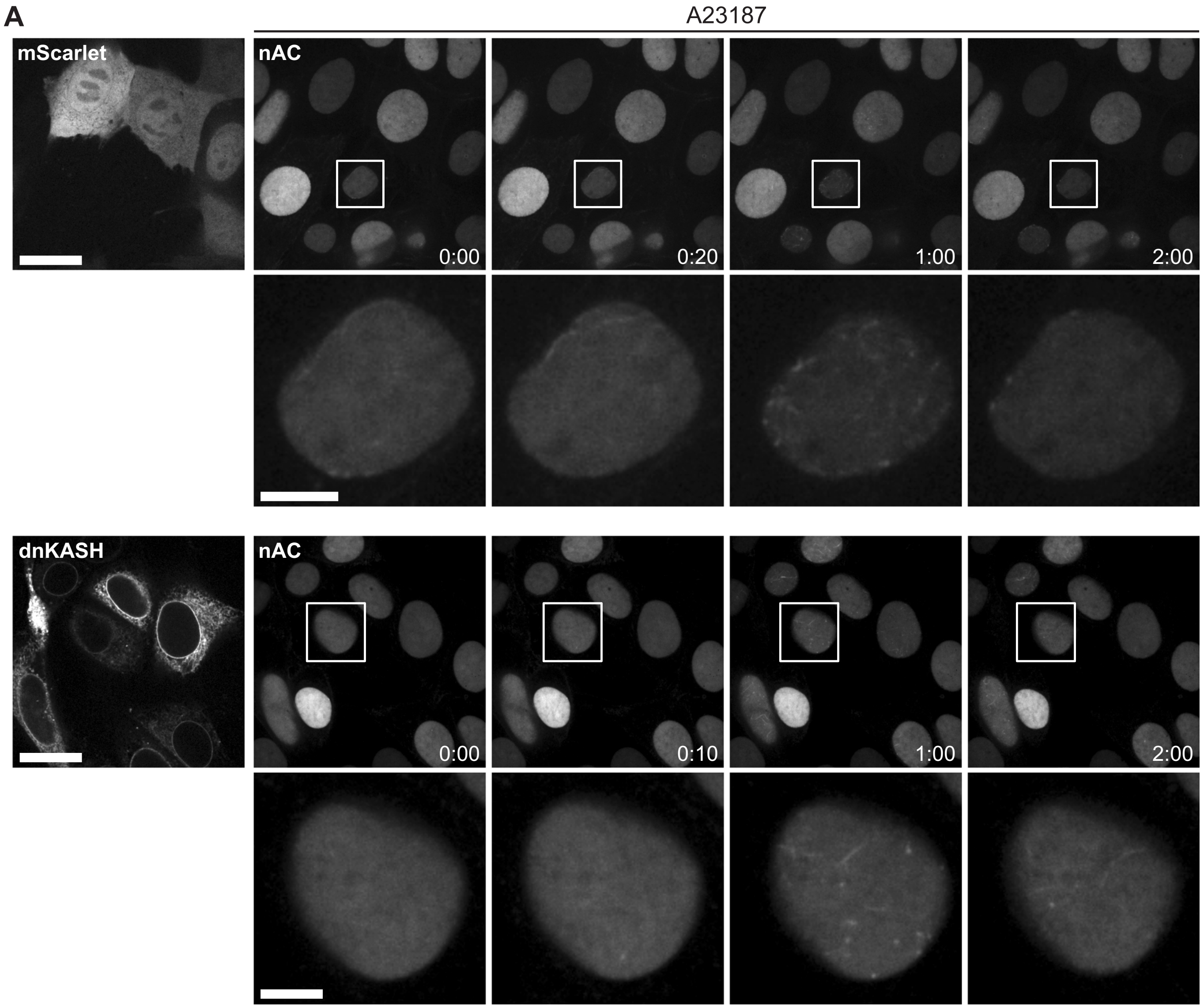

Figure EV2. Calcium transients trigger nuclear actin assembly independent of the mechanotransduction function of SUN2.

(**A**) Representative spinning disc confocal slices of NIH3T3 fibroblasts stably expressing nAC-tagGFP. Cells were transfected with mScarlet-tagged dominant-negative KASH (dnKASH) or mScarlet empty vector and stimulated with 1 μM A23187 to induce rapid nuclear actin assembly. Cells were quantified for positive events as analyzed in Fig. 1F. White boxes indicate magnified areas shown below the overview images. Scale bar = 20 μm (overview) or 5 μm (zoom). (min:s after drug treatment).

 © The Author(s)

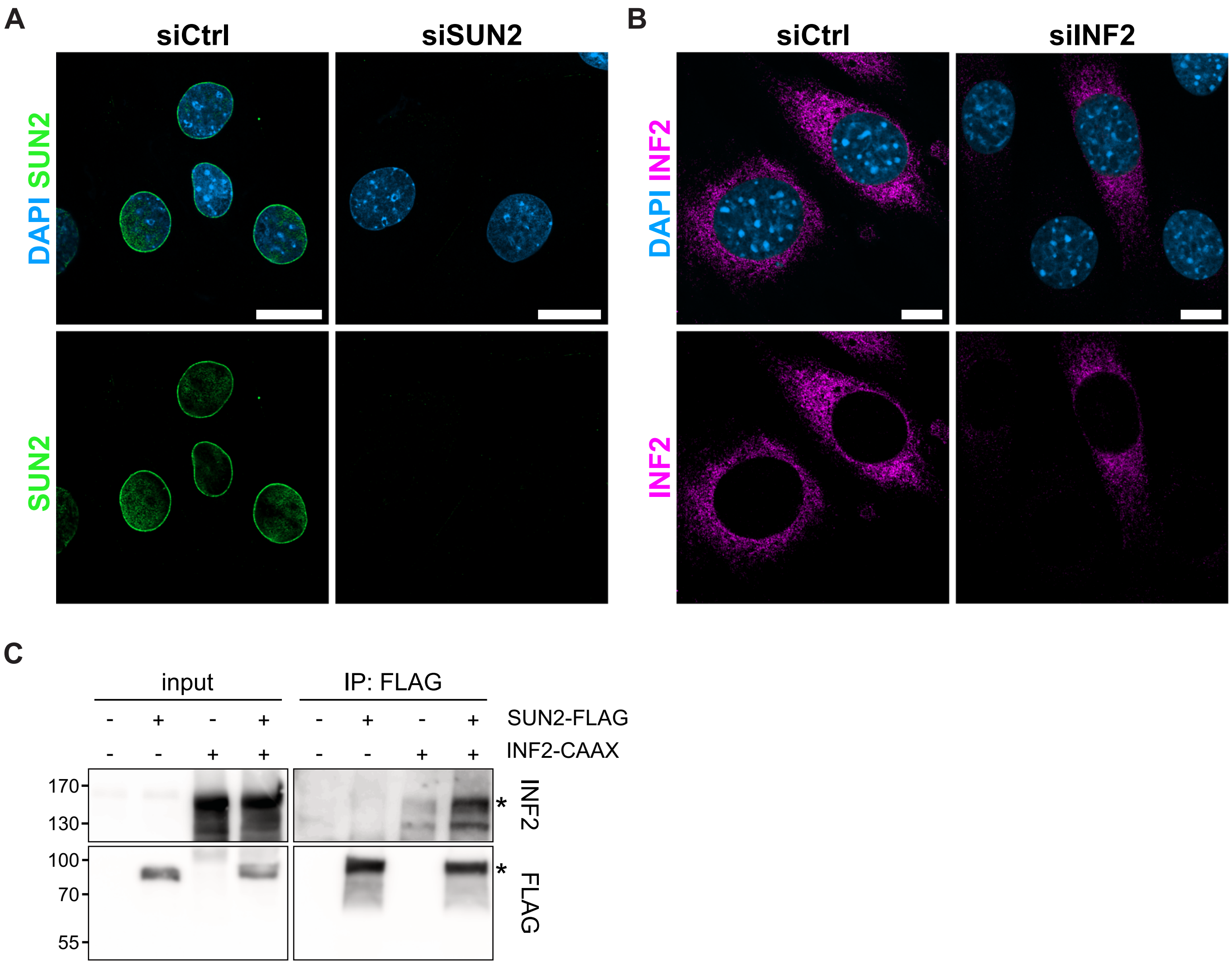

Figure EV3. Immunostaining showing antibody specificity and Co-IP of SUN2 and INF2.

(**A**) Immunostaining of endogenous SUN2 in NIH3T3 cells reveals antibody specificity as well as efficient knockdown of SUN2 protein upon transfection with SUN2 targeting siRNA. Scale bar = 20 µm. (**B**) Immunostaining of endogenous INF2 in NIH3T3 cells reveals antibody specificity as well as efficient knockdown of INF2 protein upon transfection with INF2 targeting siRNA. Scale bar = 10 µm. (**C**) Co-immunoprecipitation of HEK293T cells expressing SUN2-FLAG and INF2-CAAX using anti-FLAG beads. Asterisks indicate co-immunoprecipitated INF2 (upper right panel) and SUN2-FLAG (lower right panel). Associated INF2 was detected using an anti-INF2 antibody.

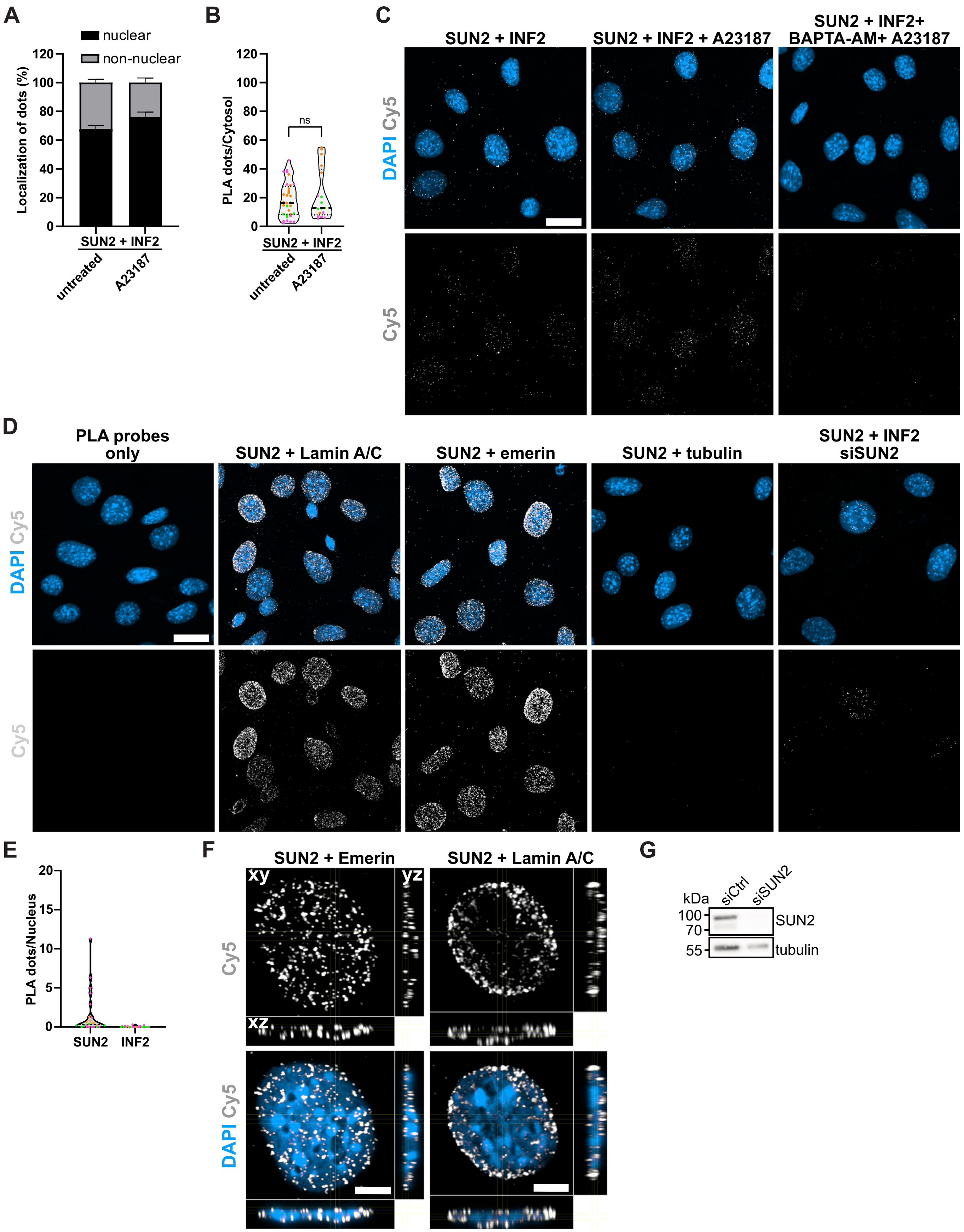
A
nuclear
non-nuclear
Localization of dots (%)
SUN2 + INF2
untreated
A23187
B
PLA dots/Cytosol
ns
SUN2 + INF2
untreated
A23187
C
SUN2 + INF2
SUN2 + INF2 + A23187
SUN2 + INF2+ BAPTA-AM+ A23187
DAPI Cy5
Cy5
D
PLA probes only
SUN2 + Lamin A/C
SUN2 + emerin
SUN2 + tubulin
SUN2 + INF2 siSUN2
DAPI Cy5
Cy5
E
PLA dots/Nucleus
SUN2
INF2
F
SUN2 + Emerin
SUN2 + Lamin A/C
xy
yz
xz
Cy5
DAPI Cy5
G
kDa
siCtrl
siSUN2
100
70
55
SUN2
tubulin

 © The Author(s)

Figure EV4. PLA control experiments show specificity of applied technique.

(**A**) Cellular distribution of detected PLA signals. Each bar represents mean +/− SEM from at least three independent experiments. (**B**) Quantification of cytosolic PLA signal reveals no significant increase in number upon A2387 treatment. Data are shown as violin plot with median and quartiles. Individual data points represent one field of view acquired from three independent biological replicates. two-tailed *t* test. ns, not significant. (**C**) Representative MIPs of NIH3T3 cells subjected to PLA and quantified in Fig. 3D. Discrete PLA signals (Cy5) indicate the interaction of endogenous target proteins. Scale bar = 20 µm. (**D**) Proximity ligation assay (PLA) of NIH3T3 cells using the indicated primary antibodies. PLA probes alone serve as technical control to detect nonspecific binding of PLA probes. Use of SUN2 primary antibody together with antibodies directed against the known interactors Lamin A/C or emerin serve as positive control. Primary antibody against cytoplasmic tubulin serves as negative biological control. Images show representative maximum-intensity projections (MIP) of 20 confocal z-slices (0.21 µm z-distance). Discrete PLA signals (Cy5) indicate the interaction of endogenous target proteins within the nucleus (DAPI). Scale bar = 20 µm. (**E**) Quantification of PLA signal (dots/nucleus) from technical controls using indicated primary antibodies alone. Data are shown as violin plot with median and quartiles. Individual data points represent one field of view acquired from three independent biological replicates. (**F**) Orthogonal optical cross section reconstructed from a Z-scan through a NIH3T3 cell nucleus stained for DAPI and PLA dots (Cy5) between SUN2 and emerin or SUN2 and Lamin A/C. Scale bar = 5 µm. (**G**) Immunoblot showing siRNA-mediated SUN2 knockdown efficiency of cells subjected to PLA (Fig. 3A,B).

© The Author(s)
